# C−C Cross‐Couplings from a Cyclometalated Au(III) C${}^{\wedge }$ N Complex: Mechanistic Insights and Synthetic Developments

**DOI:** 10.1002/chem.202102668

**Published:** 2021-08-28

**Authors:** Riccardo Bonsignore, Sophie R. Thomas, Mathilde Rigoulet, Christian Jandl, Alexander Pöthig, Didier Bourissou, Giampaolo Barone, Angela Casini

**Affiliations:** ^1^ Chair of Medicinal and Bioinorganic Chemistry Department of Chemistry Technical University of Munich Lichtenbergstr. 4 85748 Garching b. München Germany; ^2^ School of Chemistry Cardiff University Main Building Park Place CF10 3AT Cardiff UK; ^3^ CNRS/Université Paul Sabatier Laboratoire Hétérochimie Fondamentale et Appliquée (LHFA, UMR 5069) 118 Route de Narbonne 31062 Toulouse Cedex 09 France; ^4^ Catalysis Research Center & Department of Chemistry Technical University of Munich Ernst-Otto-Fischer Str. 1 85748 Garching b. München Germany; ^5^ Dipartimento di Scienze e Tecnologie Biologiche, Chimiche e Farmaceutiche Università degli Studi di Palermo Viale delle Scienze, Edificio 17 90128 Palermo Italy

**Keywords:** cross-coupling, gold cyclometalated complexes, NMR spectroscopy, organometallics, reductive elimination

## Abstract

In recent years, the reactivity of gold complexes was shown to extend well beyond π‐activation and to hold promises to achieve selective cross‐couplings in several C−C and C−E (E=heteroatom) bond forming reactions. Here, with the aim of exploiting new organometallic species for cross‐coupling reactions, we report on the Au(III)‐mediated C(sp^2^)−C(sp) occurring upon reaction of the cyclometalated complex [Au(C^CH2^N)Cl_2_] (**1**, C^CH2^N=2‐benzylpyridine) with AgPhCC. The reaction progress has been monitored by NMR spectroscopy, demonstrating the involvement of a number of key intermediates, whose structures have been unambiguously ascertained through 1D and 2D NMR analyses (^1^H, ^13^C, ^1^H‐^1^H COSY, ^1^H‐^13^C HSQC and ^1^H‐^13^C HMBC) as well as by HR‐ESI‐MS and X‐ray diffraction studies. Furthermore, crystallographic studies have serendipitously resulted in the authentication of zwitterionic Au(I) complexes as side‐products arising from cyclization of the coupling product in the coordination sphere of gold. The experimental work has been paralleled and complemented by DFT calculations of the reaction profiles, providing valuable insight into the structure and energetics of the key intermediates and transition states, as well as on the coordination sphere of gold along the whole process. Of note, the broader scope of the cross‐coupling at the Au(III) C^CH2^N centre has also been demonstrated studying the reaction of **1** with C(sp^2^)‐based nucleophiles, namely vinyl and heteroaryl tin and zinc reagents. These reactions stand as rare examples of C(sp^2^)−C(sp^2^) cross‐couplings at Au(III).

## Graphical abstract

**The mechanism of C(sp^2^)−C(sp) coupling** templated by the Au(III) cyclometalated complex [Au(C^CH2^N)Cl_2_] has been investigated. By tuning the reaction conditions and monitoring its progress by NMR, a number of intermediate species involved in the reductive elimination process could be isolated and characterised by different spectroscopic and analytical methods, including XRD, as well as by DFT studies.

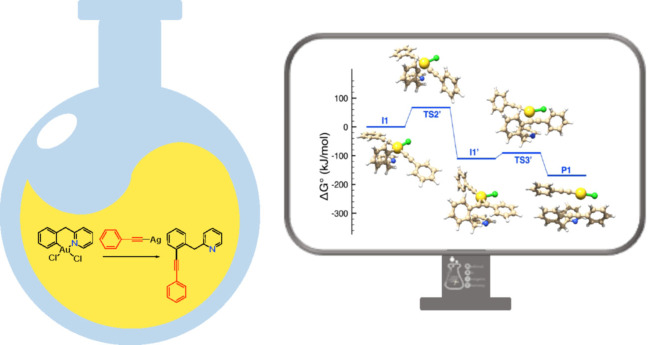

## Introduction

In the last years, homogeneous gold catalysis has evolved into a useful tool for the construction of complex and highly functionalized molecules. Gold catalysts act in most cases as efficient activators of CC multiple bonds owing to their π Lewis acidity.^[1]^ However, recently, the ability of gold complexes to undergo fundamental reactions, such as oxidative addition, reductive elimination, transmetalation and migratory insertion, has been demonstrated,^[2]^ and first insights have been gained into the underlying factors. Therefore, various examples of reductive elimination forming new C−C or C−E (E=heteroatom) bonds from two ligands at a gold centre have been reported.^[3]^ Experimental studies have shed light on the C−X (X=halide) reductive elimination from gold complexes;^[4]^ while few examples of C(sp^2^)−E bond formation through reactions with O‐ and N‐nucleophiles have also been described.^[5]^ Concerning C−S bond formation, in 2014, with the aim of introducing aryl moieties in proteins, Wong and co‐workers tackled the possibility of arylating cysteine residues starting from cyclometalated Au(III) $C^{\wedge }N$ compounds.^[6]^ Later on, investigating cysteine arylation by a series of Au(III) $C^{\wedge }N$ (${\mathrm{HC}}^{\wedge }N$ =2‐arylpyridines) complexes upon reaction with different peptidic domains, initial mechanistic insights on the reductive elimination process and structure‐activity relationships were obtained, enabling the control of the reaction in aqueous environment.^[7]^ Thus, a general reaction mechanism for cysteine arylation was proposed whereby a cysteinate residue binds Au(III) *trans* to the N of the $C^{\wedge }N$ ligand, while a second amino acid residue A coordinates to Au(III), favouring the bond breakage between the nitrogen and the metal to achieve [Au($C^{\wedge }N$ )(Cys)(A)Cl] species. Formation of the latter intermediate is crucial to promote the observed C−S cross‐coupling.^[7]^ Cysteine arylation of peptides was also reported in the case of chelate $P^{\wedge }N$ or $P^{\wedge }P$ Au(III) complexes featuring monodentate aryl ligands.^[8]^ In 2018, Bochmann and co‐workers described the reaction of [Au(III)($C^{\wedge }N^{\wedge }C$ )X] (X=Cl or SR) pincer complexes with thiols leading to the formation of aryl thioethers through cleavage of the pincer Au−C bonds by C−S reductive elimination.^[9]^ The reaction mechanism was extensively probed by kinetic experiments, showing that initial protodeauration of one Au−C bond is essential to generate a bidentate $C^{\wedge }N$ chelate, followed by the displacement of the N donor by a second equivalent of thiol, which then triggers the C−S reductive elimination.^[9]^ Of note, when using pincer complexes with X=Me, aryl or acetylide, the addition of thiols triggered C−C rather than C−S reductive elimination, although at extremely slow rates.^[9]^

The first example of Au(III)‐mediated C−P reductive elimination, was reported by Toste and co‐workers^[10]^ in 2016, while, aryl‐phosphoniums have occasionally been detected by NMR and/or mass spectrometry (MS) as side products in gold‐catalysed transformations.^[11]^ Recently we described the C_aryl_−P bond formation occurring upon reaction of Au(III) $C^{\wedge }N$ (${\mathrm{HC}}^{\wedge }N$ =2‐arylpyridines) complexes with tertiary and secondary phosphines in mild reaction conditions, including in water.^[12]^ Our mechanistic hypothesis, in line with the previously reported C−S cross‐coupling investigation on analogue compounds,^[7a]^ involves the formation of an intermediate species, whereby the N of the cyclometalated ligand has been displaced by a phosphanyl group, while the Au(III) centre maintains its favoured tetra‐coordination.^[12]^ The intermediate further evolves towards the reductive elimination product.

In this context, gold‐catalysed cross‐coupling reactions through Au^I^/Au^III^ redox cycles have also emerged as a new and complementary tool for the formation of carbon‐carbon bonds.^[13]^ In fact, the ability of gold to reductively eliminate C−C bonds from Au(III) alkyl complexes is known since the seminal work by Tobias, Kochi and Schmidbaur.^[14]^ Carbon‐carbon reductive elimination rates depend strongly on the hybridisation of the C atoms that form a new bond. In general, C(sp^3^)−C(sp^3^) and C(sp^3^)−C(sp^2^) couplings^[15]^ were found to be rather slow and required high temperatures, and in most cases went through a dissociative type mechanism. Concerning C(sp^2^)−C(sp^2^) coupling, in 2014, Toste and co‐workers reported on the formation of 4,4′‐difluorobiphenyl upon fast reductive elimination from *cis*‐diaryl‐Au(III) complexes.^[13a]^ In the same year, the group of Nevado studied a related *cis*‐diaryl‐Au(III) complex [Au(C_6_F_5_)_2_PPh_3_Cl] and reported a slow C_6_F_5_‐C_6_F_5_ reductive elimination even under elevated temperature (150 °C, 20 h, 87 % yield).^[16]^ In 2017, Kang et al. performed a systematic study on C−C reductive elimination from phosphine bis(fluoroaryl) Au(III) complexes to achieve a broad range of thermally stable bis‐aryl compounds.^[17]^ In these last examples, reductive elimination takes place from 4‐coordinated square‐planar Au(III) species.

In 2015, Corma and co‐workers reported on the catalytic homocoupling of terminal alkynes catalysed by Au(I) phosphine complexes under basic conditions in the presence of a sacrificial oxidant.^[13c]^ The study revealed that reductive elimination occurs extremely rapidly from bis‐alkynyl Au(III) intermediates of general formula [Au(alkynyl)_2_LCl] (L=PPh_3_).^[13c]^ Examples of gold‐catalysed heterocoupling^[13b]^ and homocoupling of alkynes,^[18]^ the latter leading to macrocyclization, were also reported.

**Figure 1:**
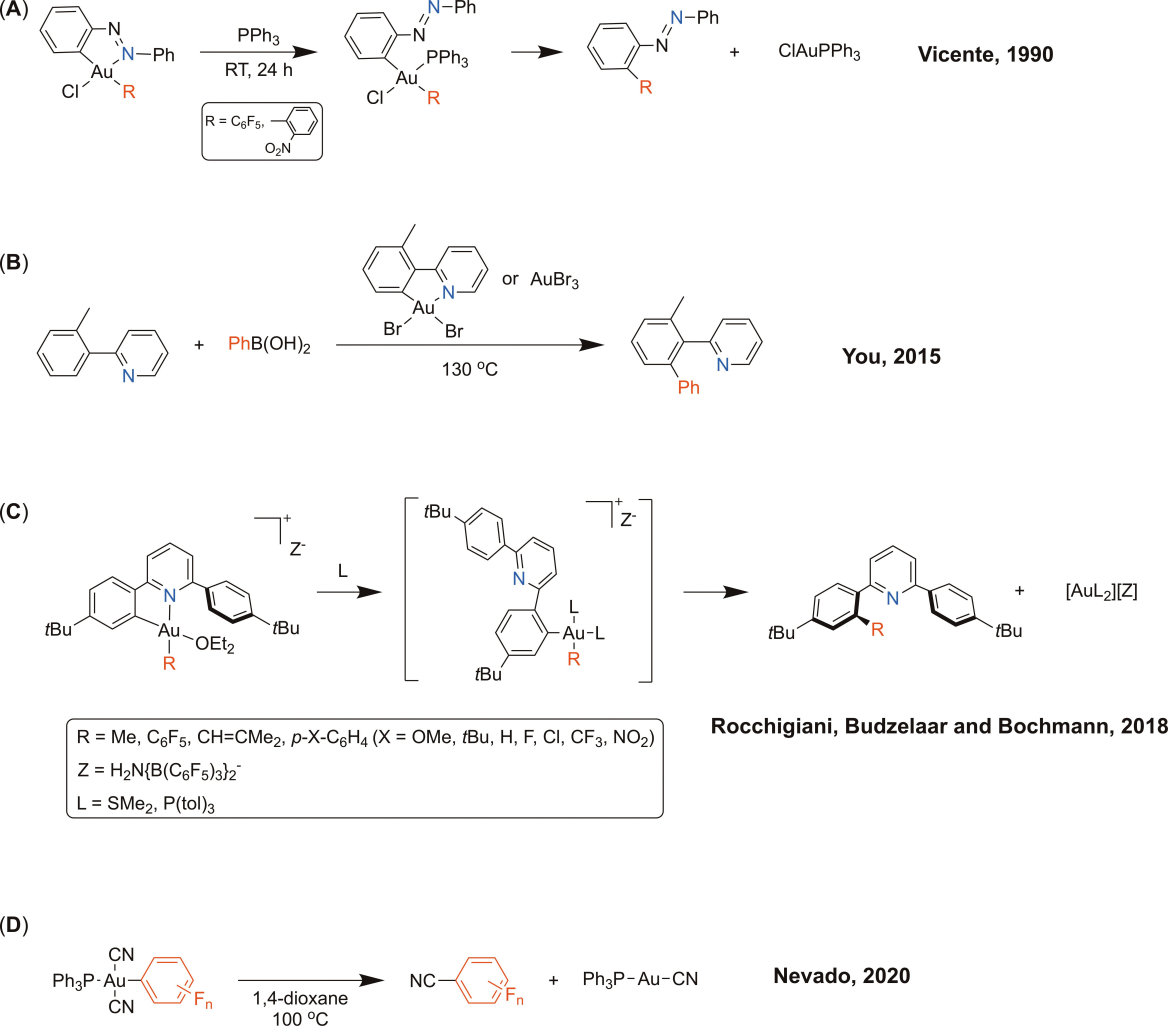
Examples of C(sp^2^)−C(sp^2^) and C(sp^2^)−C(sp^3^) cross‐couplings templated by cyclometalated Au(III) complexes (**A**–**C**) and C(sp^2^)−C(sp) cross‐coupling (**D**) from phosphine dicyanoaryl Au(III) complexes.

It is worth mentioning that developments in this relatively young area of gold‐catalysed cross‐coupling reactions have been prevented by the scarce mechanistic understanding of the individual steps along the proposed catalytic cycles. In particular, detailed information on the factors favouring the key reductive elimination process is still lacking. While it is evident that the rates of C−C coupling reactions in Au(III) complexes are strongly affected by ligand effects, with both three‐coordinate and four‐coordinate species likely involved, the lack of systematic studies does not yet allow a general picture to be drawn. In order to facilitate the mechanistic investigation, reducing the reactivity of rather labile and rapidly evolving high‐valent gold intermediates is essential; therefore, a number of examples have made use of cyclometalated Au(III) species,^[19]^ endowed with higher stability, for different types of gold‐templated C−C bond forming reactions. For example, the reductive elimination leading to aryl‐aryl cross‐coupling products in $C^{\wedge }N$ chelate Au(III) complexes was first described by Vicente et al. to be induced by the addition of PPh_3_, necessary to displace the nitrogen donor prior to the C(sp^2^)−C(sp^2^) bond‐forming step (Figure 1A).^[20]^ More recently, the phosphine‐free catalytic *ortho*‐arylation of cyclometalated 2‐arylpyridines with arylboronic acids using [Au($C^{\wedge }N$ )Br_2_] as catalyst in the presence of an oxidant was reported (Figure 1B);^[13f]^ this phosphine‐free system requires forcing conditions (130 °C). Furthermore, Rocchigiani et al. have investigated C−C reductive elimination from a range of Au($C^{\wedge }N$ ) cations obtained by protodeauration of Au(III) $C^{\wedge }N^{\wedge }C$ pincer complexes with a strong acid (Figure 1C).^[21]^ In this case, the rate of C−C cross‐coupling (aryl‐R bond formation) decreased in the sequence R=vinyl>aryl ≫C_6_F_5_>Me. Porcel and co‐workers reported on the C(sp^2^)−C(sp) cross‐coupling of aryl Au(III) complexes (resulting from the addition of diazonium salts to Au(I)) with silver acetylides,^[22]^ but the reductive elimination pathway was not investigated in detail. Despite the demonstrated efficiency of gold to trigger the formation of aryl‐alkynyl bonds, only one mechanistic study has been reported so far on C(sp^2^)−C(sp) reductive elimination, in contrast to the aforementioned investigations on C(sp^3^)−C(sp^3^) and C(sp^2^)−C(sp^2^) bond‐forming reactions. Finally, Nevado et al. studied the formation of benzonitriles from phosphine‐ligated dicyanoaryl‐Au(III) compounds (Figure 1D).^[23]^

**Scheme 1:**
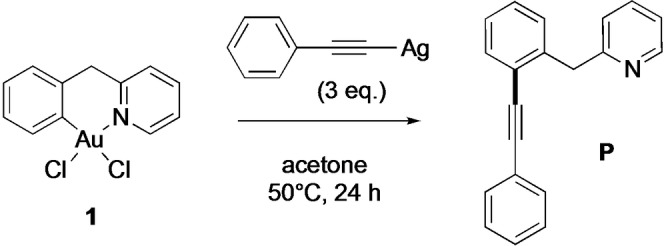
C(sp^2^)−C(sp) cross‐coupling of the cyclometalated Au(III) C^CH2^N complex **1** with AgPhCC.

In this context, in order to gain mechanistic knowledge on C−C reductive elimination at Au(III), we engaged in a detailed study of the reaction of the Au(III) $C^{\wedge }N$ complex **1** (Scheme 1) with different organometallic species. In the course of our investigations, Wong and co‐workers published the reaction of a series of cyclometalated Au(III) $C^{\wedge }N$ compounds (${\mathrm{HC}}^{\wedge }N$ =2‐arylpyridines) with phenylacetylene in the presence of a base to afford the reductive elimination product.^[24]^ Evidently, C(sp^2^)−C(sp) coupling partly outcompetes the alternative possible C(sp)−C(sp) homocoupling process, which is anyway detected in ca. 17 % yield in the applied experimental conditions.^[24]^ As previously observed in the case of the C−P and C−S cross‐coupling reactions of analogous Au(III) $C^{\wedge }N$ compounds,[^6^, ^12^] reductive elimination occurs only in the case of 6‐membered cyclometalated derivatives, and not with 5‐membered chelates, most likely due to the higher steric demand nearby the C atom of the aryl group approaching the incoming residue in the latter case.^[24]^ In this work, we shed light on the mechanism of this type of C(sp^2^)−C(sp) coupling reactions at Au(III), successfully identifying several of the intermediate species eventually leading to reductive elimination, as well as some side products. DFT studies provide invaluable support for the elucidation of the likely reaction pathways. Furthermore, the scope of the C−C coupling from Au(III) $C^{\wedge }N$ compounds is extended to C(sp^2^)−C(sp^2^) bond formation.

## Results and Discussion

### C(sp^2^)−C(sp) cross‐coupling

Initially, we probed the ability of the Au(III) cyclometalated complex [Au(C^CH2^N)Cl_2_] **1** (HC^CH2^N=2‐benzylpyridine, Scheme 1) to participate in C(sp^2^)−C(sp) cross‐coupling reactions. Upon reaction of **1** with 3 equiv. of silver phenylacetylide (AgPhCC) in acetone (25 mL) at 50 °C overnight (Scheme 1), the alkynylation product **P**, that is, a dissymmetric diarylalkyne with a pendant pyridine moiety, was obtained after purification by column chromatography with a yield of 33 %. This could be increased to 73 % by reacting with an additional equivalent of AgPhCC for a further 24 h. A side product of the reaction was found in trace amounts, that could be assigned as the homocoupled alkyne side product by ^1^H NMR spectroscopy (data not shown). The product **P** was characterised by ^1^H, ^13^C and 2D NMR spectroscopy, as well as by HR‐ESI‐MS (High‐Resolution Electrospray Ionisation Mass Spectrometry) (Figure S1–8, Supporting Information). Of note, the alkynylation product **P** is formed in the absence of Pd traces, as checked by Inductively Coupled Plasma Mass Spectrometry (ICP‐MS). The possible role of Pd contaminants in gold complexes has been pointed out in Sonogashira cross‐coupling of aryl iodides and terminal alkynes with gold complexes, see ref. [42]. Overall, and although using different reaction conditions, our results are in accordance with those recently reported by Wong et al.,^[24]^ that is, formation of alkynylation product **P** by reaction of Au(III) $C^{\wedge }N$ complex (1 equiv.) with a terminal alkyne (5 equiv.) and K_2_CO_3_ (5 equiv.) in an acetonitrile/water mixture (1 : 1) at 40 °C for 16 h.

We also attempted the reaction with the analogous 5‐membered metallacyclic complex [Au($C^{\wedge }N$ )Cl_2_] ($C^{\wedge }N$ =2‐phenylpyridine), which we previously found inactive towards both C−S and C−P cross coupling.[^7a^, ^12^] Thus, the C−C coupling was performed in an NMR tube in DMSO‐*d_6_* using 3 equiv. of AgPhCC and heated in an oil bath to 50 °C for 24 h. The NMR spectrum was then recorded directly; however, no reaction occurred (data not shown), also in line with Wong's observations.^[24]^

The reaction of **1** with AgPhCC was further challenged in different solvents. In the case of DMSO, the reaction was carried out directly in the NMR tube at lower scale under similar conditions, that is, 3 equiv. of AgPhCC, 50 °C, 24 h. In this case, the reaction yield was estimated by ^1^H NMR spectroscopy using an internal standard (see Experimental Section for details). The obtained yields of product **P** formation after purification are reported in Table 1, and show that DMSO is the most favourable solvent for the cross‐coupling reaction.

**Table 1:** C(sp^2^)−C(sp) bond formation from reaction of **1** (1 equiv.) with AgPhCC (3 equiv.) in different solvents (25 mL) maintained at 50 °C for 24 h, with their corresponding yield of product **P** formation.

Solvent	Yield
Acetone	33 %
MeOH	49 %
DMF	traces
DMSO^[a]^	70 %
Water	traces
MeCN	63 %

[a] In a NMR tube using DMSO‐*d_6_* and estimated via ^1^H NMR with internal standard.

Acetonitrile was also found to be suitable, with the 2^nd^ highest yield of product **P** formation (63 %), whilst only traces of the product could be observed when using DMF or water. In the latter case, we could argue that this was likely due to the insolubility of the reagents.

In an attempt to slow down the reaction rate and identify all the possible intermediates leading to product **P** formation, the reaction was conducted again in acetone at room temperature (see Experimental Section for details) and carefully monitored by ^1^H NMR spectroscopy.

**Figure 2:**
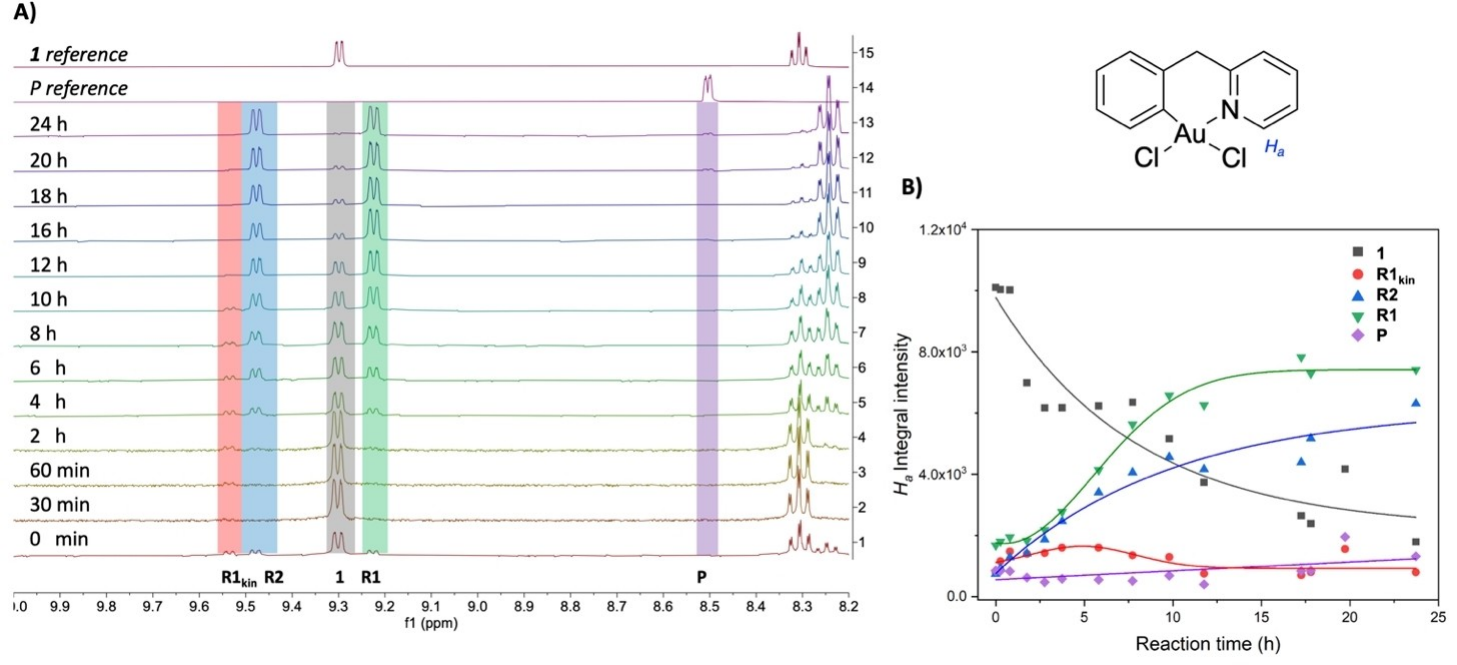
**A**) ^1^H NMR spectra (between 8.2 and 10 ppm) of the reaction between **1** (1 equiv.) and AgPhCC (2 equiv.) followed over 24 h at r.t. in acetone‐*d_6_*. The spectra of the starting material **1** and of the final purified product **P** are reported as reference. The various signals, corresponding to *H_a_* chemical shifts, are assigned to different species, **R1_kin_**, **R1** and **R2**, as depicted in Scheme 2. **B**) Evolution of the intensities of the integrals of *H_a_* chemical shifts over 24 h. Fitting lines have been included as a visual aid to follow the trend over the selected time window. The structure of **1** highlighting the proton *ortho* to the pyridyl N (*H_a_*) is included.

**Scheme 2:**
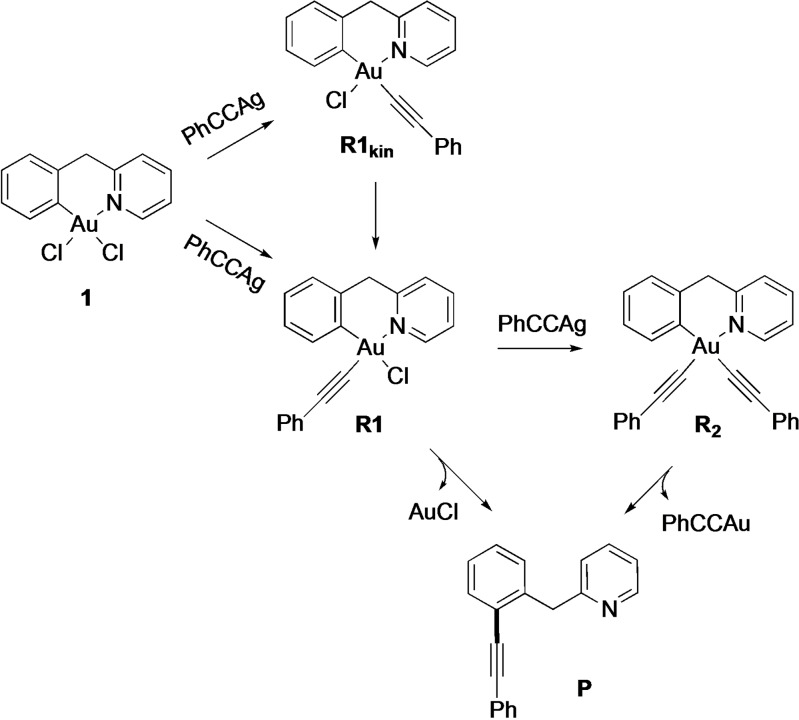
Reaction mechanism proposed to account for the C(sp^2^)−C(sp) cross‐coupling reaction templated by the Au(III) complex **1**.

The obtained results are reported in Figure 2A, B. The chemical shift of the proton *ortho* to the pyridyl nitrogen (*H_a_* in Figure 2) is indicative of the coordination environment of the Au centre and was used to monitor the evolution of the system. In detail, as expected, the intensity of the *H_a_* signal of **1** decreases over 24 h, whilst the product **P** (in purple in Figure 2A, B) is formed only in traces, indicating that the C−C coupling does not proceed to full completion under these conditions. Of note, three new Au(III) species appear, which display a *H*_a_ signal in the typical range of cyclometalated $C^{\wedge }N$ complexes and were thus tentatively assigned to the three possible transmetalation products: the *cis*/*trans* form of the mono‐substituted alkynyl complex [Au(C^CH2^N)(CCPh)Cl] **R1_kin_/R1** and the bis‐substituted alkynyl complex [Au(C^CH2^N)(CCPh)_2_] **R2**, respectively (Scheme 2). Two species appeared directly after mixing (red and blue traces in Figure 2A, B) and in both cases the proton *ortho* to the pyridyl N (9.54 and 9.48 ppm, respectively) is more deshielded than in **1**. The chemical shift of the third species (green traces in Figure 2A, B), also fast forming after mixing in acetone‐*d_6_*, is instead close to the *H_a_* signal of **1** (9.22 ppm) suggesting a similar chemical environment, such as preserving the chloride bound in *trans* to C8. Of note, the species corresponding to the red traces is short‐lived and quickly disappears, while the other two increase over time (Figure 2A, B). ^1^H NMR monitoring was also carried out in different deuterated solvents (DMSO‐*d_6_*, MeOH‐*d_4_*, DMF‐*d_7_* and MeCN‐*d_3_* in Figures S9–S12, Supporting Information) at room temperature. Overall, all reactions followed a similar path as in acetone‐*d_6_* and the same intermediates were detected.

As previously mentioned for the C−P cross‐coupling reaction starting from **1**, we proposed a general reaction mechanism whereby a phosphane ligand can first replace the chloride *trans* to the N of the $C^{\wedge }N$ ligand to give the thermodynamically favoured [Au($C^{\wedge }N$ )Cl(phosphane)]^+^ species, followed by a second chloride substitution reaction to achieve [Au($C^{\wedge }N$ )‐(phosphane)_2_]^2+^.^[12]^ Both species can independently evolve to C−P coupling through reductive elimination. Based on these results, the C_aryl_−C_alkyne_ coupling under investigation is hypothesised to proceed as depicted in Scheme 2. Specifically, the first chloride to alkynyl transmetalation occurs *trans* to the N of the $C^{\wedge }N$ ligand to give the thermodynamically favoured [Au(C^CH2^N)(CCPh)Cl] complex **R1**. A second transmetalation step would then afford the bis‐alkynyl complex **R2**. Both **R1** and **R2** can independently evolve through reductive elimination to give the cross‐coupling product **P**. The third possible Au(III) species, the mono‐alkynyl complex **R1_kin_** resulting from transmetalation in *cis* position to N, is anticipated to form quickly (due to the stronger *trans* influence of C versus N), but to be thermodynamically less stable than **R1**. Therefore, it may be associated with the red *H*_a_ NMR‐signal that rapidly disappears over time (Figure 2A, B).

**Figure 3:**
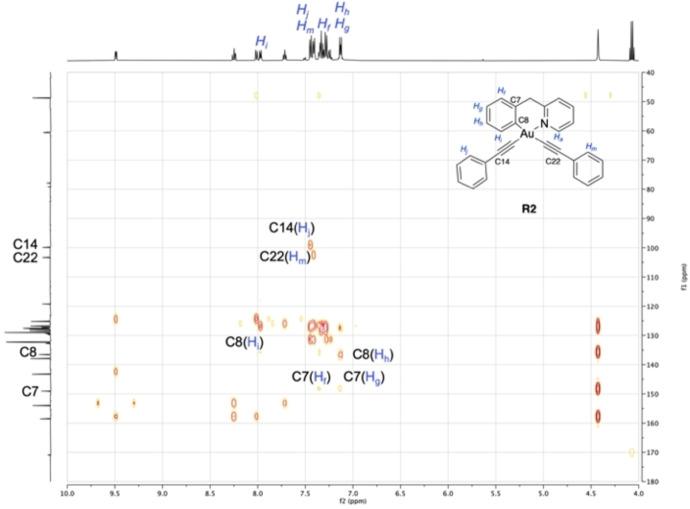
^1^H‐^13^C HMBC NMR spectrum of **R2** in acetone‐*d_6_* highlighting the coupling of C7 with *H_f_* and *H_g_*, C8 with *H_i_* and *H_h_*, C14 with *H_j_* and C22 with *H_m_*.

To support our mechanistic hypothesis, we optimised the reaction conditions in an attempt to isolate the various observed species before C−C coupling could occur. Initially, compound **1** (0.11 mmol) was reacted with 3 equiv. of AgPhCC at room temperature in acetone. The gold complex obtained under these conditions was isolated in 48 % yield after purification by column chromatography. The NMR and HR‐ESI‐MS data (Figures S1, S13‐S18, Supporting Information) indicate $C^{\wedge }N$ ‐cyclometalation to Au(III) (∂_*Ha*_=9.48 ppm) and the presence of two inequivalent PhCC moieties likely to correspond to bis‐alkynyl species **R2** (Scheme 2). This hypothesis was unambiguously confirmed by 2D HMBC and HSQC experiments enabling the assignment of all of the ^1^H and ^13^C resonance signals. Most diagnostic in the HMBC NMR spectrum (Figure 3) are the two C(sp)‐Au resonance signals (C13 and C21) at 77.85 and 79.16 ppm, while the corresponding C(sp)‐Ph signals (C14 and C22) are observed at 99.91 and 103.29 ppm. Notably, the ^1^H NMR spectrum of **R2** corresponds to the “blue” NMR signals in the reaction performed in acetone‐*d_6_* at room temperature (Figure 2A, B). Noteworthy, **R2** formation was also detected by HR‐ESI‐MS within the crude mixture obtained by reacting **1** with AgPhCC at 50 °C leading to **P** (Scheme 1, Figure S19, Supporting Information).

To further demonstrate the involvement of **R2** in the formation of the cross‐coupling product **P**, the isolated compound **R2** was treated with 2 equiv. of AgPhCC in acetone (25 mL) at 50 °C for 48 h. This reaction led again to **P**, which could be isolated in 80 % yield after purification by column chromatography. Formation of product **P** could also be observed when **R2** was heated to 50 °C in acetone over a short period of time, without addition of AgPhCC (data not shown). Overall, our findings further support the hypothesis that **R2** is an intermediate species in the reaction pathway to achieve **P**, in line with previous observations on the C−C and C−P cross‐coupling mechanisms of this family of N^C‐cyclometalated Au(III) complexes.[^12^, ^24^]

In order to synthesize and characterize the hypothesised mono‐substituted alkynyl derivative [Au(C^CH2^N)Cl(CCPh)] (**R1**, Scheme 2), we carried out a redistribution reaction from the isolated bis‐alkynyl complex. For chloride/aryl exchange in P^C‐cyclo‐metalated Au(III) complexes, see ref. [10]. Thus, **R2** was mixed with an equimolar amount of **1** in acetone at room temperature overnight, resulting in the isolation of the mono‐PhCC‐substituted complex **R1** with a yield of 85 % after purification. The structure of the complex was confidently assigned thanks to careful identification of all the ^1^H and ^13^C NMR signals, also based on HSQC and HMBC NMR and MS experiments (Figure S1, S20–S26, Supporting Information). Most remarkably, the *H_a_* chemical shift of the isolated species **R1** was located at 9.22 ppm in acetone‐*d_6_*, thus, matching with the signals of the “green” species in Figure 2 and Figures S9–S12 (Supporting Information). Given that **R1** is obtained by redistribution of chlorido/alkynyl ligands from **R2** and **1**, it is likely to correspond to the most thermodynamically stable substitution isomer, with the alkynyl group in *trans* position to the pyridyl N (Scheme 2). The difference in stability between the two isomers **R1** and **R1_kin_** was assessed computationally by DFT and, as expected, **R1** was found to be significantly lower in energy than **R1_kin_**, by 59.4 kJ mol^−1^ (Figure S27, Supporting Information). The redistribution reaction of **1** and **R2** into **R1** was calculated according to Equation (1) below and was found to be downhill in energy by 31 kJ mol^−1^ per Au centre. The fact that the chemical shifts of *H_a_* in **R1** (9.22 ppm) and **1** (9.30 ppm) are very close supports this assignment.

(1) $$\Delta G{}^{\circ }=\left\{2\times G{}^{\circ }\left[R1\right]-G{}^{\circ }\left[R2\right]-G{}^{\circ }\left[1\right]\right\}/2$$

**Figure 4:**
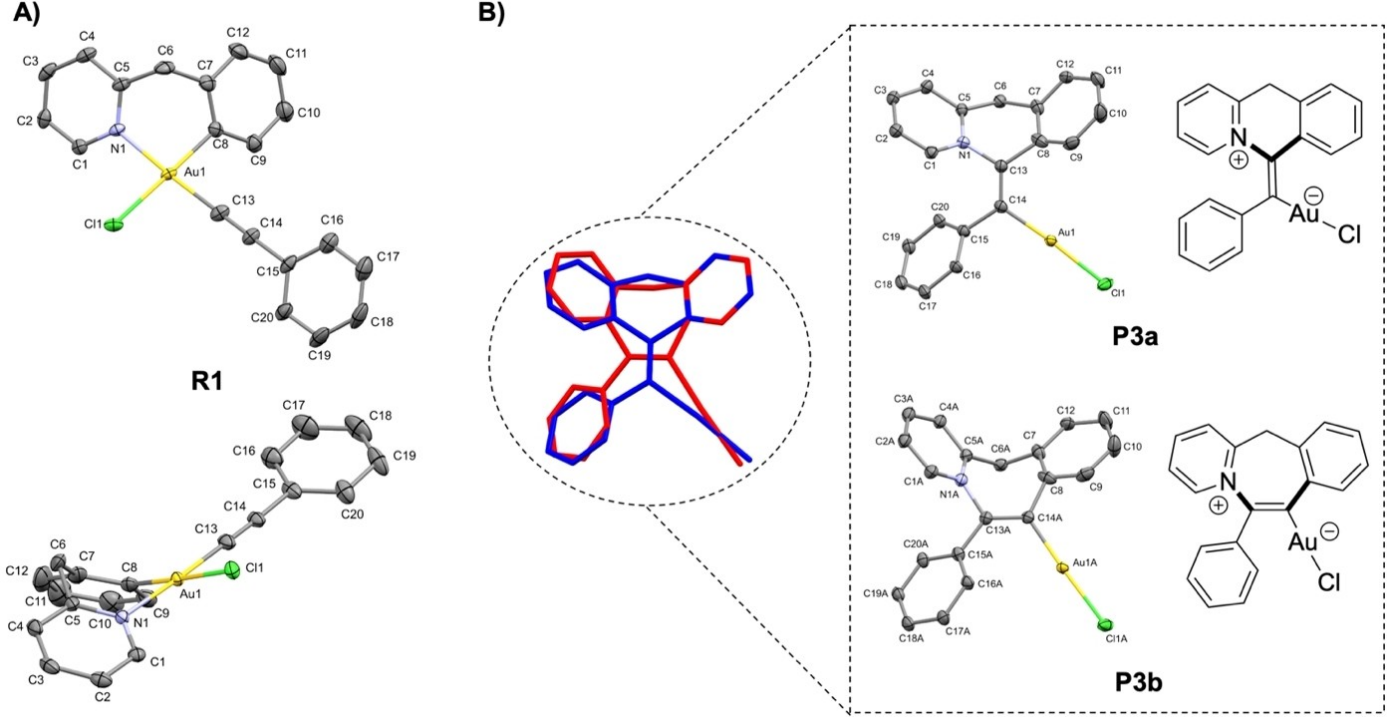
**A**) Representations of the molecular structure of **R1** in the solid state (second polymorph) with ellipsoids at the 50 % probability level, H atoms are omitted for clarity. Selected distances [Å] and angles [°]: Au1‐Cl2 2.3690(6), Au1‐N1 2.074(2), Au1‐C8 2.017(2), Au1‐C13 1.965(2), Cl1‐Au1‐N1 91.01(5), N1‐Au1‐C8 88.85(8), C8‐Au1‐C13 88.5(1) C13‐Au1‐Cl1 91.66(7), C8‐Au1‐Cl1 178.33(7), N1‐Au1‐C13 177.10(9). **B**) Molecular structures of insertion‐products **P3a** and **P3b** with corresponding ChemDraw structures shown on the right, with the overlap view of them as co‐crystallised in the solid state. Ellipsoids are shown at 50 % probability and H atoms are omitted for clarity.

To further confirm the identity of **R1** and the position of the alkynyl group at gold, crystals were grown by layering *n*‐pentane over a diluted chloroform solution of **R1**. This produced a milky interface from which crystals suitable for X‐ray diffraction analysis were formed over 24 h. Single‐crystal X‐ray diffraction confirmed the structure of **R1** as shown in Figure 4A. Two polymorphs of **R1** were observed, both crystallising in the monoclinic space group *P*2_1_/c/*P*2_1_/n (No. 14). On the molecular level, the difference between them is the disorder of the alkynyl ligand over three different orientations of the phenyl ring in the first polymorph (see Supporting Information, XRD data), whereas this ligand is fully ordered in the second polymorph. All structures are very similar in geometry, so for sake of clarity, only the latter is discussed here. Accordingly, **R1** features the typical square planar coordination geometry of Au(III) complexes with the alkynyl ligand *trans* to the N binding site and the chloride *trans* to the C binding site of the C^CH2^N ligand. The distortion from ideal square planar geometry is very small as shown by the τ_δ_ value of 0.03 (according to the concept of Houser and Kubiak, 0 corresponds to ideal square planar and 1 to ideal tetrahedral coordination).^[25]^ The bond distances Au1−Cl2=2.3690(6) Å, Au1−N1=2.074(2) Å and Au1−C8=2.017(2) Å are similar to those found in analogous cationic literature‐reported complexes bearing a PTA or triphenylphosphine instead of the alkynyl ligand.^[26]^ The bite angle of the bidentate C^CH2^N ligand N1−Au1−C8=88.85(8)° is slightly wider than in the literature complexes. The six‐membered metallacycle of the C^CH2^N ligand coordinated to Au(III) features a boat‐like conformation (Figure 4A, bottom representation) in which the (N1 Au1 C8) plane and (C5 C6 C7) plane form angles of 37.29° and 47.14°, respectively, with the mean (N1 C5 C7 C8) plane, which is in a similar range as in the mentioned literature‐known complexes.^[26]^

Interestingly, from the same crystallisation experiments, single crystals of the zwitterionic Au(I) vinyl products **P3a** and **P3b** were also obtained, both being constitutional isomers of **R1** which co‐crystallised as a disordered mixture of both molecules (with a ratio of ca. 62 : 38) on the same site (see Figure 4B). Due to this disorder, no detailed bond lengths and angles will be discussed here. The formation of **P3a**/**P3b** involves C(sp^2^)−C(sp) coupling, as observed in **P**, as well as C−N coupling. A somewhat related difunctionalization of CC bonds with C−C and C−N bond formation catalysed by gold has been previously reported. It proceeds by a completely different route, i.e. visible light‐mediated addition of a diazonium salt to gold, migratory insertion of the alkyne followed by reductive elimination, see ref. [43]. The C and N atoms of the C^CH2^N ligand are connected through either the terminal carbon atom (**P3a**) or the two carbon atoms (**P3b**) of the former C≡C bond, resulting in 6 or 7‐membered rings, respectively. In both complexes, the N atom and gold centre are in *trans* position with respect to the C13=C14 double bond, in line with *anti* nucleophilic addition of the pyridine to the alkyne π ‐activated at gold (see below for further mechanistic discussions). The zwitterionic Au(I) vinyl complexes **P3a**/**P3b** were not detected in the NMR studies, but their crystallographic authentication highlights a possible follow‐up process for the C(sp^2^)−C(sp) coupling between **1** and AgPhCC.

Regarding the last “red” species observed in our ^1^H NMR monitoring (red traces in Figure 2, ∂_*Ha*_=9.54 ppm in acetone‐*d_6_*), any attempt in isolating it was unsuccessful. Nonetheless, having unambiguously identified **R1** and **R2**, it was deduced to be **R1_kin_**, the other isomer of the mono‐alkynyl complex [Au(C^CH2^N)Cl(CCPh)] with the CCPh group *cis* to N. This is consistent with the fact that this species is observed at short reaction times but then converts into **R1** and **R2**. From a spectroscopic perspective, it is noteworthy that the corresponding *H_a_* NMR resonance signal (9.54 ppm) is somewhat deshielded compared to those of **1** (9.30 ppm) and **R1** (9.22 ppm), but similar to that observed for **R2** (9.48 ppm). This probably reflects the chemical environment of *H_a_* with an alkynyl group sitting in *cis* position in both **R1_kin_** and **R2**.

Overall, the analysis of the *H_a_* intensity trends in Figure 2 and Figures S9–12 (Supporting Information) clearly shows that consumption of **1** results in the formation of **R1_kin_**, **R1** and **R2**. Moreover, the authentication of products **P3a** and **P3b** reveals that depending on the reaction conditions, the release of C(sp^2^)−C(sp) coupling product **P** may be ‘parasitised’ by C−N coupling (promoted by π ‐activation at gold).

### DFT mechanistic studies

The C−C cross coupling reaction mechanism in acetone was also investigated by DFT calculations. For C−S and C−P couplings from cyclometalated Au(III) $C^{\wedge }N$ complexes, pathways involving pyridine decoordination (to enable rotation of the phenyl ring) were previously observed to be favoured.[^7a^, ^12^] Given that an excess of AgPhCC was experimentally found to favour the C(sp^2^)−C(sp) coupling and increase the yield in compound **P**, the formation of Au(III) ate‐complexes by addition of an additional alkynyl moiety to **R2** and **R1** (with displacement of the pyridine) was also considered and investigated.

**Scheme 3:**
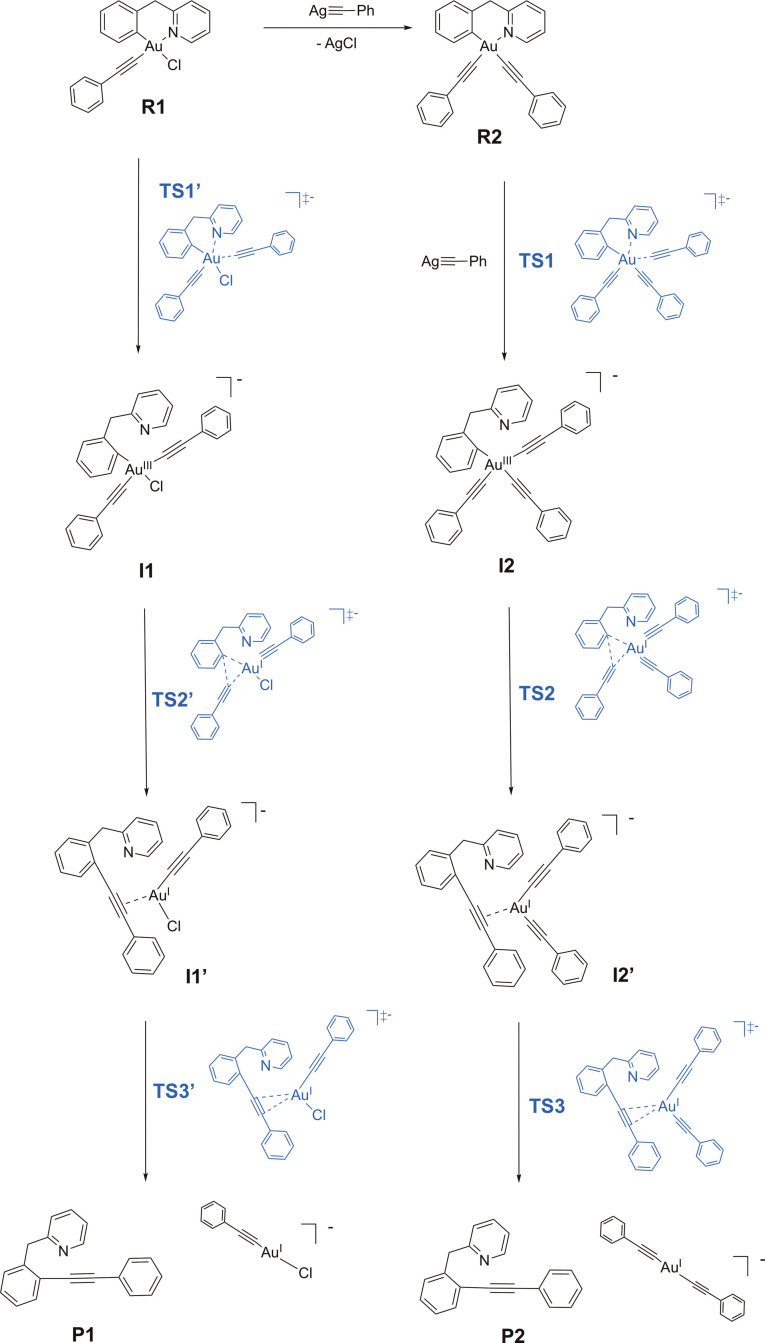
Possible reaction pathways leading to the reductive elimination products calculated by DFT, including transition states.

**Figure 5:**
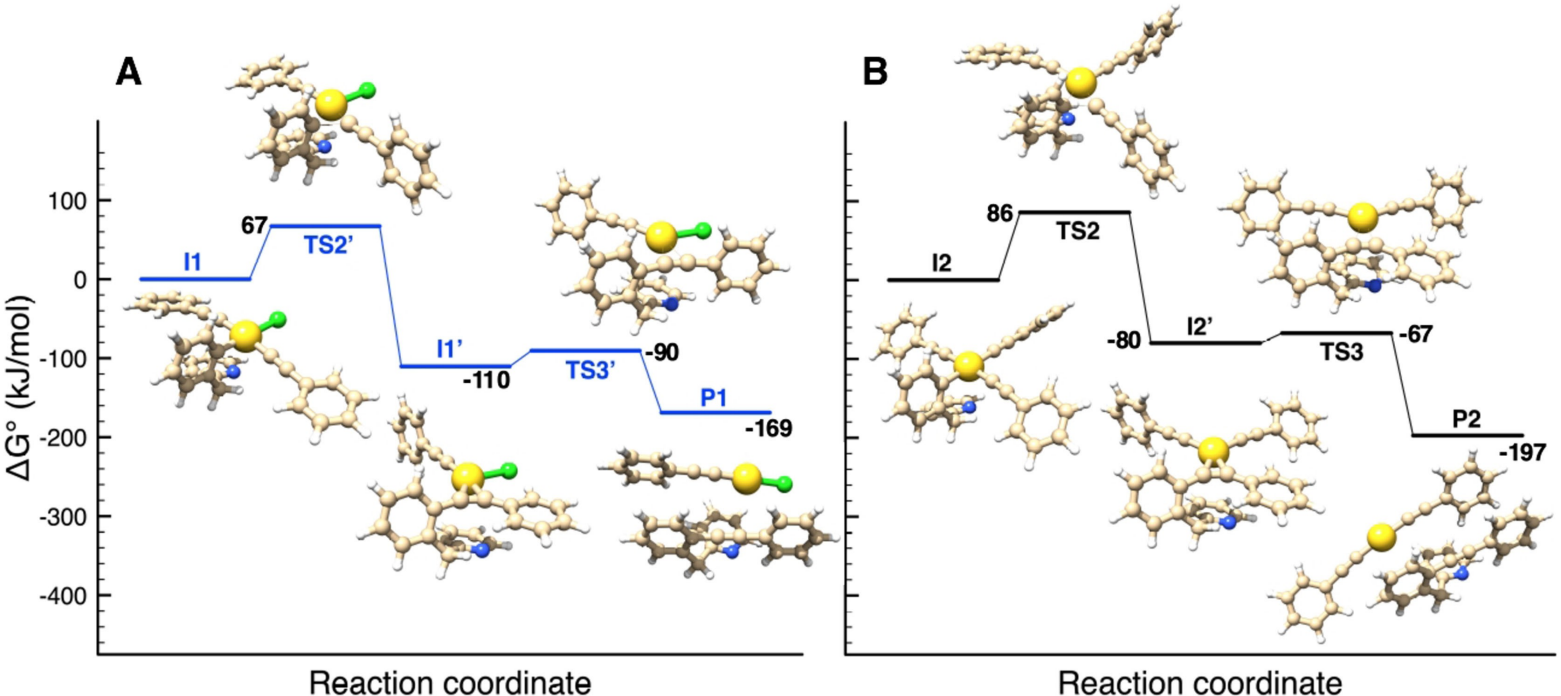
Energy profiles computed for the C(sp^2^)−C(sp) coupling from Au(III) ate‐complexes **I1** (blue line, panel A) and **I2** (black line, panel B). Structures and energies have been obtained by DFT calculations.

Following Schemes 2 and 3, the reaction profiles from **1** to **R1**, from **R1** to **R2**, from **R1** to **I1** and from **R2** to **I2** have been mimicked by using the PhCC^−^ anion as a simplified model reactant, instead of AgPhCC. As shown in Figure S28 (Supporting Information) and Table 2, the four reaction steps are all highly favoured, involving a stabilization of more than 100 kJ mol^−1^ and activation energies of up to 15 kJ mol^−1^. The intramolecular reaction steps going from intermediates **I1** and **I2**, in which the AuPhCC binding induced Au(III)‐N‐pyridyl dissociation, to the cross‐coupling products **P1** (**P**+[Au^I^Cl(PhCC)]^−^) and **P2** (**P**+[Au^I^(PhCC)_2_]^−^) are shown in Figure 5, while the corresponding energy values are reported in Table 2. In both cases, reductive elimination is highly exergonic (by −125.1 kJ mol^−1^ for **I2** (from **R2**) and −116.6 kJ mol^−1^ for **I1** (from **R1**)). The associated activation barrier from **I2** (85.8 kJ mol^−1^) falls in the same range as those found from **R1** and **R2**, while it is slightly lower from **I1** (67.1 kJ mol^−1^).

**Table 2:** Calculated relative standard Gibbs free energy values (ΔG°) and activation barriers (E^≠^, kJ mol^−1^) of the species involved in the proposed C−C cross‐coupling reaction pathways of compound **1**, in acetone solution, leading to the **P1**, **P2**, **P3a** and **P3b** products and to putative intermediate species **I4’**.

Species	ΔG°	Species	ΔG°
1+PhCC^−^	0.0	R1+PhCC^−^	0.0
TS0=E_0_^≠^	5.3	TS00=E_00_^≠^	13.8
R1+Cl^−^	−162.8	R2+Cl^−^	−104.3
R1+PhCC^−^	0.0	R2+PhCC^−^	0.0
TS1’=E_1_^≠’^	15.4	TS1=E_1_^≠^	7.3
I1	−116.6	I2	−125.1
I1	0.0	I2	0.0
TS2’=E_2_^≠’^	67.1	TS2=E_2_^≠^	85.8
I1’	−110.4	I2’	−79.9
TS3’	−90.5	TS3	−67.3
E_3_^≠’^	19.9	E_3_^≠^	12.6
P1	−168.9	P2	−197.1
R1	0.0	R2	0.0
TS4’=E_4_^≠’^	87.9	TS4=E_4_^≠^	80.7
I3	−115.6	I4	−126.3
TS5’	−88.7	TS5	−122.1
E_5_^≠’^	26.9	E_5_^≠^	4.3
I3’	−101.9	I4’	−139.9
TS6a	−62.8		
E_6_^≠^	39.1		
P3a	−145.0		
TS6b	−60.0		
E_7_^≠^	41.9		
P3b	−142.9

In detail, each of the two reaction pathways involve two transition states (**TS2**, **TS2’** and **TS3**, **TS3’**, respectively) and one π‐complex intermediate (**I1’** and **I2’**) in which the C−C bond is formed between the phenyl and phenylacetylide groups while the C≡C triple bond is π ‐coordinated to gold. The alkyne moiety sits in the Au(CCPh)_2_/Au(CCPh)Cl coordination plane, to maximize Au→π *(CC) backdonation. Dissociation of the alkyne to release the coupling product **P** along with the corresponding anionic dicoordinate Au(I) complexes is downhill in energy (Δ G°=−58.5(**I1’**)/−117.2(**I2’**) kJ mol^−1^) and proceeds easily (the activation barrier Δ G^≠^ being of 19.9 (to **I1’**) and 12.6 (to **I2’**) kJ mol^−1^, respectively.

The comparison of the energy trends of the two reaction pathways leading to **P1** and **P2** (Figure S29, Supporting Information) shows that the reaction of the monosubstituted intermediate **I1** leading to **P1** is kinetically favoured, being the first transition state **TS2’** of ca. 19 kJ mol^−1^ lower in energy than **TS2** (67.1 vs. 85.8 kJ mol^−1^, see also Table 2). On the other hand, **P2** is thermodynamically favoured with respect to **P1**, of ca. 28 kJ mol^−1^, due to the larger stability of the co‐product [Au^I^(PhCC)_2_]^−^ compared to [Au^I^Cl(PhCC)]^−^.

It is also interesting to compare the energy trend of the C−C versus the C−P cross‐coupling reaction, obtained by DFT calculations at the same level of theory.^[12]^ Since the C−P reaction was computationally investigated considering the (C^CO^N) ligand, we have here reported for comparison the results of the similar reaction pathway involving compound **1** (Figure S30, Supporting Information). This figure shows that the activation barriers of the determining steps are all comparable (ca. 86 vs. 83 kJ mol^−1^ for C−C and C−P reaction pathways, respectively).

**Figure 6:**
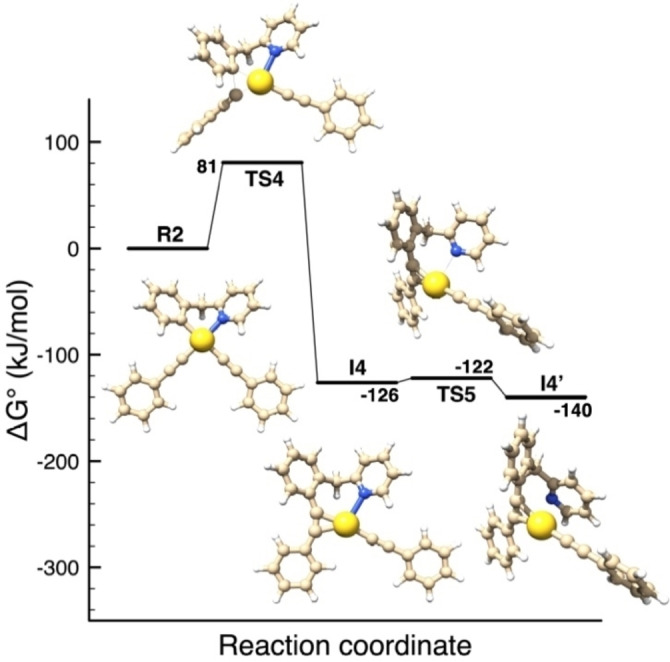
Energy profile computed for the C(sp^2^)−C(sp) coupling at Au(III) from the experimentally observed bis‐alkynyl complex **R2**. Structures and energies have been obtained by DFT calculations.

Considering that reductive elimination was experimentally observed also starting from the isolated bis‐alkynyl complex **R2** upon heating, DFT calculations were performed to evaluate the reaction pathway from **R2** without adding another PhCC^−^ ligand. The results are depicted in Figure 6 and show that an activation energy of at least 80.7 kJ mol^−1^ (E_4_^≠^, Table 2) is required for the C(sp^2^)−C(sp) coupling to occur, in line with the experimental observations.

**Figure 7:**
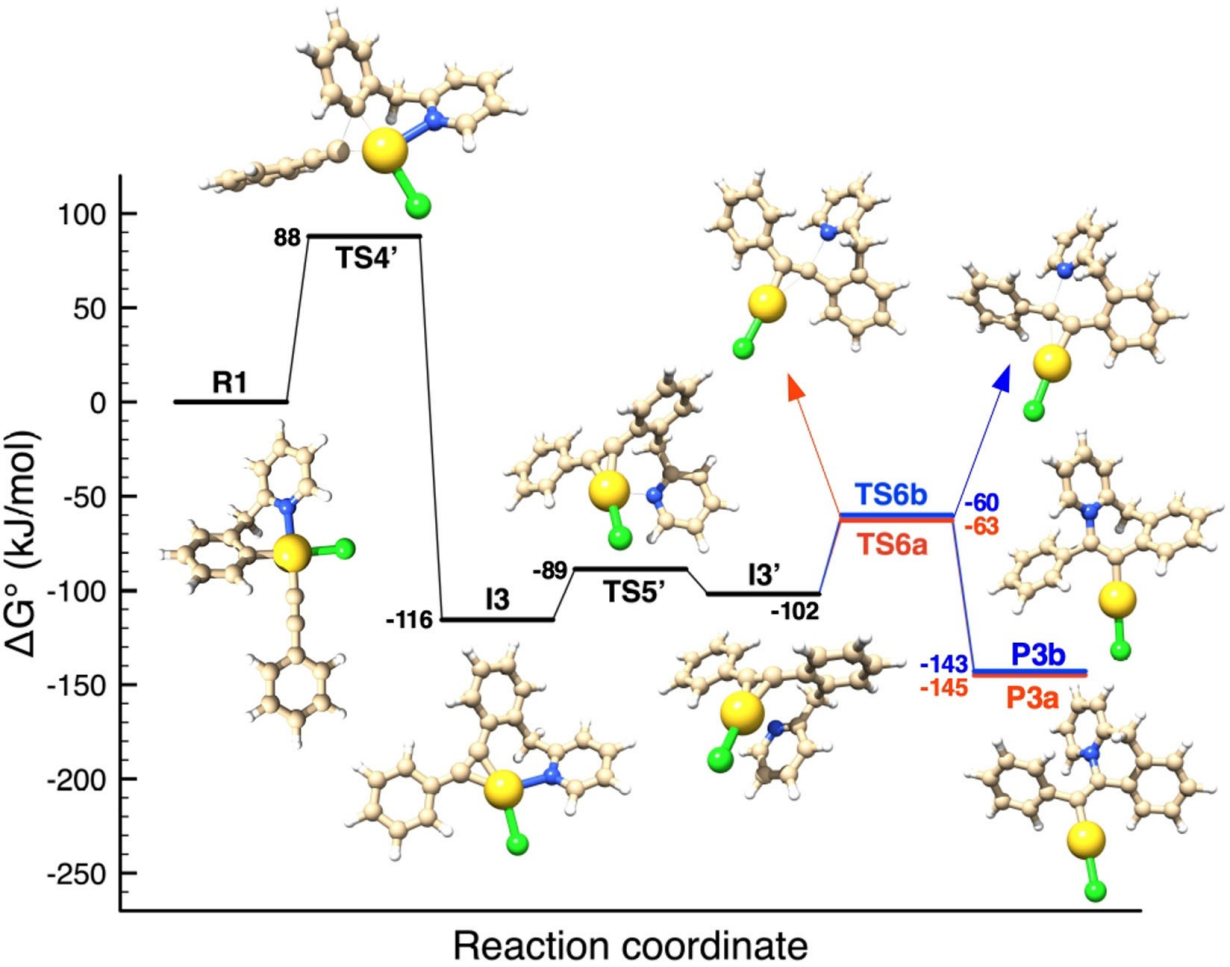
Energy profile computed for the C(sp^2^)−C(sp) coupling at Au(III) from the experimentally observed mono‐alkynyl complex **R1**, as well as for the formation of the zwitterionic Au(I) vinyl complexes **P3a**/**P3b** from **R1**. Structures and energies have been obtained by DFT calculations.

In addition, DFT calculations were exploited to provide an alternative reaction pathway for the formation of the zwitterionic Au(I) vinyl complexes **P3a** and **P3b** from **R1** (Figure 7). From the linear π ‐complex **I3’**, the mechanism involves outer‐sphere nucleophilic attack of the N atom to either of the C atoms of the C≡C bond coordinated to gold. In line with the characterisation of both **P3a** and **P3b**, the two processes are very similar in activation barrier (Δ G^≠^=39.1 and 41.9 kJ mol^−1^) and thermodynamics (Δ G°=−43.1 and −41.0 kJ mol^−1^). The activation barrier of the first step of this ‘parasitic’ pathway (**E_4_** ^**≠**^’, Table 2) is in the upper range of those calculated for the reaction pathways shown in Figure 5. Of note, the reaction step from **R1** to **I4’** can be considered as essentially irreversible, since the activation barrier from **I3’** to **TS4’** is more than 200 kJ mol^−1^.

### C(sp^2^)−C(sp^2^) cross‐coupling

**Scheme 4:**
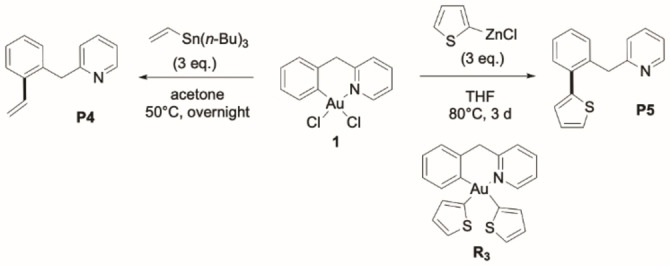
C(sp^2^)−C(sp^2^) cross‐couplings of the cyclometalated Au(III) C^CH2^N complex **1** with vinyltin and heteroarylzinc reagents.

The ease of C(sp^2^)−C(sp) cross‐coupling, as observed with AgPhCC, prompted us to explore the possibility to extend the transformation to other C‐based nucleophiles. Alkynyl substrates are somewhat peculiar. Because of the C(sp) hybridization, the reacting C atom is very sterically unshielded and the ≡C−H bond is easily activated. We thus wondered about the possibility to generalise the reaction to C(sp^2^)‐based nucleophiles and C(sp^2^)−C(sp^2^) cross‐couplings that are extremely rare from $C^{\wedge }N$ ‐cyclometalated Au(III) complexes. To the best of our knowledge, there is in fact only one precedent from the You group under oxidative catalytic conditions with arylboronic acids as partners.^[13f]^ With the aim to achieve C−C cross‐coupling with mild vinylic and aromatic organometallic reagents, we turned to tin and zinc derivatives. Vinyl and 2‐thienyl groups were chosen to facilitate reaction monitoring and product authentication by NMR spectroscopy. Accordingly, the cyclometalated Au(III) $C^{\wedge }N$ complex **1** was found to smoothly react with vinyl‐tri(*n*‐butyl)stannane (3 equiv.) in acetone to give the corresponding vinylation product **P4** (quantitative spectroscopic yield) after heating at 50 °C overnight (Scheme 4, Figures S31–35, Supporting Information). The reaction works also with heteroaryl nucleophiles, as illustrated by the use of (2‐thienyl)zinc chloride (reaction complete within 3 days at 80 °C in THF, 71 % isolated yield). The ensuing thiofurane‐pyridine product **P5** was unambiguously authenticated by NMR spectroscopy and mass spectrometry (see Figures S41–S45, Supporting Information). Of note, the bis(2‐thienyl) Au(III) intermediate **R3** related to the bis‐alkynyl complex **R2** could be prepared at RT and fully characterized, including by X‐ray diffraction analysis (see Figures S36–S40, Supporting Information).

## Conclusion

Cross‐coupling strategies have become essential in modern organic synthesis. While palladium has historically dominated this field, other transition metals have arisen as efficient and orthogonal alternatives. Gold has been typically exploited in catalysis mostly for its Lewis acidity,^[27]^ however, a number of studies have demonstrated the involvement of its redox chemistry in several cross‐coupling reactions using external oxidants, photoredox catalytic cycles or chelating/hemilabile ligands.[^2c^, ^2d^, ^4f^, ^28^] Further developments in this still young area of gold‐promoted cross‐coupling reactions have been hampered by the scarce mechanistic understanding of the individual steps along the proposed catalytic cycles. The elucidation of these mechanisms has been a great challenge due to the reactivity of labile and rapidly evolving high‐valent gold intermediates, which often prevents their isolation.

In this context, our study explores the feasibility of C−C cross‐coupling templated by a cyclometallated Au(III) C^CH2^N complex, endowed with sufficient stability to provide insights into the mechanism of reductive elimination from Au(III). The reaction of complex **1** with AgPhCC to achieve C(sp^2^)−C(sp) coupling was thoroughly investigated. By tuning the reaction conditions and monitoring its progress carefully by NMR, a number of intermediate species involved in the reductive elimination process could be isolated and characterized by different spectroscopic and analytical methods, including XRD. Moreover, some ‘parasitic’ Au(I) species have been identified during crystallization attempts: namely, the zwitterionic cyclic Au(I) vinyl complexes **P3a** and **P3b**, whose formation cannot be reversed towards the reductive elimination intermediates leading to the desired C−C product. In addition, we have demonstrated the feasibility of C(sp^2^)−C(sp^2^) cross‐couplings by complex **1** with vinyl and heteroaryl tin and zinc reagents under mild conditions.

DFT studies provide further insight into the mechanism of the C−C cross‐coupling reaction and formation of the zwitterionic Au(I) side complexes. Of note, reductive elimination at Au(III) affords a π‐complex in which the alkynyl moiety of the coupling product is side‐on coordinated to Au(I). This opens the way to intramolecular nucleophilic attack by the pendant pyridine moiety, leading to the zwitterionic Au(I) vinyl complexes **P3a** and **P3b**.

Better understanding of the course and fate of reactions between organometallic species and Au(III) complexes will help to favour the desired paths and ultimately to improve efficiency and expand the scope of gold‐promoted C−C coupling reactions. Further studies are ongoing in our laboratory on derivatives of **1** featuring modifications of the $C^{\wedge }N$ scaffold to identify possible structure‐activity relationships, as previously achieved for C−P and C−S bond forming reactions.

## Experimental Section

**General**: Solvents and reagents (reagent grade) were all commercially available and used without further purification. ^1^H, ^13^C and 2D NMR spectra were recorded in Acetone‐*d_6_* solution, with TMS as an internal reference, on Bruker Avance (300–500 MHz) NMR spectrometers and analysed using MestReNova v.14 by Mestrelab Research S.L. Benzylether was used as an internal standard to quantify the yield of the reaction in DMSO*‐d_6_*. Hexamethylbenzene was used as an internal standard to quantify the yield of the coupling reactions with stannanes performed in acetone*‐d_6_*. HR‐ESI‐MS spectra were recorded on Synapt G2‐Si time‐of‐flight (TOF) mass spectrometer (Waters). Mass spectra were acquired and processed using MassLynx V4.1 (Waters). Complex **1** and AgPhCC were both synthesized following literature procedures.^[29]^ The purity of **R1** was confirmed by elemental analysis.

### Synthesis of the compounds

**Synthesis of product P**: [Au(C^CH2^N)Cl_2_] (**1**) (1 equiv., 50 mg, 0.11 mmol) was suspended in 25 mL of acetone prior to the addition of silver phenylacetylide (3 equiv., 71 mg, 0.34 mmol). The mixture was stirred at 50 °C overnight. After filtration of the resulting suspension, purification via flash chromatography (*n*‐hexane:EtOAc 85 : 15) afforded the desired product as a yellow oil (0.037 mmol, 10 mg, 33 %). ^1^H NMR (400 MHz, Acetone‐*d_6_*) δ 8.52 (d, *J*=5.8 Hz, 1H), 7.66 (td, *J*=7.7, 1.9 Hz, 1H), 7.59–7.50 (m, 3H), 7.47–7.37 (m, 3H), 7.39–7.27 (m, 3H), 7.25 (d, *J*=7.9 Hz, 1H), 7.18 (ddd, *J*=7.6, 4.8, 1.2 Hz, 1H), 4.43 (s, 2H). ^13^C NMR (126 MHz, Acetone‐*d_6_*) δ 160.42, 149.23, 141.75, 136.26, 132.05, 131.37, 130.15, 128.68, 128.54, 128.48, 126.54, 123.22, 122.95, 122.85, 121.23, 93.25, 88.08, 42.76. HR‐ESI‐MS (CH_3_CN, pos. mode) for C_20_H_16_N^+^: exp. 270.1307 (calc. 270.1283).

**Synthesis of R2**: Compound **1** (1 equiv., 48 mg, 0.11 mmol) was suspended in 14 mL of acetone prior to the addition of silver phenylacetylide (3 equiv., 71 mg, 0.34 mmol). The mixture was stirred at room temperature overnight. After filtration of the resulting suspension, purification via flash chromatography (DCM:Methanol 100–>98 %) afforded the desired product as a yellow solid (0.055 mmol, 31 mg, 48 %). ^1^H NMR (500 MHz, Acetone‐*d_6_*) δ 9.48 (d, *J*=5.0 Hz, 1H), 8.24 (td, *J*=7.7, 1.6 Hz, 1H), 8.01–7.99 (m, 1H), 7.99–7.96 (m, 1H), 7.70 (ddd, *J*=7.4, 5.8, 1.5 Hz, 1H), 7.46–7.40 (m, 4H), 7.36–7.22 (m, 7H), 7.15–7.11 (m, 2H), 4.41 (s, 2H). ^13^C NMR (126 MHz, Acetone‐*d_6_*) δ 158.55, 154.01, 149.04, 143.27, 137.97, 136.53, 132.28, 132.22, 129.04, 128.96, 128.43, 127.85, 127.64, 127.51, 127.40, 127.22, 126.79, 125.22, 119.20, 103.29, 99.91, 79.16, 77.85, 48.75. HR‐ESI‐MS (CH_3_CN, pos. mode) for C_28_H_20_NNa^+^: exp. 590.1146 (calc. 590.1159).

**Synthesis of R1**: Compound **R2** (1 equiv., 61.7 mg, 0.11 mmol) was dissolved in 16 mL of acetone prior to the addition of an equimolar amount of **1** (1 equiv., 48 mg, 0.11 mmol). The resulting suspension was stirred overnight turning into a pale‐yellow solution. Purification via flash chromatography (*n*‐hexane:EtOAc 40 : 60) afforded the desired product as an off white solid (0.1 mmol, 51 mg, 85 %). ^1^H NMR (400 MHz, Acetone‐*d_6_*) δ 9.22 (dd, *J*=5.8, 1.6 Hz, 1H), 8.24 (td, *J*=7.7, 1.6 Hz, 1H), 7.98 (d, *J*=7.5 Hz, 1H), 7.80 (dd, *J*=7.7, 1.3 Hz, 1H), 7.72 (ddd, *J*=7.5, 5.8, 1.5 Hz, 1H), 7.43–7.36 (m, 2H), 7.40–7.26 (m, 5H), 7.20 (t, *J*=7.3 Hz, 1H), 7.11 (t, *J*=7.6 Hz, 1H), 4.47 (s, 1H). ^13^C NMR (126 MHz, Acetone‐*d_6_*) δ 157.40, 152.19, 143.31, 139.95, 136.54, 134.87, 132.30, 129.58, 129.05, 128.61, 128.10, 128.02, 127.92, 126.65, 126.59, 125.37, 125.09, 98.94, 81.67, 47.52. HR‐ESI‐MS (CH_3_CN, pos. mode) for C_20_H_15_AuN^+^: exp. 466.0833 (calc. 466.0870).

Elemental analysis for C_41_H_44_Au_2_Cl_4_N_2_O_6_ (2**R1**‐6H_2_O ⋅ CH_2_Cl_2_): exp. C 41.04 %, H 3.43 %, N 2.87 % (calc. C 41.16 %, H 3.71 %, N 2.34 %).

**Synthesis of P4**: On preparative scale, complex **1** (1 equiv., 43 mg, 0.10 mmol) was suspended in 10 mL of acetone prior to the addition of vinyl‐tri(*n*‐butyl)stannane (3 equiv., 88 μL, 0.30 mmol). The mixture was stirred at 50 °C overnight. Purification via flash chromatography (*n*‐pentane:EtOAc 85 : 15) afforded the product as a mixture with (*n*Bu)_3_SnCl. Then the mixture was dissolved in acetonitrile (10 mL) and (*n*Bu)_3_SnCl was extracted with pentane (3×10 mL). Acetonitrile was removed under vacuum to afford the desired product as an oil (0.044 mmol, 8.5 mg, 44 %). A reaction was performed in an NMR tube (starting from 0.01 mmol of **1**) with hexamethylbenzene as internal standard. The NMR yield was quantitative (>99 %). ^1^H NMR (300 MHz, Acetone‐*d_6_*) δ 8.51–8.47 (m, 1H), 7.64 (td, *J*_HH_=7.7, 1.9 Hz, 1H), 7.60–7.53 (m, 1H), 7.31–7.20 (m, 3H), 7.20–7.15 (m, 1H), 7.14 (dd, J_HH_=17.4, 11.0 Hz, 1H), 7.11–7.07 (m, 1H), 5.65 (dd, *J*_HH_=17.4, 1.5 Hz, 1H), 5.23 (dd, *J*_HH_=11.0, 1.5 Hz, 1H), 4.23 (s, 2H). ^13^C NMR (75 MHz, Acetone‐*d_6_*) δ 161.65, 149.93, 137.92, 137.84, 137.32, 135.84, 131.59, 128.77, 127.74, 126.49, 123.65, 122.06, 115.79, 42.38. EI‐MS for C_14_H_11_N: exp. 195.25 (calc. 195.10).

**Synthesis of (2‐thienyl)zinc chloride**: To a solution of 2‐bromothiophene (194 μL, 2 mmol) in THF (4 mL, 0.5 m) was added *n*BuLi (1.5 mL, 1.46 m, 1.1 equiv.) dropwise at −78 °C and stirred for 1 h. A freshly prepared ZnCl_2_ solution (300 mg, 2 mL of THF, 1.1 equiv.) was added dropwise at −78 °C and then the reaction mixture was allowed to warm to rt and stirred for 1 h. The solution was titrated using a THF solution of I_2_.

**Synthesis of R3**: Compound **1** (1 equiv., 43 mg, 0.10 mmol) was suspended in 9 mL of THF prior to the addition of (2‐thienyl)zinc chloride (3 equiv., 1.1 mL of solution in THF, c=0.26 m, 0.30 mmol) at 0 °C. The mixture was stirred at rt for 2 h. Purification via flash chromatography (*n*‐pentane:EtOAc 70 : 30) afforded **R3** as a white solid (22.1 mg, 42 % yield). Crystals suitable for X‐ray diffraction analysis were obtained by slow evaporation of a DCM solution. ^1^H NMR (500 MHz, CD_2_Cl_2_) δ 8.55–8.51 (m, 1H), 7.89 (td, *J*_HH_=7.7, 1.7 Hz, 1H), 7.66 (d, *J*_HH_=7.7 Hz, 1H), 7.47 (dd, *J*_HH_=4.8, 0.8 Hz, 1H), 7.41 (dd, *J*_HH_=5.1, 1.0 Hz, 1H), 7.28–7.24 (m, 2H), 7.23 (dd, *J*_HH_=7.3, 1.5 Hz, 1H), 7.16 (dd, *J*_HH_=4.8, 3.3 Hz, 1H), 7.08 (dd, J_HH_=5.1, 3.3 Hz, 1H), 7.03 (td, *J*_HH_=7.3, 1.5 Hz, 1H), 6.98 (td, td, *J*_HH_=7.3, 1.6 Hz, 1H), 6.96 (dd, *J*_HH_=3.4, 1.0 Hz, 1H), 6.90 (dd, *J*_HH_=3.3, 0.8 Hz, 1H), 4.38 (s, 2H). ^13^C NMR (126 MHz, CD_2_Cl_2_) δ 163.95, 159.10, 157.72, 152.00, 141.46, 137.27, 136.49, 131.08, 130.72, 128.32, 127.94, 127.72, 127.54, 127.16, 127.10, 126.63, 126.27, 125.79, 124.02, 49.30. EI‐MS for C_16_H_13_AuNS: exp. 448.0 (calc. 448.04).

**Synthesis of P5**: Compound **1** (1 equiv., 88 mg, 0.20 mmol) was suspended in 17 mL of THF prior to the addition of (2‐thienyl)zinc chloride (3 equiv., 2.8 mL of solution in THF, c=0.22 m, 0.60 mmol) at 0 °C. The mixture was stirred at 80 °C for 3 days. The rection mixture was quenched by addition of an aqueous solution of HCl and extracted with DCM. Organic layers were washed with brine and dried over Na_2_SO_4_. After filtration and concentration, the product was purified via flash chromatography (*n*‐pentane:EtOAc 80 : 20) to give the desired product (35.7 mg, 71 % yield). ^1^H NMR (300 MHz, CDCl_3_) δ 8.53 (d, *J*_HH_=4.3 Hz, 1H), 7.51 (td, *J*_HH_=7.8, 1.9 Hz, 1H), 7.46–7.41 (m, 1H), 7.32–7.25 (m, 4H), 7.11–7.04 (m, 1H), 7.02 (dd, *J*_HH_=5.1, 3.5 Hz, 1H), 6.96 (dd, *J*_HH_=3.5, 1.2 Hz, 1H), 6.88 (d, *J*_HH_=7.8 Hz, 1H), 4.30 (s, 2H). ^13^C NMR (75 MHz, CDCl_3_) δ 161.21, 149.39, 142.56, 137.79, 136.48, 134.72, 131.39, 131.02, 128.39, 127.23, 126.86, 126.73, 125.57, 123.23, 121.19, 42.29. DCI/NH_3_‐MS for C_16_H_13_NS: exp. 252.2 (calc. 252.1).

^**1**^ **H NMR Reaction Monitoring**: The reactions yielding the cross‐coupling product **P** were monitored at room temperature over 24 h by ^1^H NMR spectroscopy, on a Bruker Avance spectrometer (400 MHz). The reaction mixtures were prepared by suspending 1 equiv. of [Au(C^CH2^N)Cl_2_] (**1**) and 2 equiv. of silver phenylacetylide in 1 mL of deuterated solvent in an NMR tube, with TMS as an internal reference. Reactions were carried out in Acetone‐*d_6_*, Methanol*‐d_4_*, Acetonitrile‐*d_3_*, DMF‐*d_7_*, or DMSO*‐d*_6_. The collected spectra were analysed by the Reaction Monitoring plugin in MestReNova v.14 (Mestrelab Research S.L.).

**Computational studies**: DFT calculations were performed to propose a reaction pathway for the C−C cross coupling reaction of Au(C^CH2^N)Cl_2_ with PhCC^−^ (see Schemes 2 and 3, and also Table S1), and with 1,3,5‐triaza‐7‐phosphaadamantane (PTA) (see Figure S30, Supporting Information), following recently reported methods,[^7a^, ^12^] using the M06‐L DFT functional,^[30]^ the Lanl2tz(f) basis set^[31]^ for Au and the and the 6‐311G(d,p) basis set^[32]^ for Cl, O, N, C and H atoms. Full geometry optimizations were performed in the acetone solvent, implicitly reproduced by the polarisable continuum model (PCM).^[33]^ Transition state structures were found by the synchronous transit guided quasi‐Newton method.^[34]^ Vibration frequency calculations, within the harmonic approximation, were performed to check that each optimized geometry corresponded to a minimum or to a first‐order saddle point (for transition state structures) in the potential energy surface, and to evaluate their standard Gibbs free energy values at 298.15 K. All calculations were performed by the Gaussian 09 program package.^[35]^

**X‐Ray diffraction data**: **R1**, **P3a** and **P3b** co‐crystallized by layering *n*‐pentane on a saturated solution of **R1** in DCM. Data were collected on a Bruker D8 Venture single‐crystal X‐ray diffractometer equipped with a CMOS detector (Bruker Photon‐100), a TXS rotating anode with Mo_Kα_ radiation (λ=0.71073 Å) and a Helios optic using the APEX3 software package.^[36]^ Measurements were performed on single crystals coated with perfluorinated ether. The crystals were fixed on top of a kapton micro sampler and frozen under a stream of cold nitrogen. A matrix scan was used to determine the initial lattice parameters. Reflections were corrected for Lorentz and polarisation effects, scan speed, and background using SAINT.^[37]^ Absorption correction, including odd and even ordered spherical harmonics was performed using SADABS.^[38]^ Space group assignments were based upon systematic absences, E statistics, and successful refinement of the structures. The structures were solved using SHELXT with the aid of successive difference Fourier maps, and were refined against all data using SHELXL in conjunction with SHELXLE.^[39]^ Non‐hydrogen atoms were refined with anisotropic displacement parameters. Full‐matrix least‐squares refinements were carried out by minimizing Σ*w*(F_o_^2^–F_c_^2^)^2^ with the SHELXL weighting scheme.^[39a]^ Neutral atom scattering factors for all atoms and anomalous dispersion corrections for the non‐hydrogen atoms were taken from *International Tables for Crystallography*.^[40]^ Images of the crystal structures were generated with Mercury.^[41]^

Deposition Numbers 2082253 (for **R1**, second polymorph), 2082254 (for **P3a**/**P3b** cocrystal), 2082255 (for **R1**, first polymorph) and 2096758 (for **R3**) contain the supplementary crystallographic data for this paper. These data are provided free of charge by the joint Cambridge Crystallographic Data Centre and Fachinformationszentrum Karlsruhe Access Structures service.

## Conflict of interest

The authors declare no conflict of interest.

## Supporting information

As a service to our authors and readers, this journal provides supporting information supplied by the authors. Such materials are peer reviewed and may be re‐organized for online delivery, but are not copy‐edited or typeset. Technical support issues arising from supporting information (other than missing files) should be addressed to the authors.

Supporting Information
