# Supplementary material for: C−C Cross‐Couplings from a Cyclometalated Au(III) C∧ N Complex: Mechanistic Insights and Synthetic Developments
Source: Chemistry. 2021 Aug 28;27(57):14322–34. doi: 10.1002/chem.202102668 (PMC8597034; doi:10.1002/chem.202102668)
Supplement: Supplementary file 1 — Supporting Information [file CHEM-27-14322-s001.pdf]

Chemistry–A European Journal

Supporting Information

C–C Cross-Couplings from a Cyclometalated Au(III) C^N Complex: Mechanistic Insights and Synthetic Developments

Riccardo Bonsignore[+], Sophie R. Thomas[+], Mathilde Rigoulet, Christian Jandl, Alexander Pöthig, Didier Bourissou,* Giampaolo Barone,* and Angela Casini*

Figures

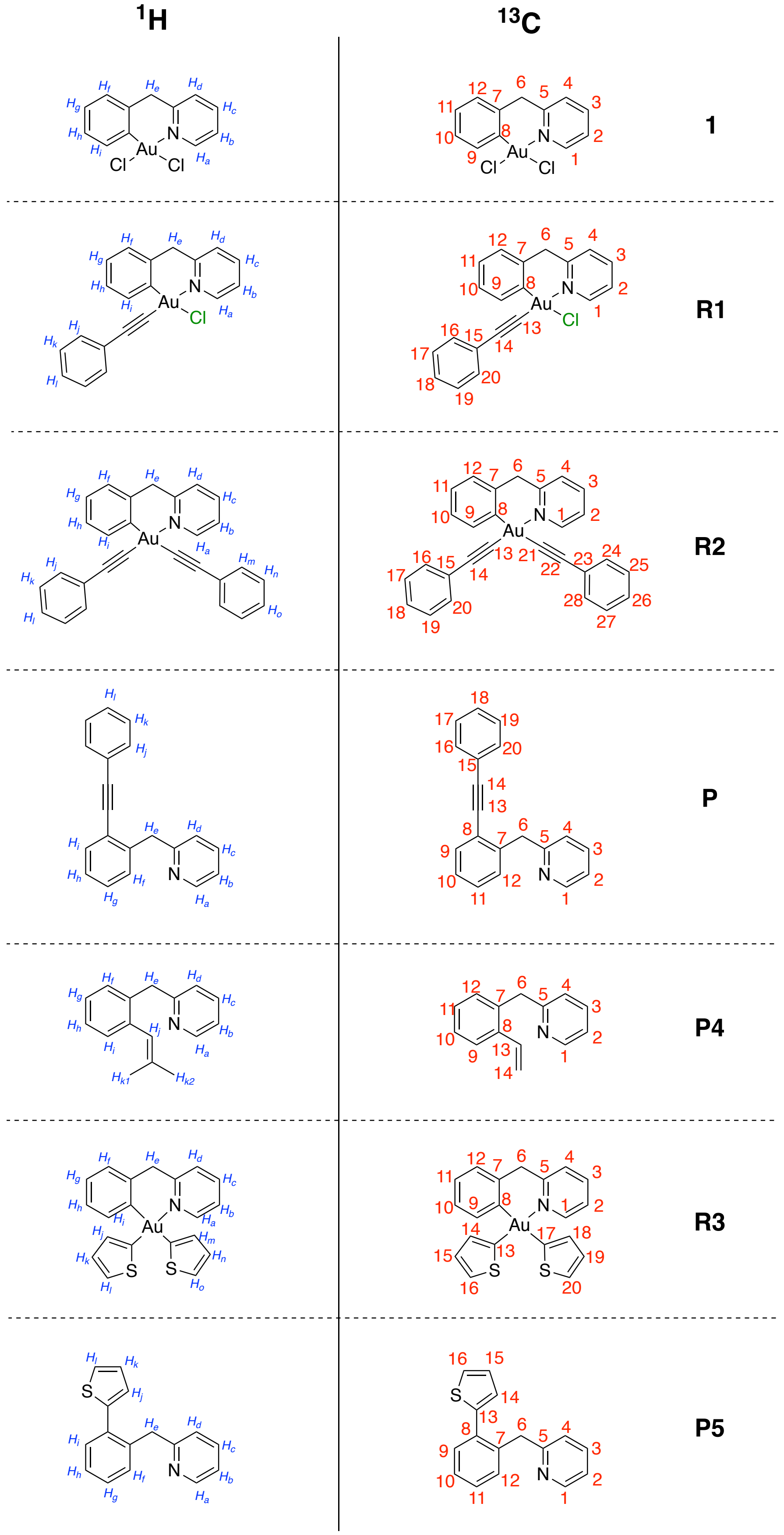

Figure S1. Structures of the species involved in this work along with labelling to assign the corresponding protons and carbons in the ^{1}H and ^{13}C spectra.

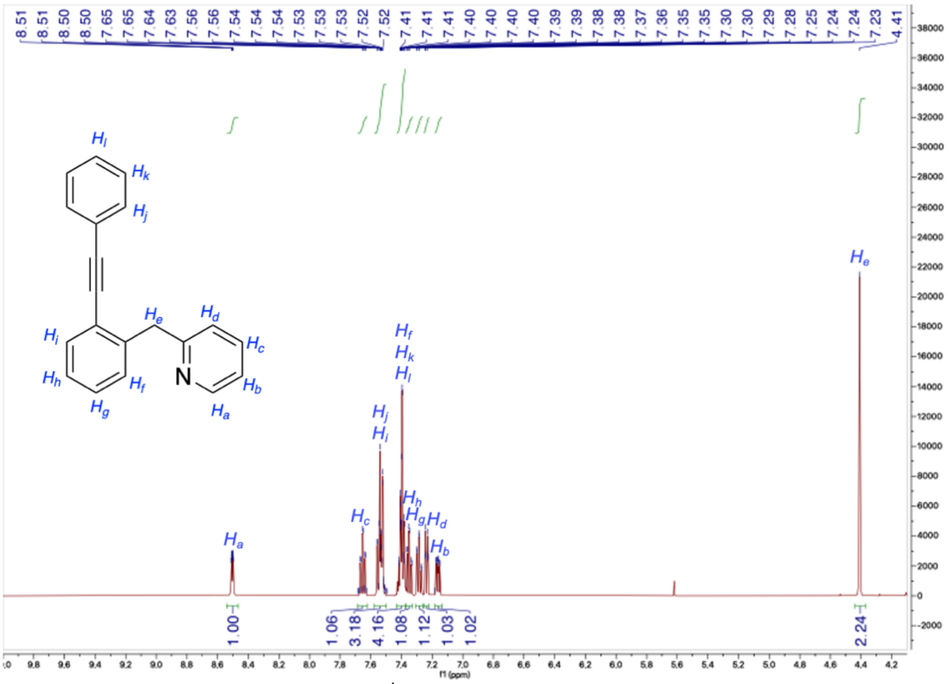

Figure S2. ^{1}H NMR of **P** in acetone-d_6.

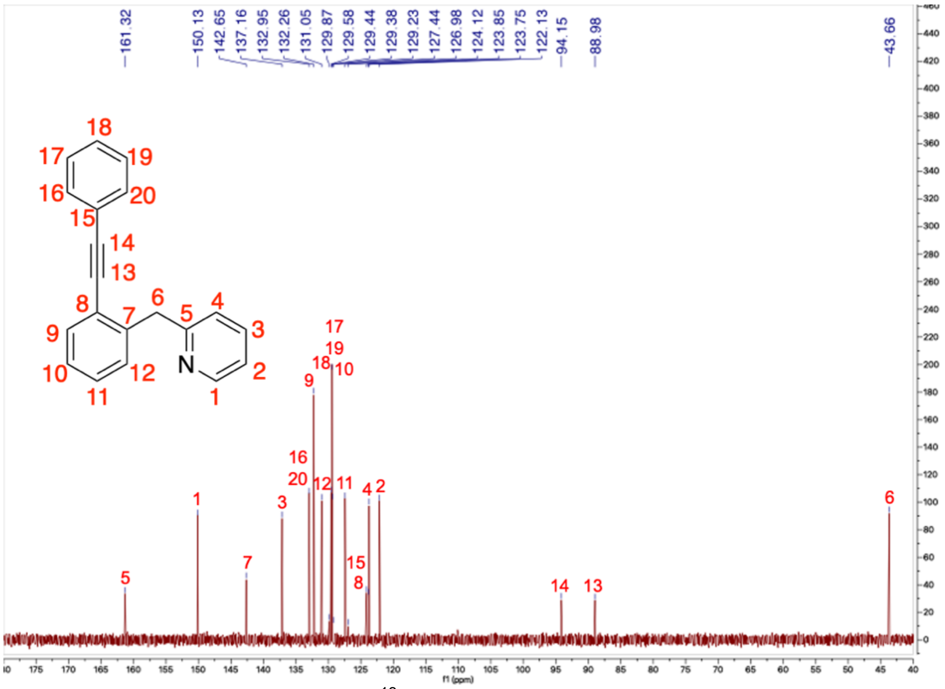

Figure S3. ^{13}C NMR of **P** in acetone-d_6.

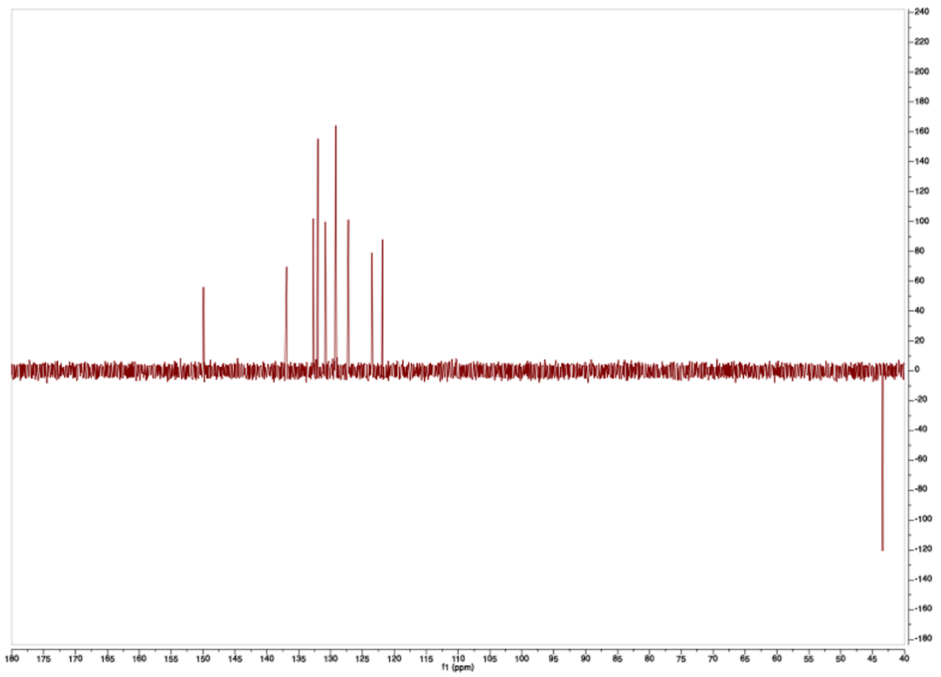

Figure S4. ^{13}C DEPT NMR of **P** in acetone-d_6.

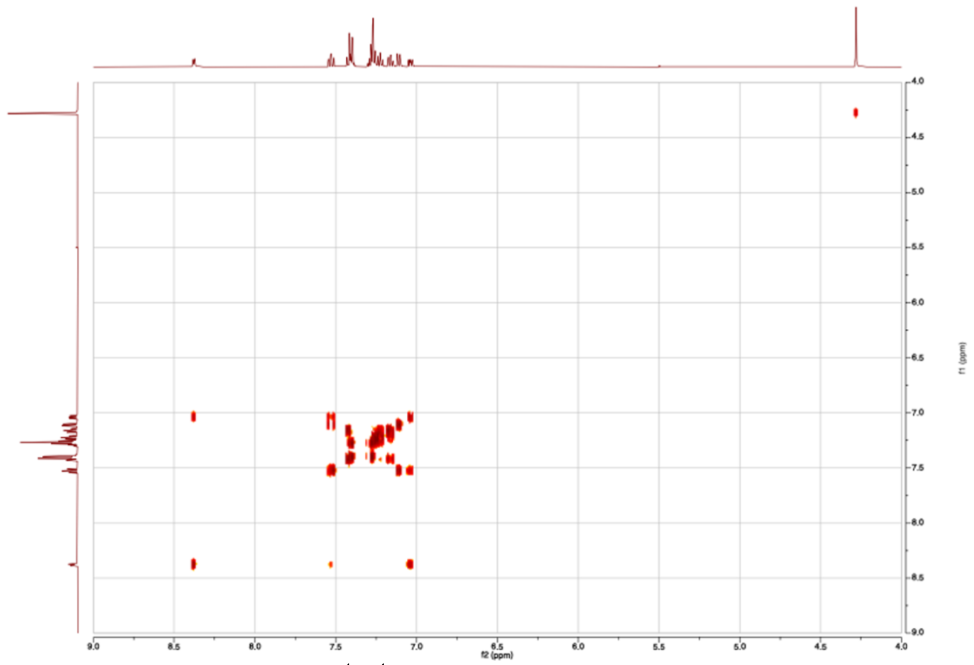

Figure S5. ^{1}H-^{1}H COSY NMR of **P** in acetone-d_6.

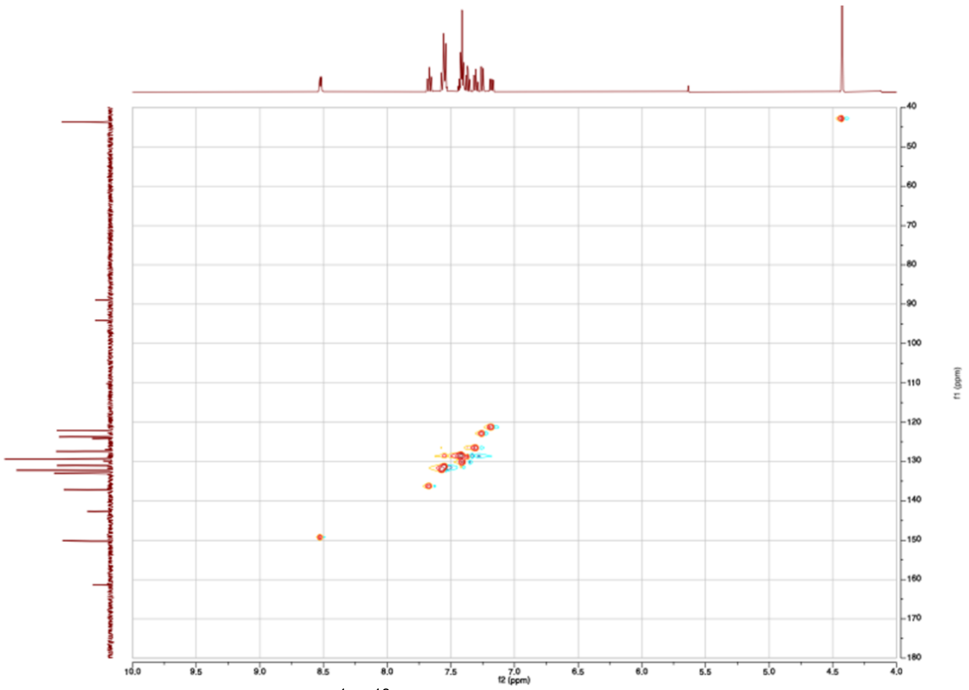

Figure S6. ^{1}H-^{13}C HSQC NMR of **P** in acetone-d_6.

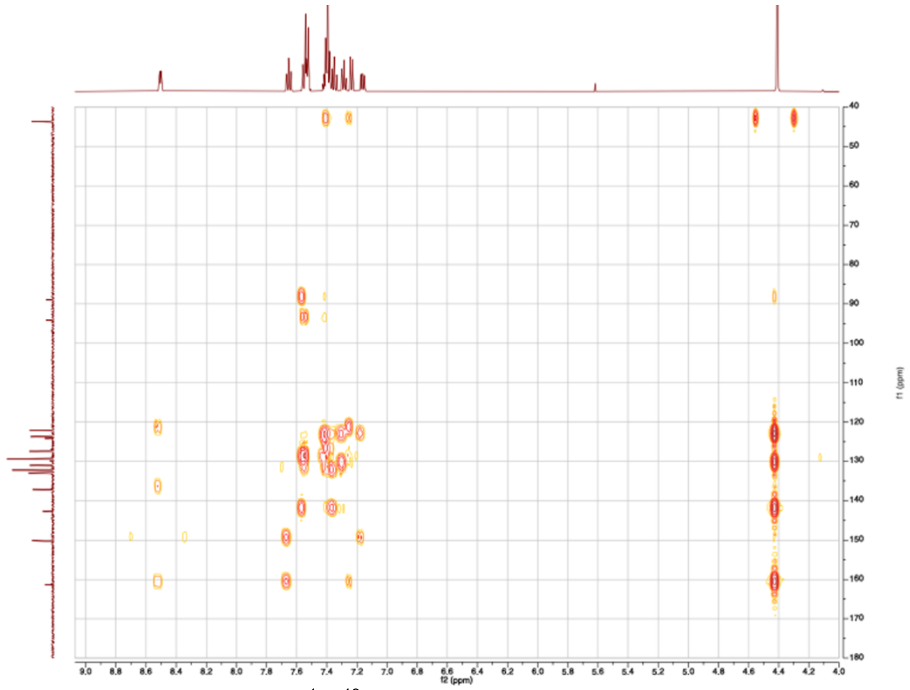

Figure S7. ^{1}H-^{13}C HMBC NMR of **P** in acetone-d_6.

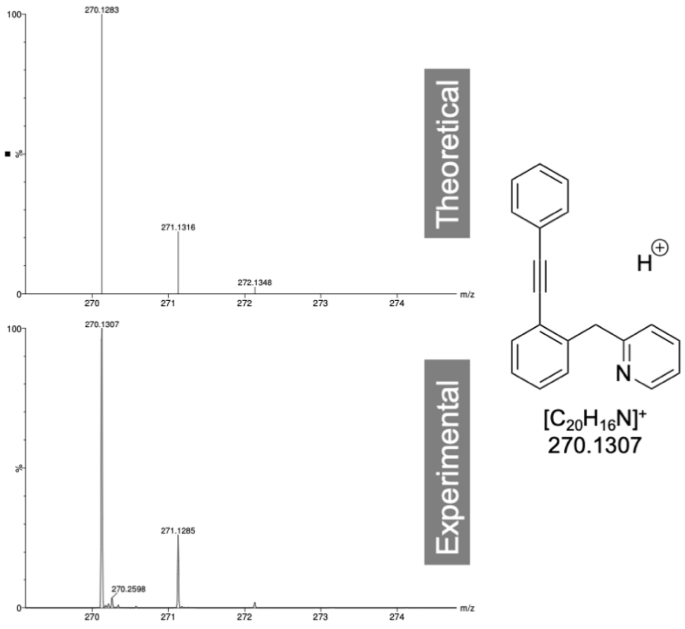

Figure S8. HR-ESI-MS simulated (top) and experimental (below) spectra of **P**.

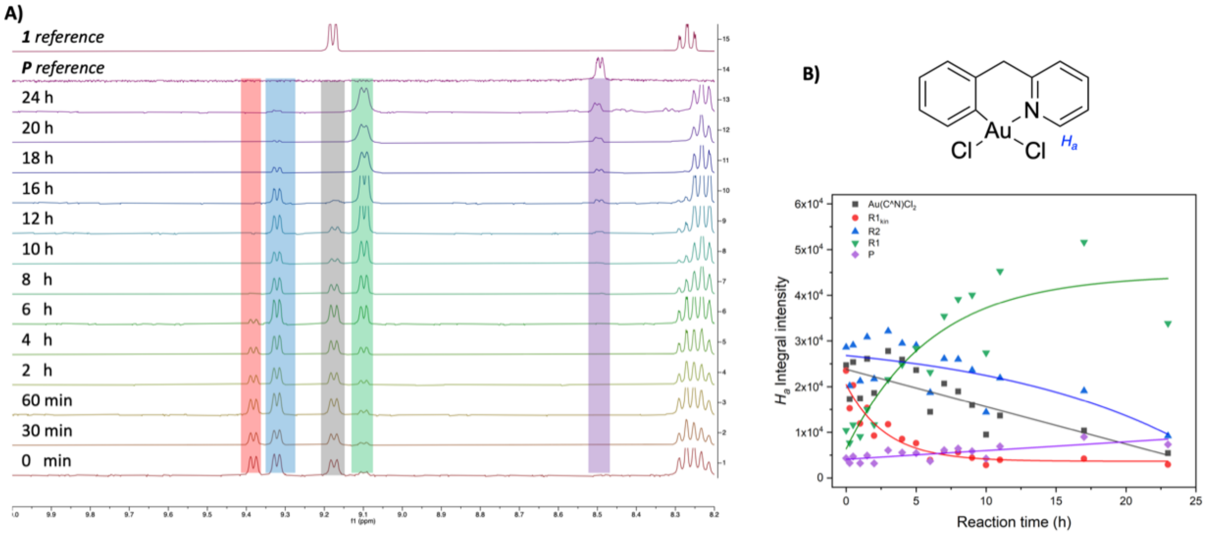

Figure S9. **A**) ^{1}H NMR spectra (between 8.2 and 10 ppm) of the reaction between **1** (1 eq.) and AgPhCC (2 eq.) in DMSO-d_6 followed over 24 h at r.t.. The spectrum of the starting material **1** and of the final purified product **P** are reported as reference. **B**) Structure of **1** highlighting the proton ortho to the pyridyl N (H_a), and evolution of the intensities of the integrals of H_a chemical shifts over 24 h. Fitting lines have been included as a visual aid to follow the trend over the selected time window.

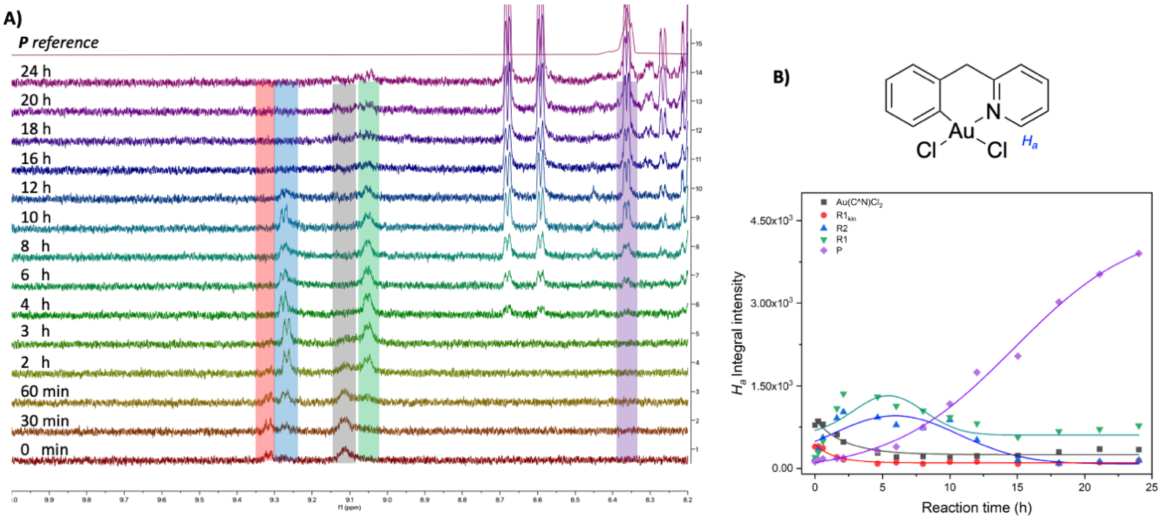

Figure S10. **A**) ^{1}H NMR spectra (between 8.2 and 10 ppm) of the reaction between **1** (1 eq.) and AgPhCC (2 eq.) in MeOH-*d*$_4$ followed over 24 h at r.t.. The spectrum of the final purified product **P** is reported as reference. The reference spectrum for **1** cannot be reported due to poor solubility in this solvent. **B**) Structure of **1** highlighting the proton ortho to the pyridyl N (H_a), and evolution of the intensities of the integrals of H_a chemical shifts over 24 h. Fitting lines have been included as a visual aid to follow the trend over the selected time window.

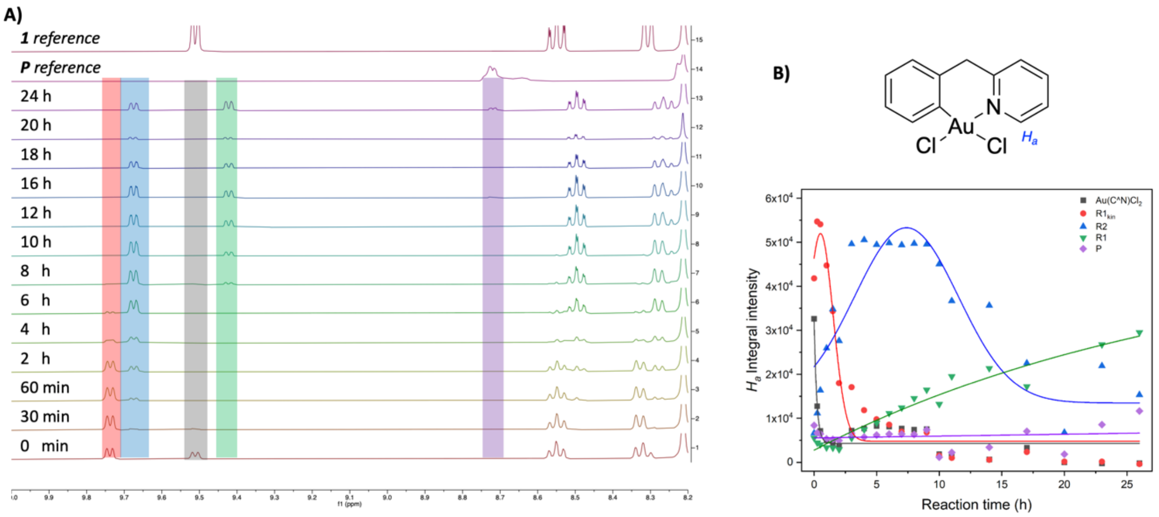

Figure S11. **A**) ^{1}H NMR spectra (between 8.2 and 10 ppm) of the reaction between **1** (1 eq.) and AgPhCC (2 eq.) in DMF-*d*$_7$ followed over 24 h at r.t.. The spectrum of the starting material **1** and of the final purified product **P** are reported as reference. **B**) Structure of **1** highlighting the proton ortho to the pyridyl N (H_a), and evolution of the intensities of the integrals of H_a chemical shifts over 24 h. Fitting lines have been included as a visual aid to follow the trend over the selected time window.

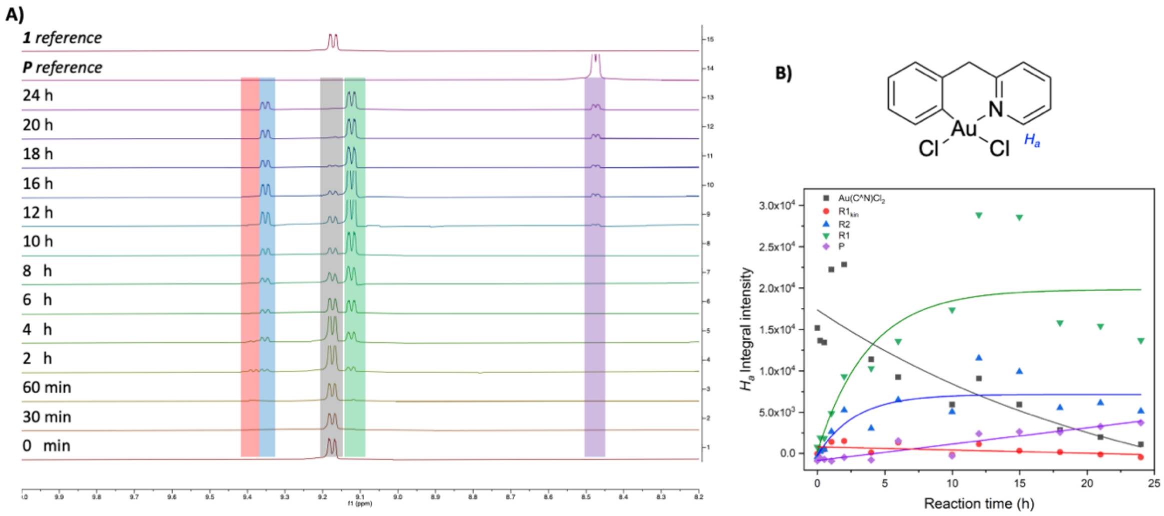

Figure S12. **A**) ^{1}H NMR spectra (between 8.2 and 10 ppm) of the reaction between **1** (1 eq.) and AgPhCC (2 eq.) in MeCN-d_3 followed over 24 h at r.t.. The spectrum of the starting material **1** and of the final purified product **P** are reported as reference. **B**) Structure of **1** highlighting the proton ortho to the pyridyl N (H_a), and evolution of the intensities of the integrals of H_a chemical shifts over 24 h. Fitting lines have been included as a visual aid to follow the trend over the selected time window.

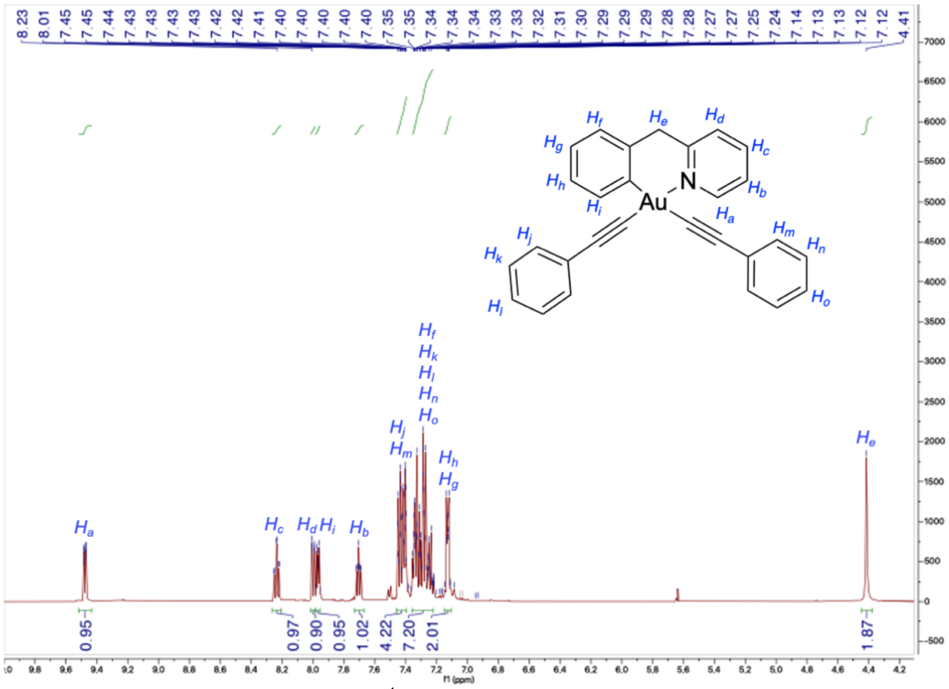

Figure S13. ^{1}H NMR of **R2** in acetone-d_6.

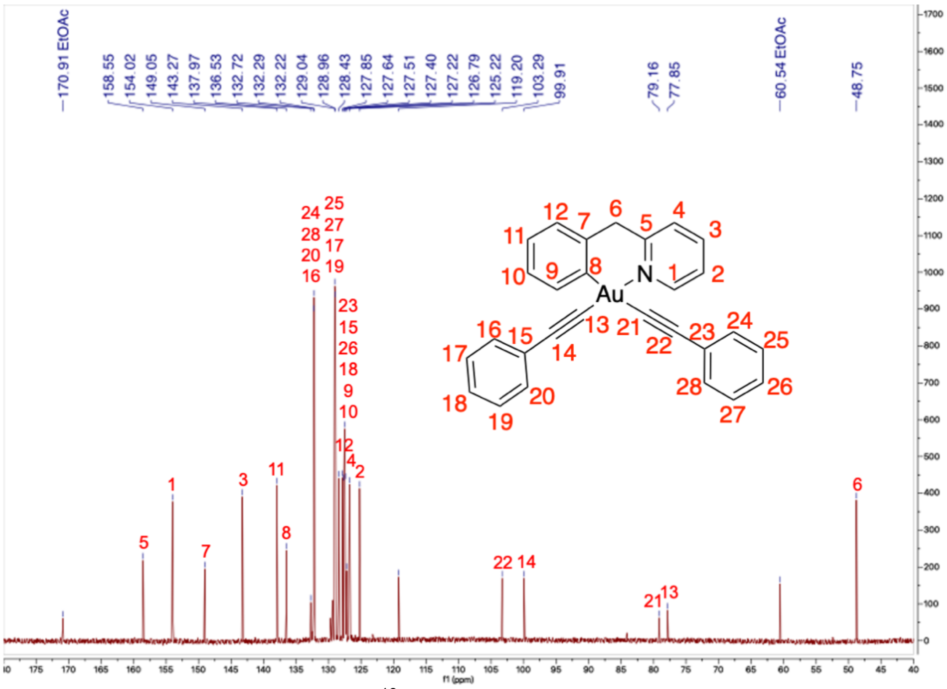

Figure S14. ^{13}C NMR of **R2** in acetone-*d*$_6$.

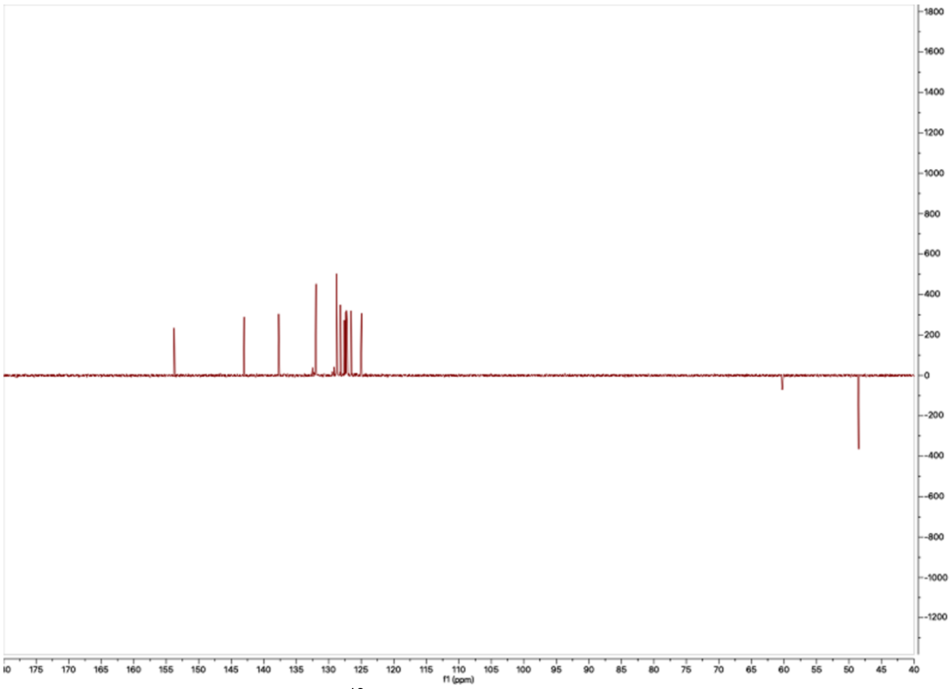

Figure S15. ^{13}C DEPT NMR of **R2** in acetone-*d*$_6$.

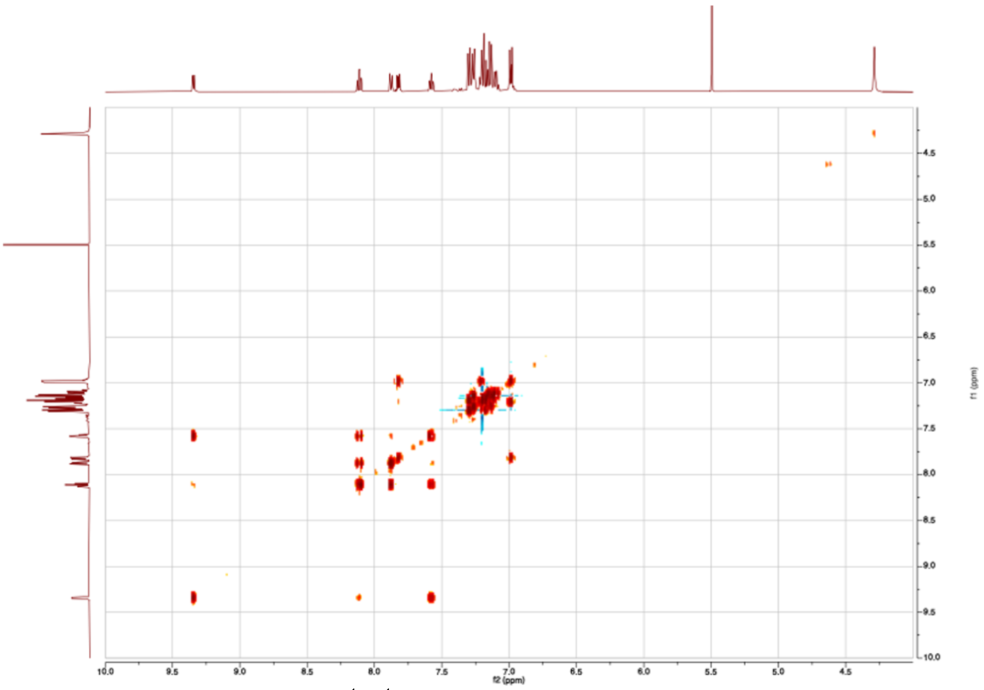

Figure S16. ^{1}H-^{1}H COSY NMR of **R2** in acetone-d_6.

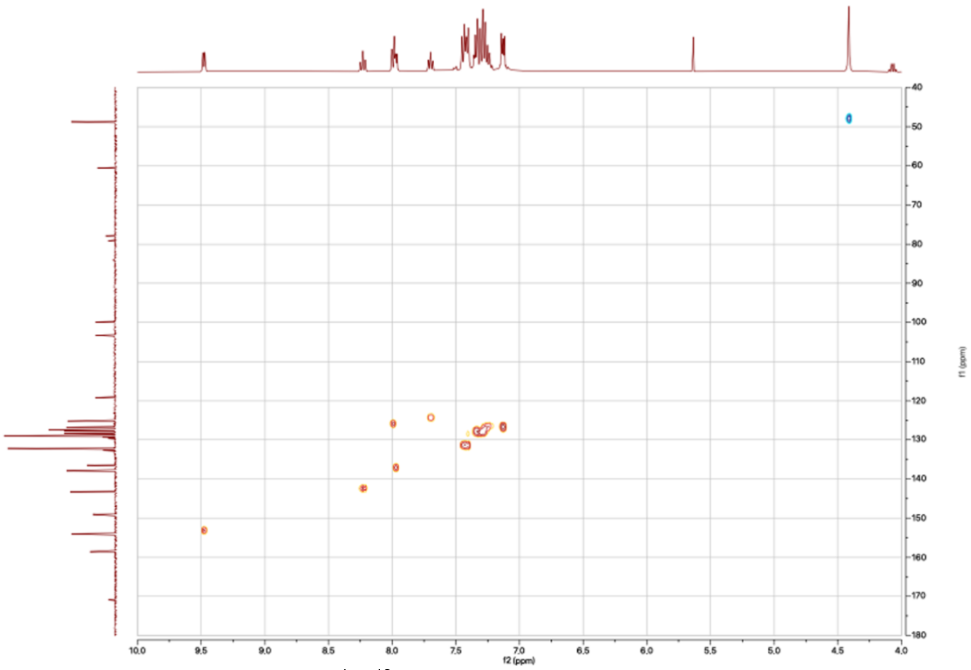

Figure S17. ^{1}H-^{13}C HSQC NMR of **R2** in acetone-d_6.

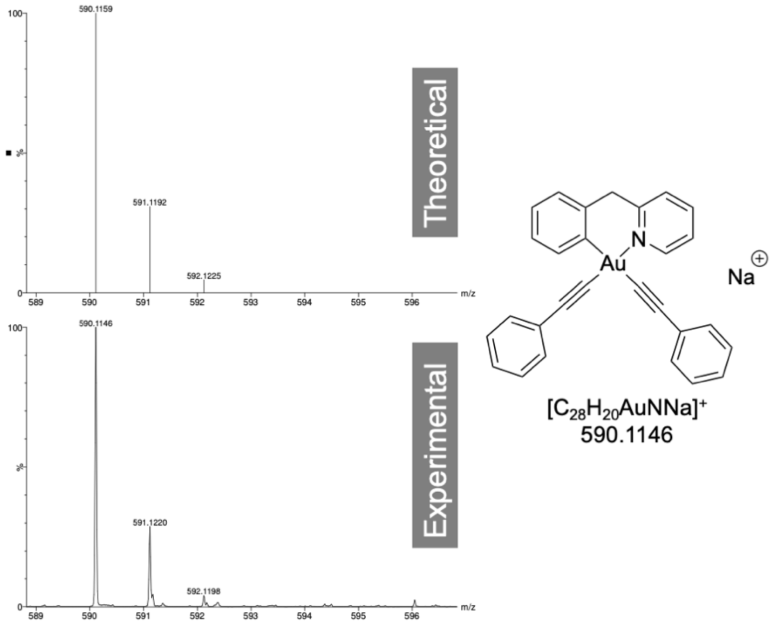

Figure S18. HR-ESI-MS simulated (top) and experimental (below) spectra of **R2.**

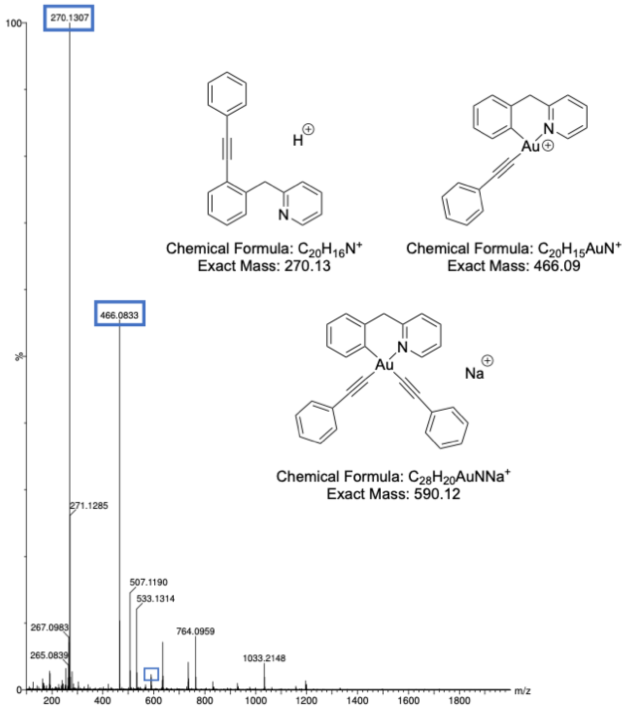

Figure S19**.** HR-ESI-MS of crude obtained by reacting **1** with AgPhCC in a 1:3 ratio in acetone (25 mL).

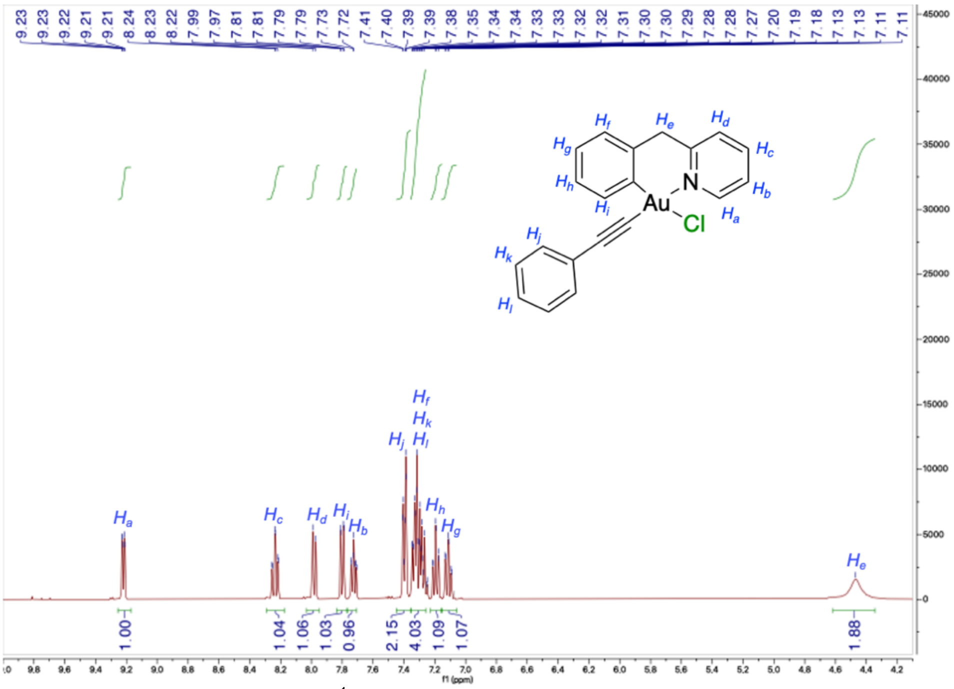

Figure S20. ^{1}H NMR spectrum of **R1** in acetone-d_6.

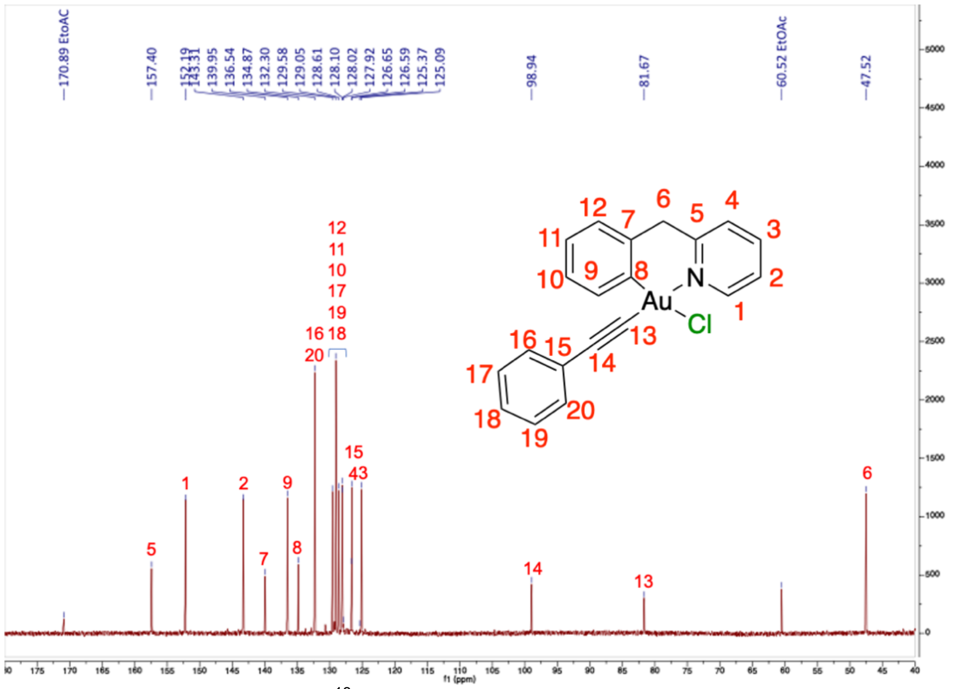

Figure S21. ^{13}C NMR spectrum of **R1** in acetone-d_6.

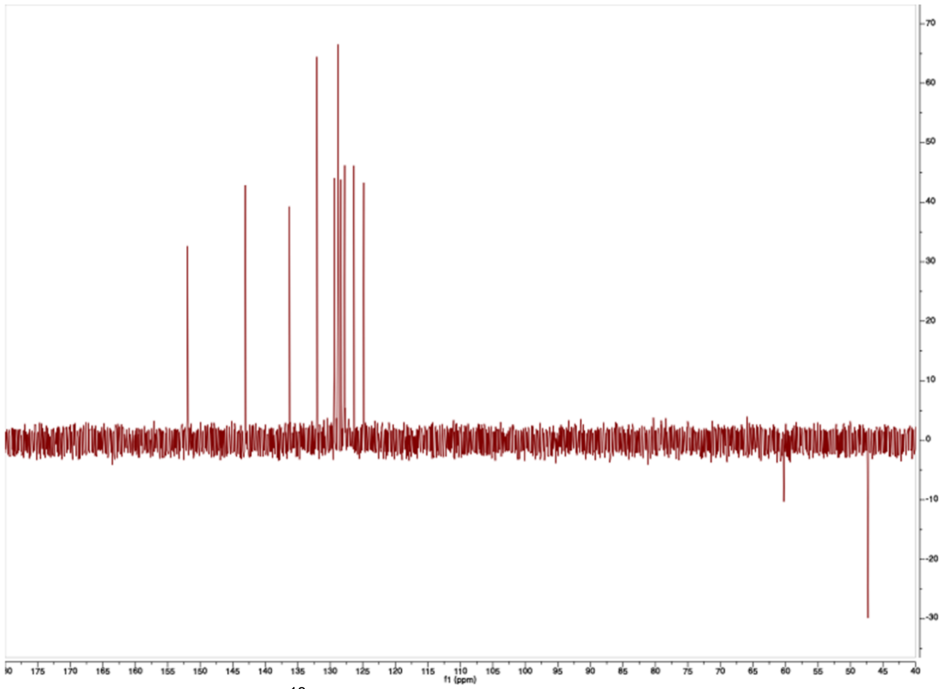

Figure S22. ^{13}C DEPT NMR spectrum of **R1** in acetone-d_6.

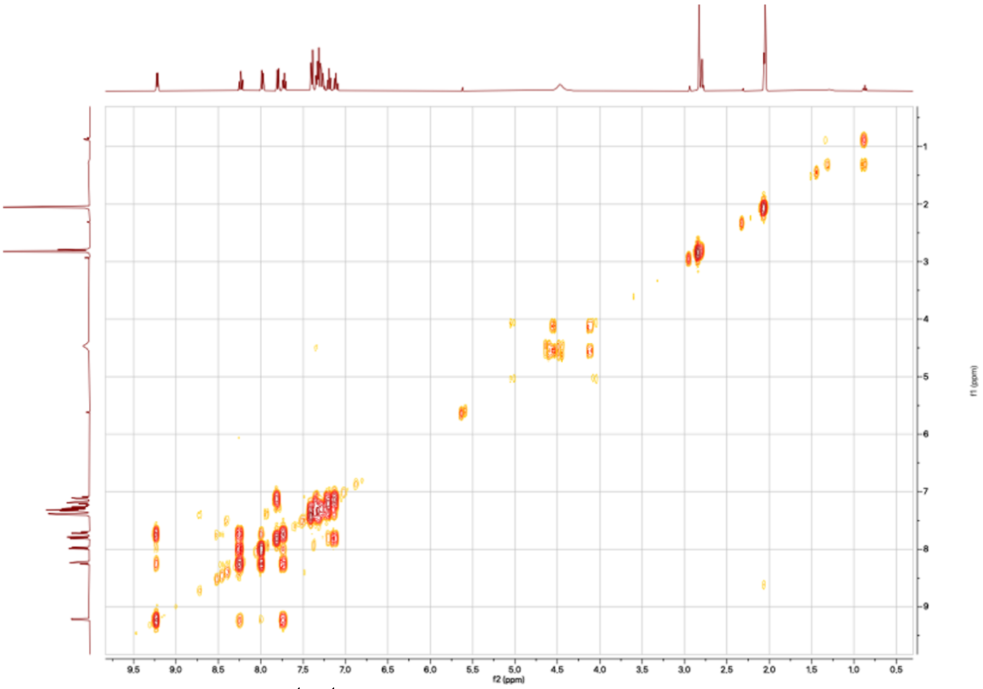

Figure S23. ^{1}H-^{1}H COSY NMR spectrum of **R1** in acetone-d_6.

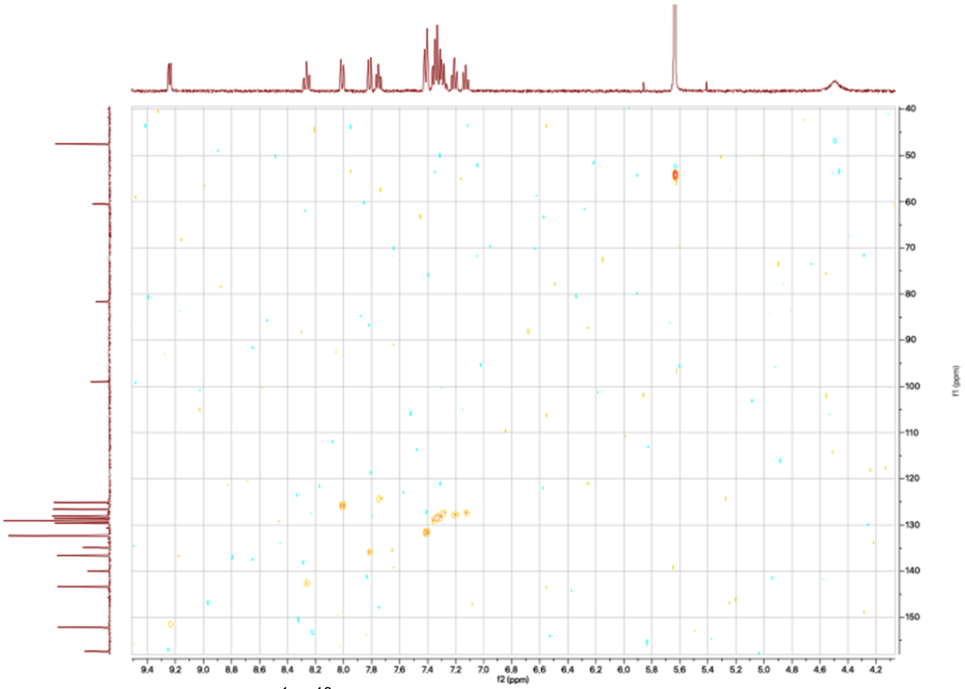

Figure S24. ^{1}H-^{13}C HSQC NMR spectrum of **R1** in acetone-d_6.

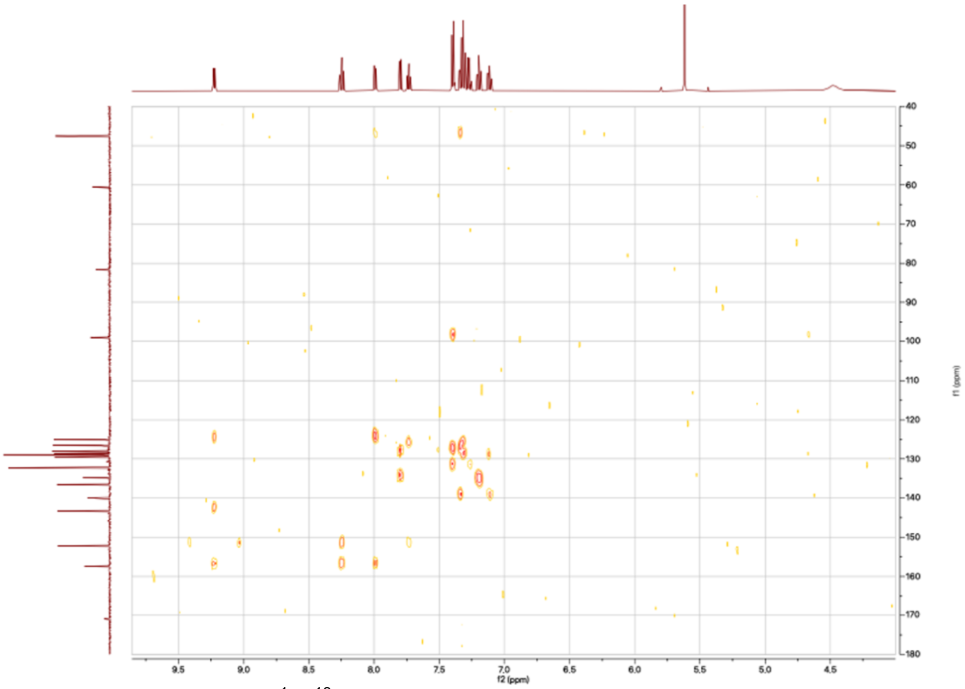

Figure S25. ^{1}H-^{13}C HMBC NMR spectrum of **R1** in acetone-d_6.

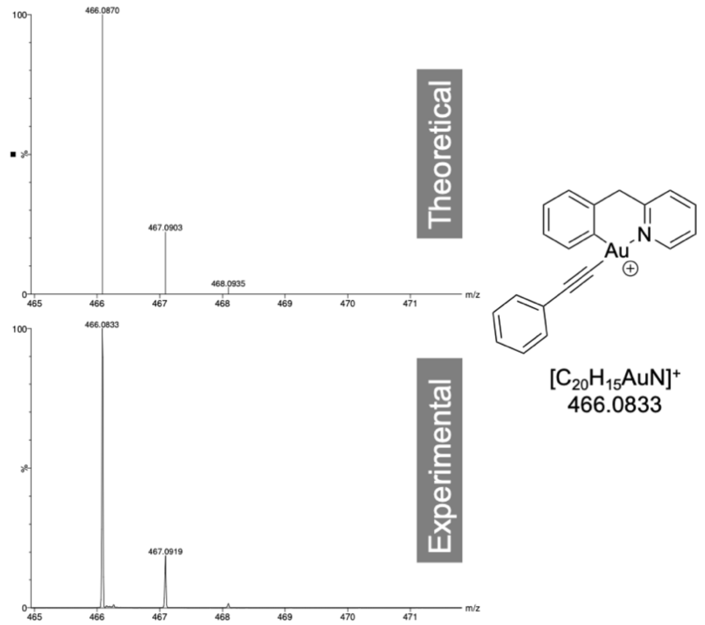

Figure S26. HR-ESI-MS simulated (top) and experimental (below) spectra of **R1**.

DFT calculations

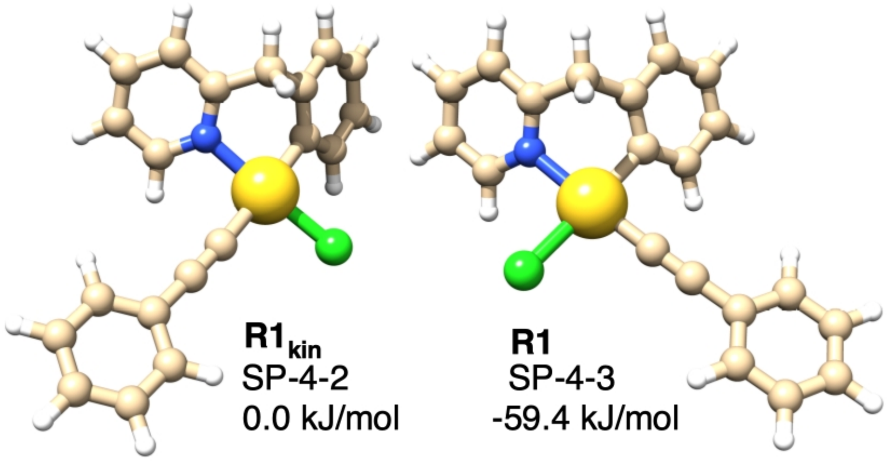

Figure S27. Structure and relative stability in acetone of the possible stereoisomers of the [Au(C^N)Cl(PhCC)] complexes (**R1**$_{\mathbf{kin}}$ and **R1**), obtained by DFT calculations.

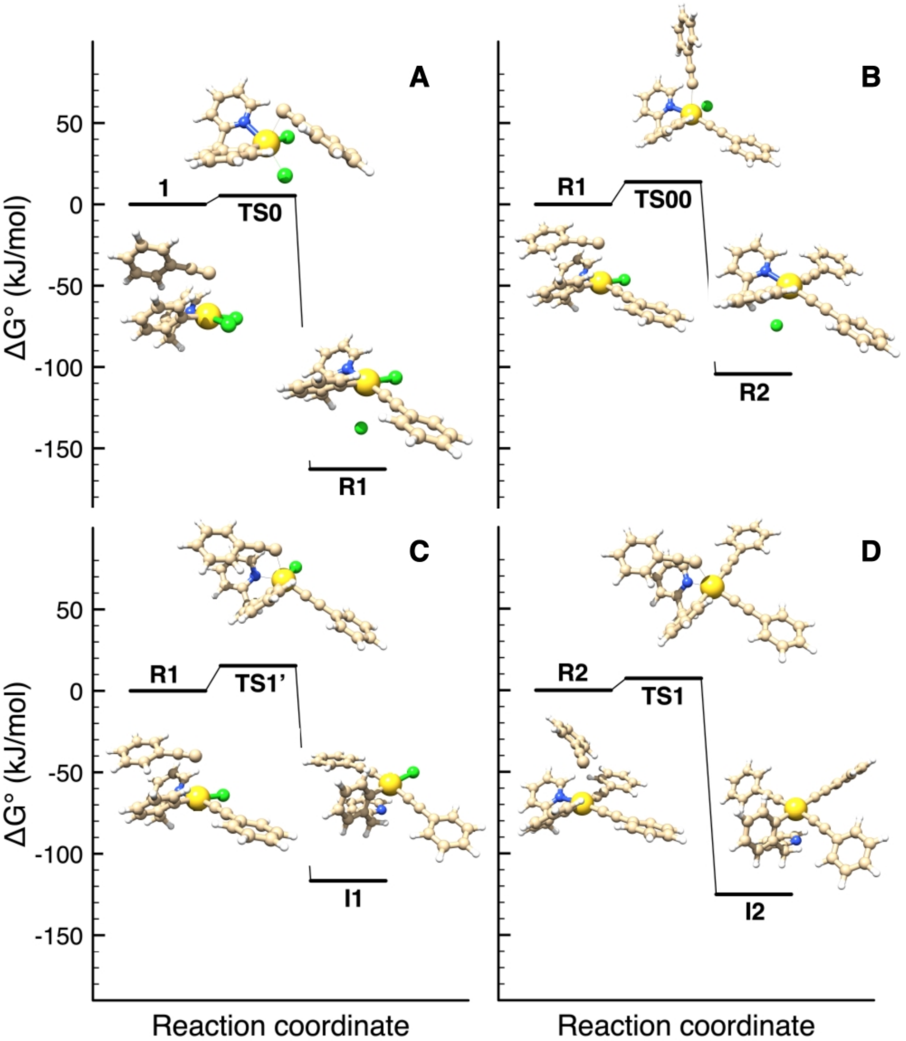

Figure S28. Reaction steps of compound **1** to **R1** (A), **R1** to **R2** (B), **R1** to **I1** (C) and **R2** to **I2** (D). Structures and energies have been obtained by DFT calculations.

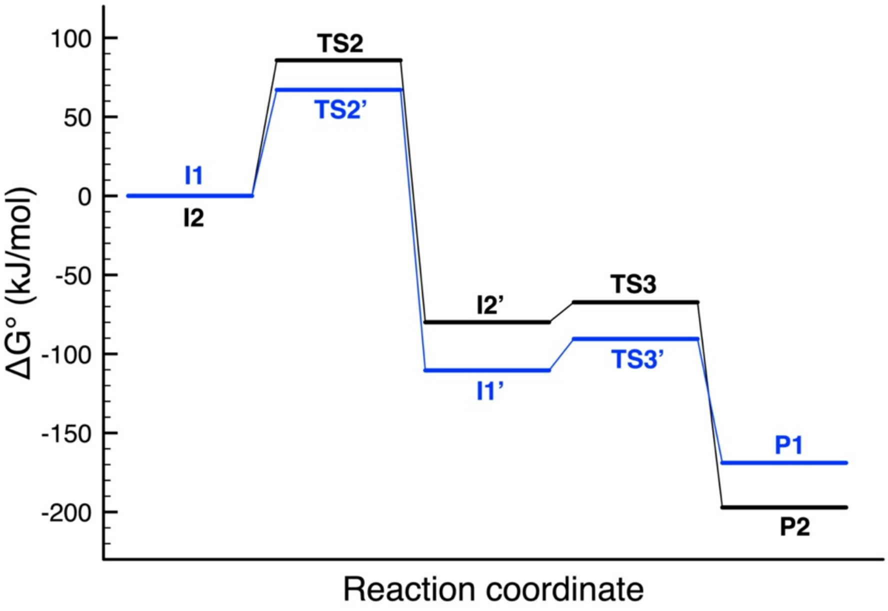

Figure S29. Energy comparison between the two C−C cross-coupling reaction pathways, obtained by DFT calculations (see Figure 5 and Scheme 3).

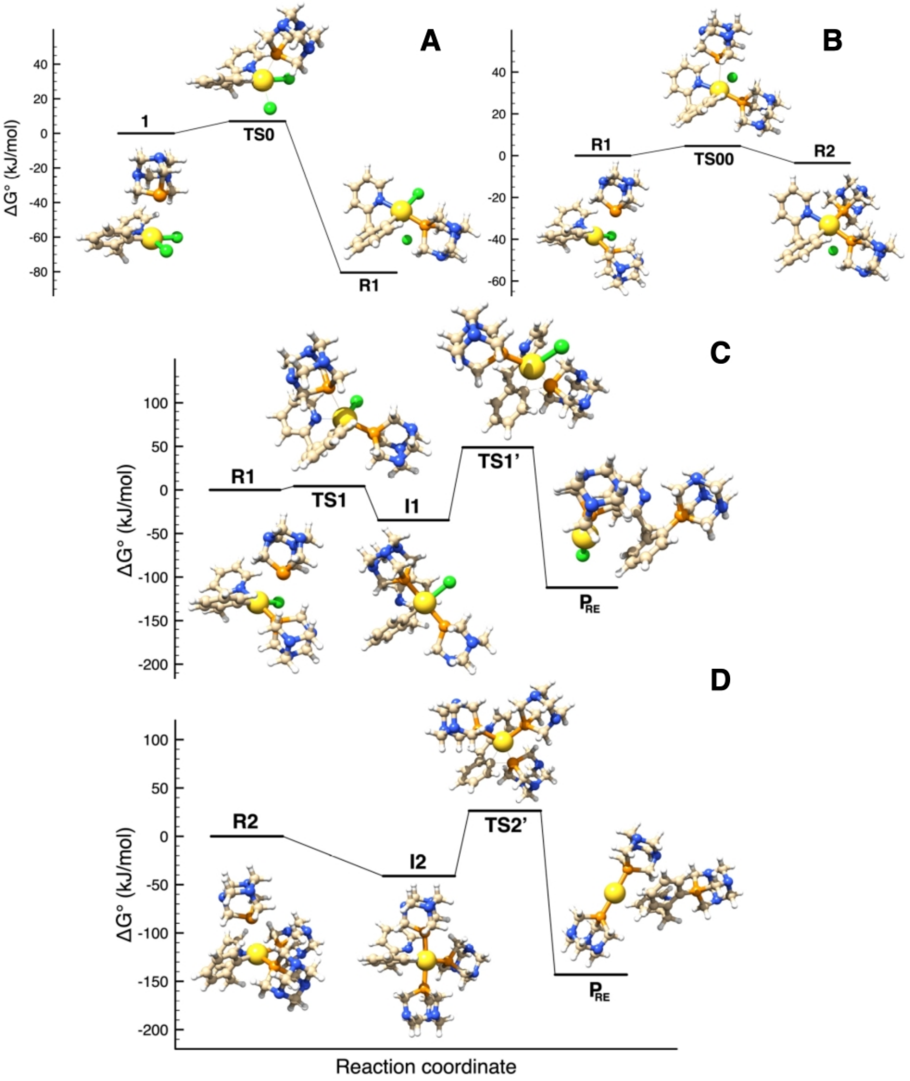

Figure S30. Species involved along the C-P cross-coupling reaction pathway of compound **1**, undergoing Cl^-/PTA substitution (to give **R1** (**A**) or **R2** (**B**)) and reductive elimination (**C** and **D**). Structures and energies have been obtained by DFT calculations.

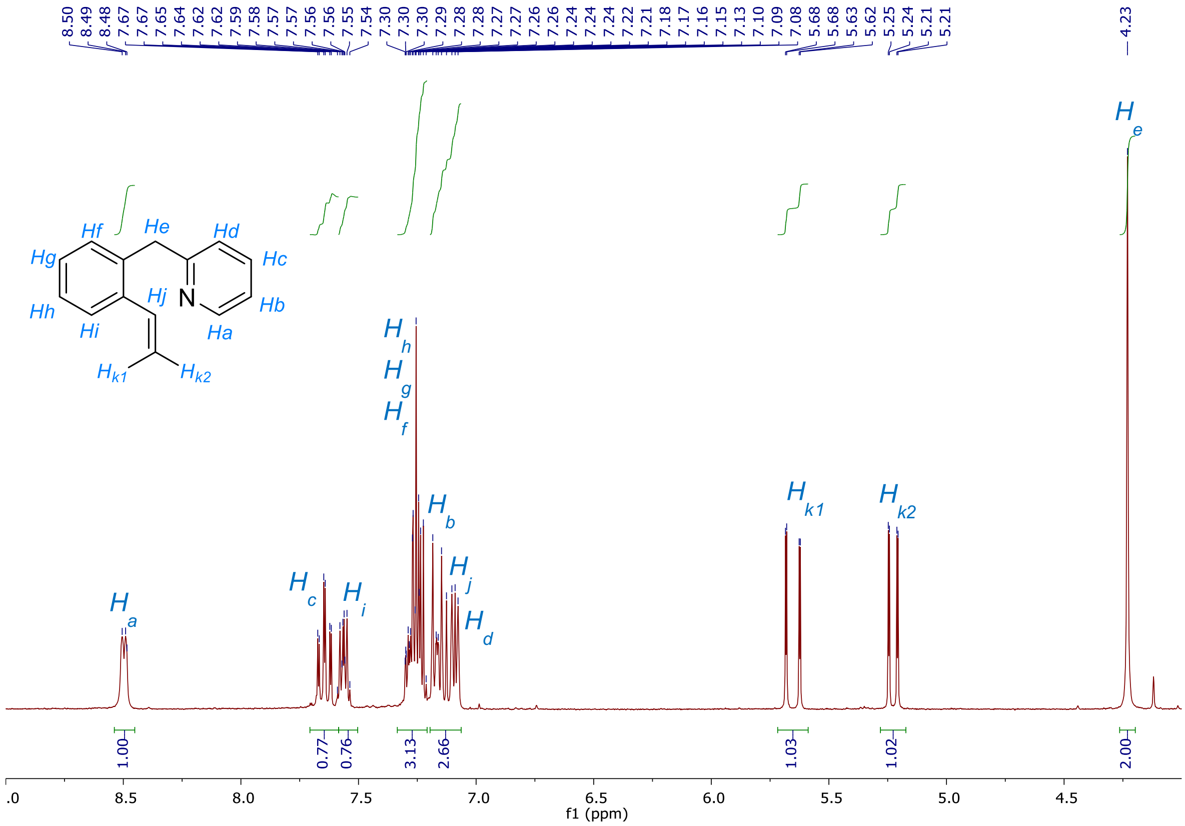

Figure S31. ^{1}H NMR spectrum of **P4** in acetone-d_6.

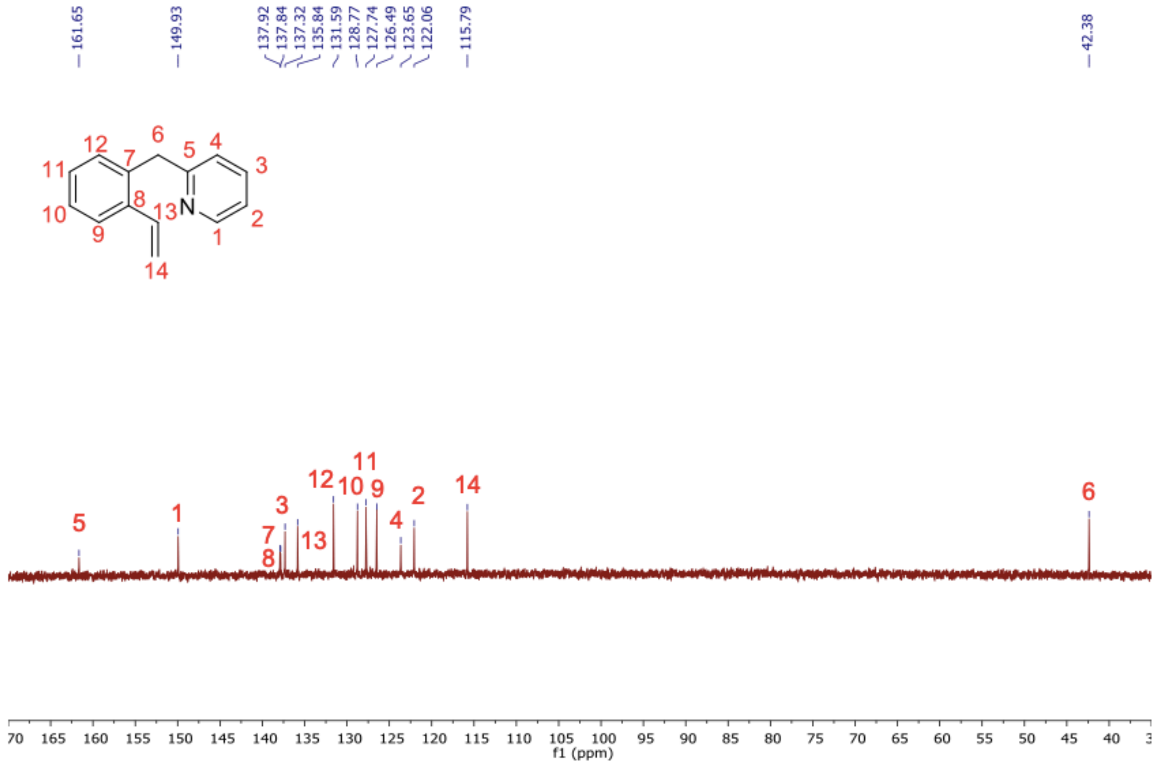

Figure S32. ^{13}C NMR spectrum of **P4** in acetone-d_6.

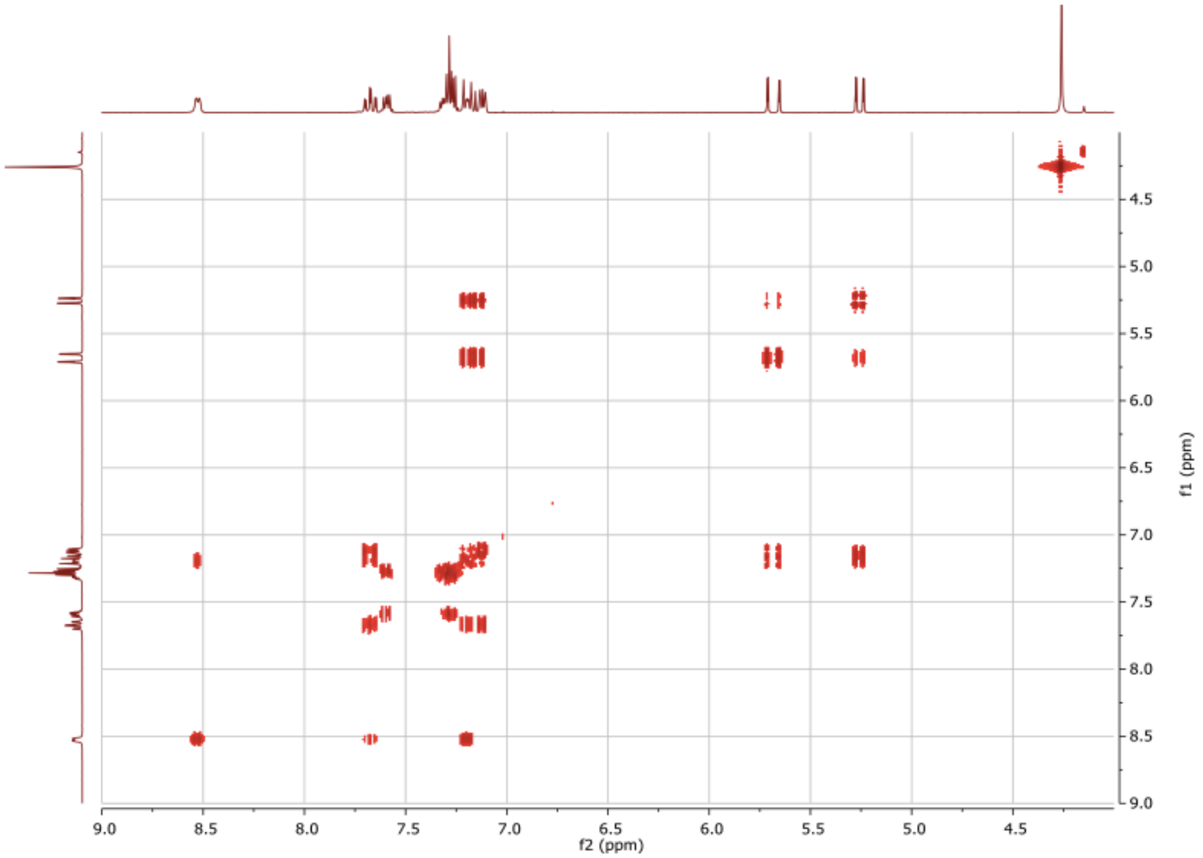

Figure S33. ^{1}H-^{1}H COSY NMR spectrum of **P4** in acetone-d_6.

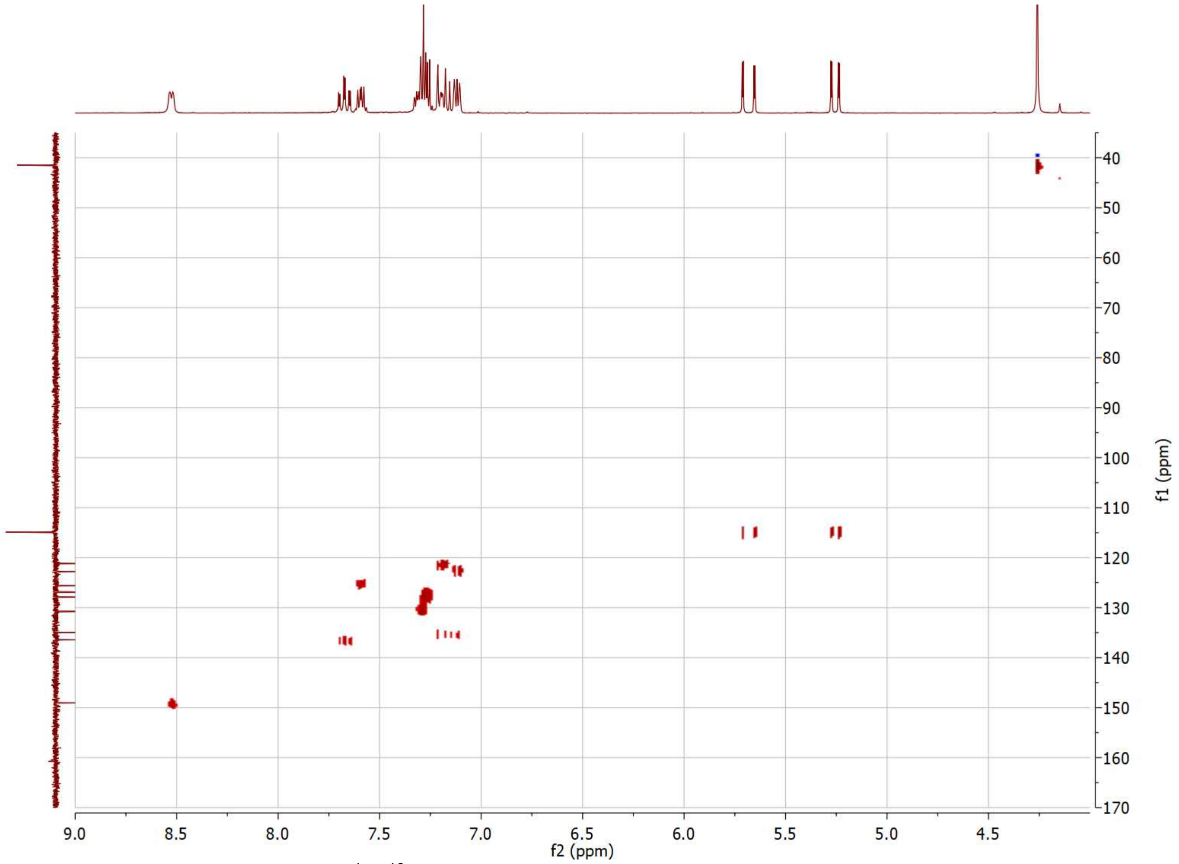

Figure S34. ^{1}H-^{13}C HSQC NMR spectrum of **P4** in acetone-d_6.

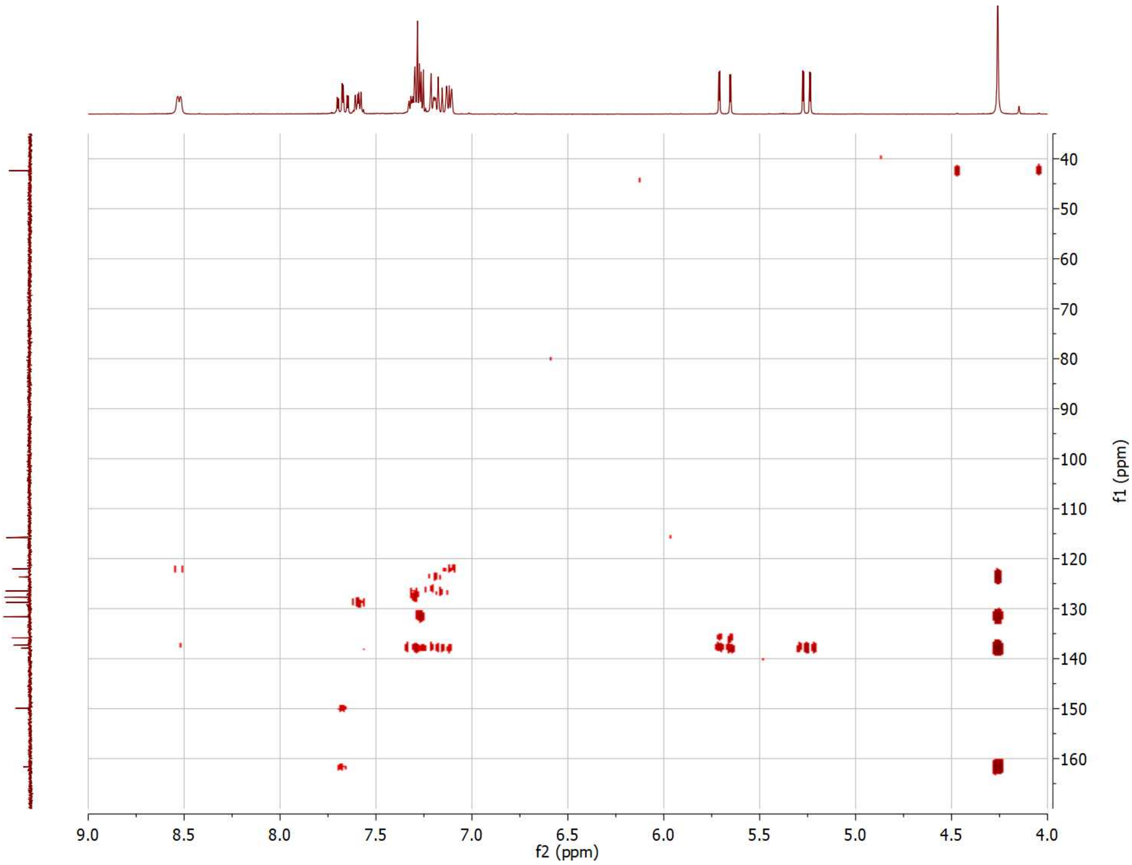

Figure S35. ^{1}H-^{13}C HMBC NMR spectrum of **P4** in acetone-d_6.

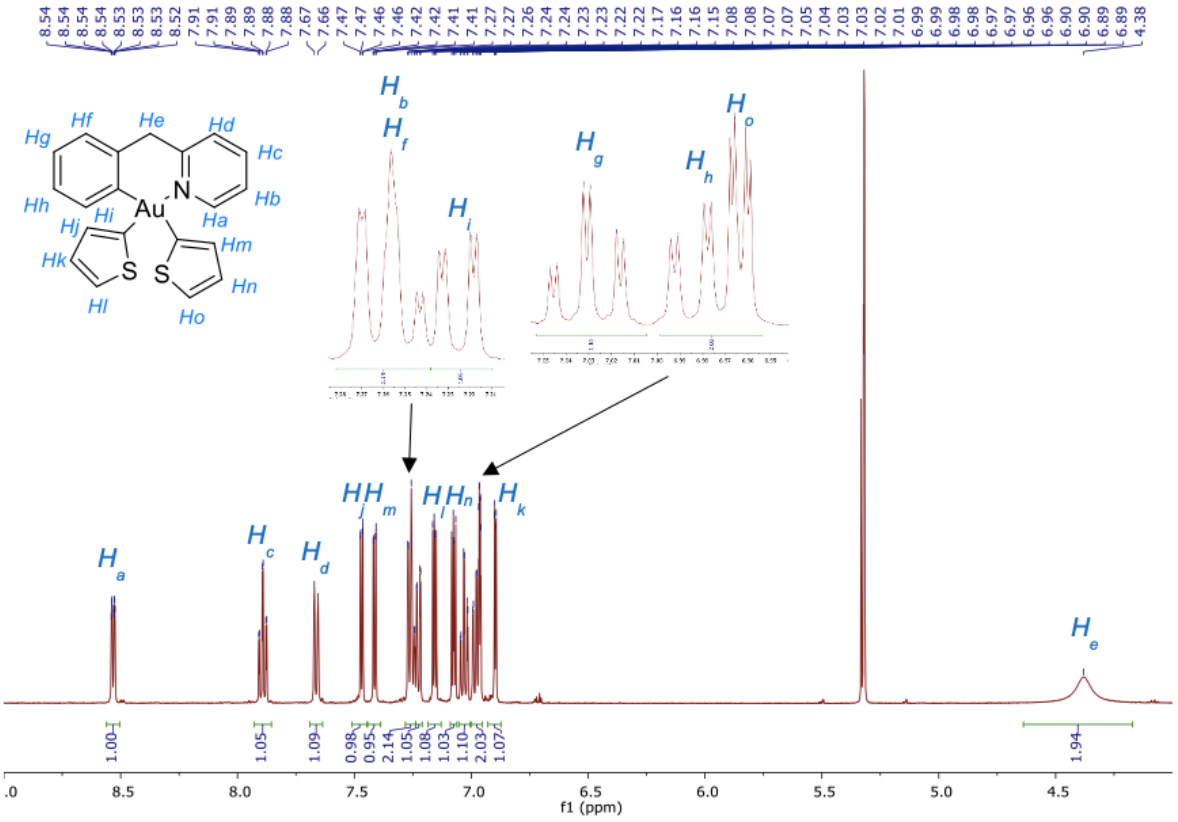

Figure S36. ^{1}H NMR spectrum of **R3** in CD_2Cl_2.

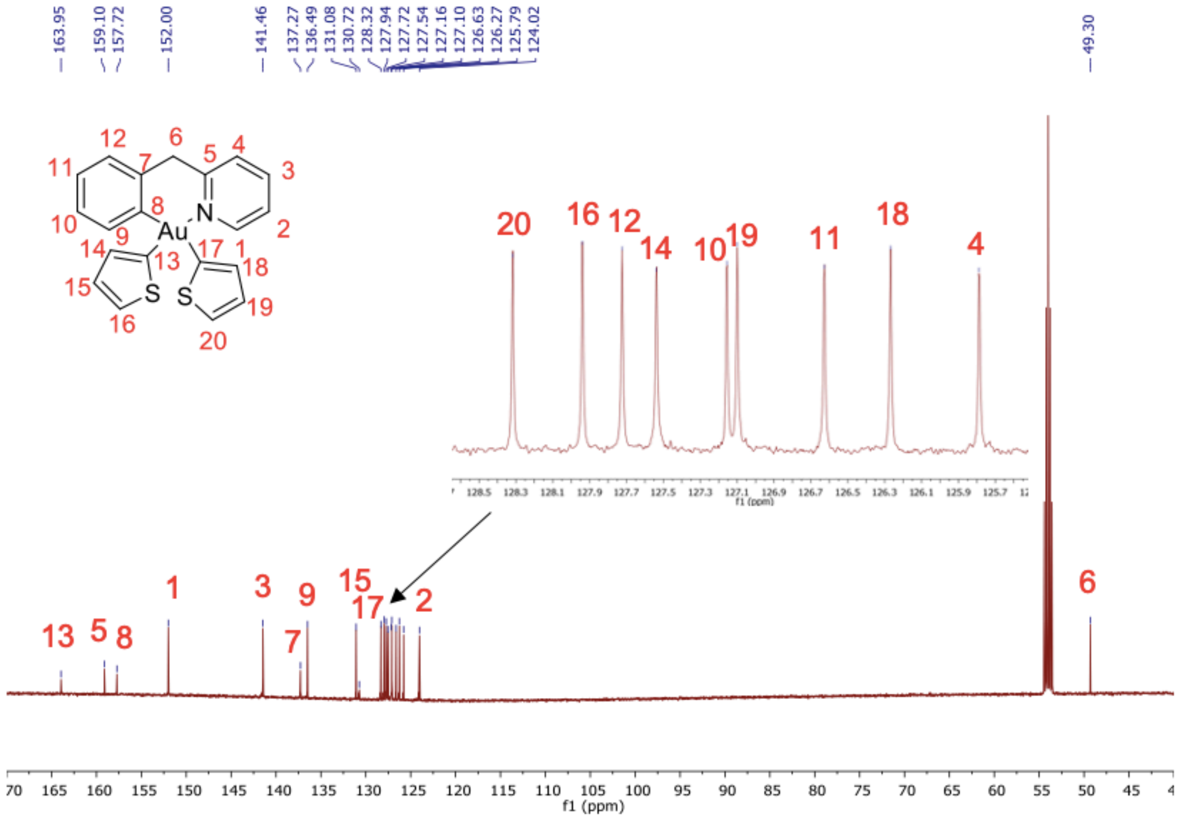

Figure S37. ^{13}C NMR spectrum of **R3** in CD_2Cl_2.

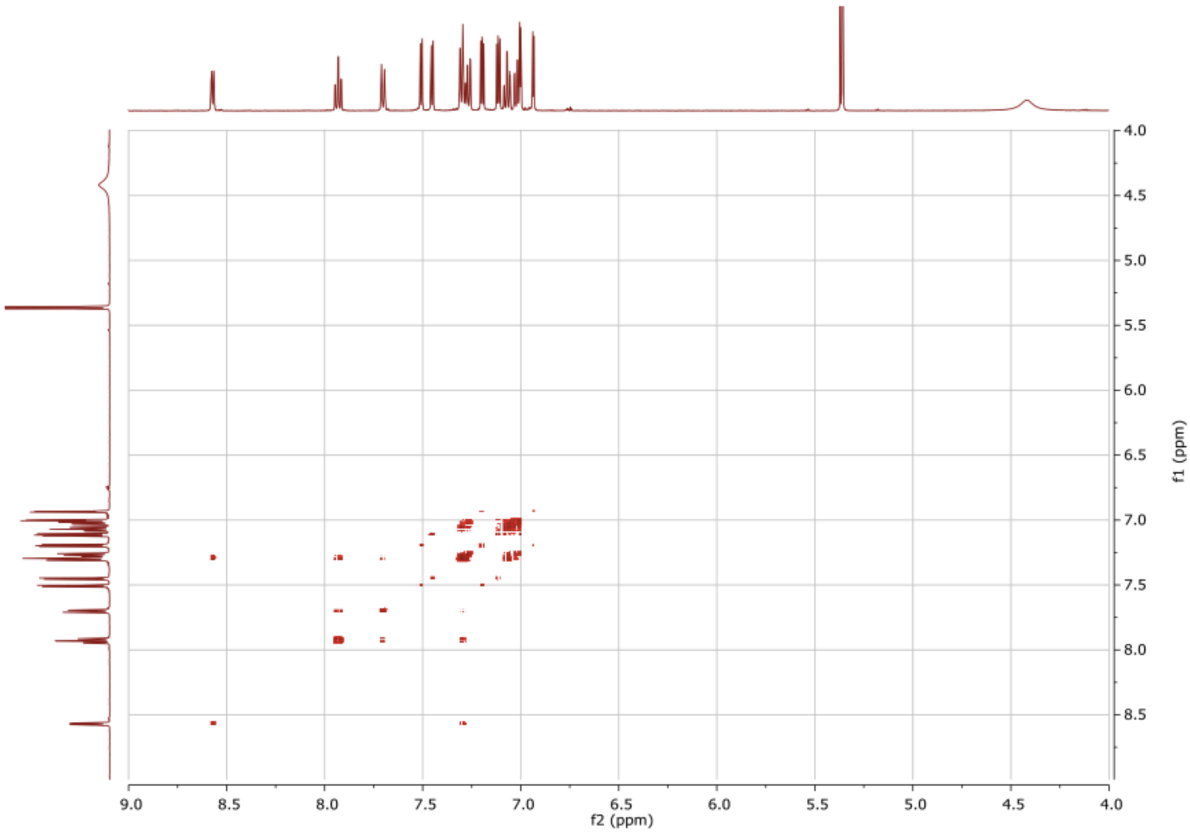

Figure S38. ^{1}H-^{1}H COSY NMR of **R3** in CD_2Cl_2.

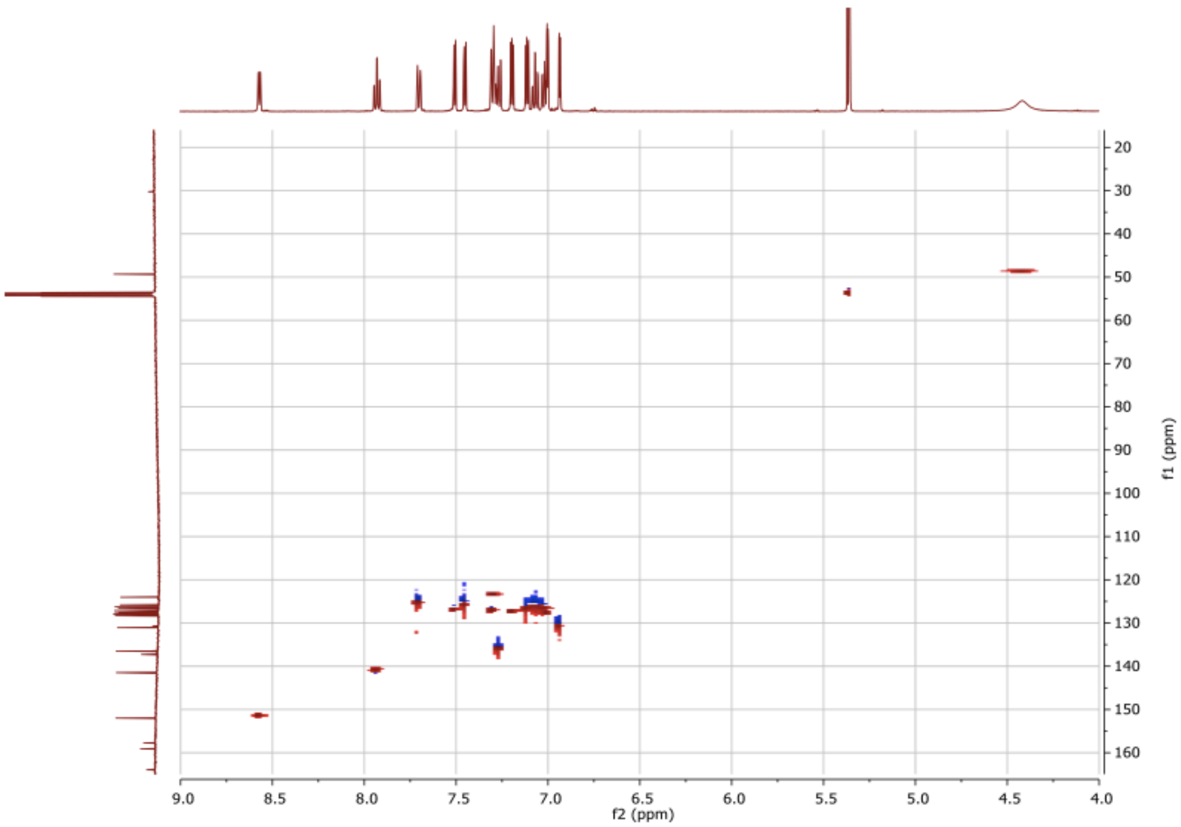

Figure S39. ^{1}H-^{13}C HSQC NMR of **R3** in CD_2Cl_2.

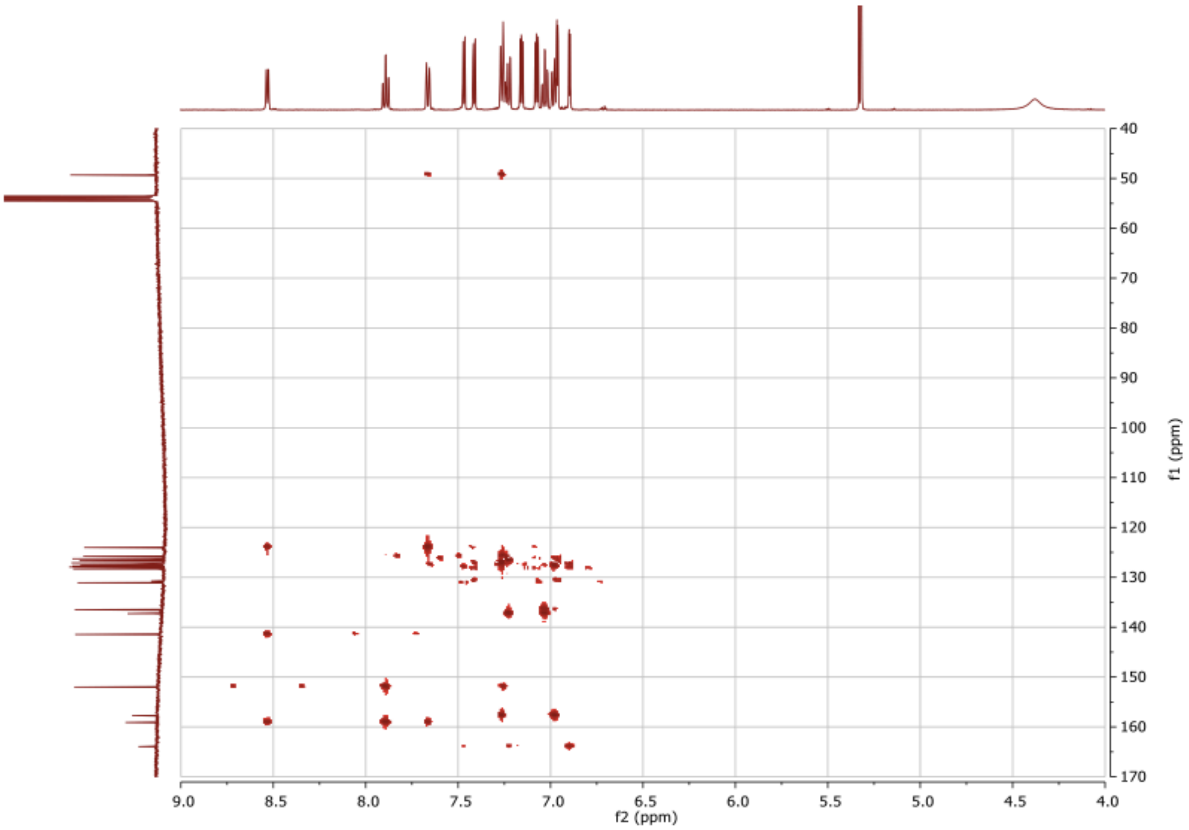

Figure S40. ^{1}H-^{13}C HMBC NMR of **R3** in CD_2Cl_2.

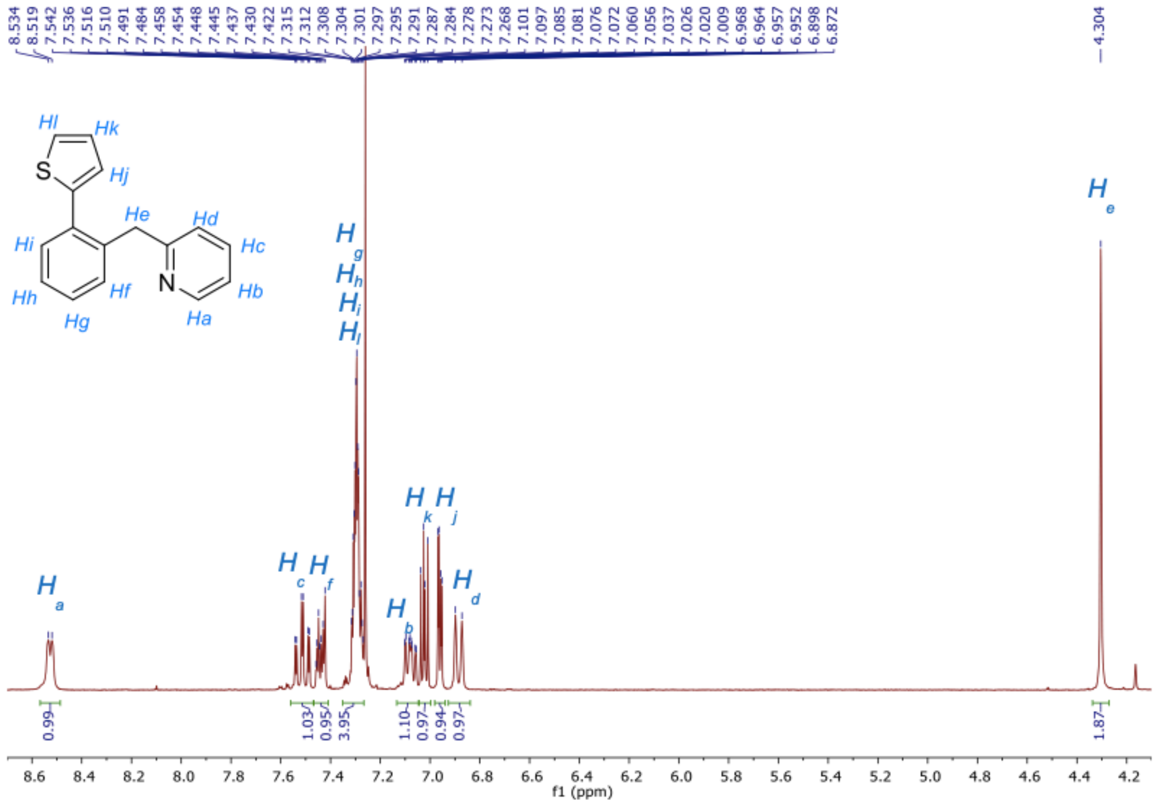

Figure S41. ^{1}H NMR spectrum of **P5** in $CDCl_3$

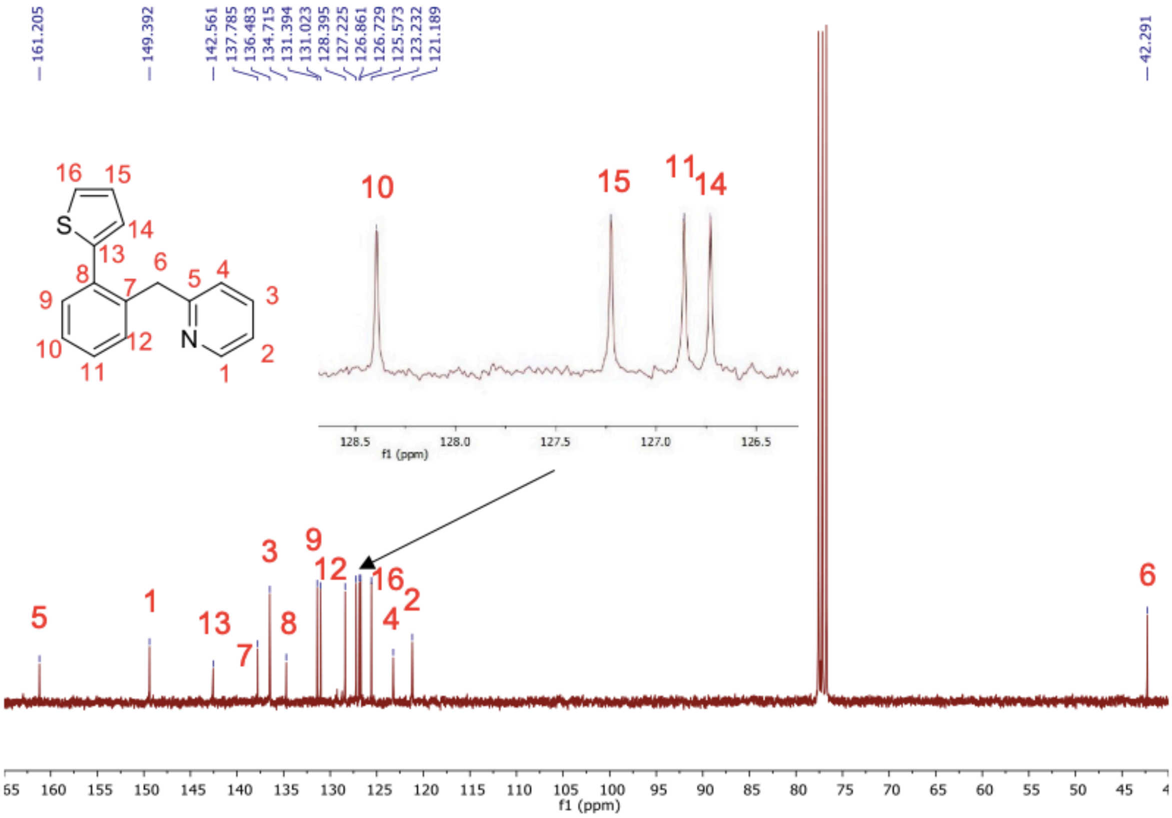

Figure S42. ^{13}C NMR spectrum of **P5** in $CDCl_3$.

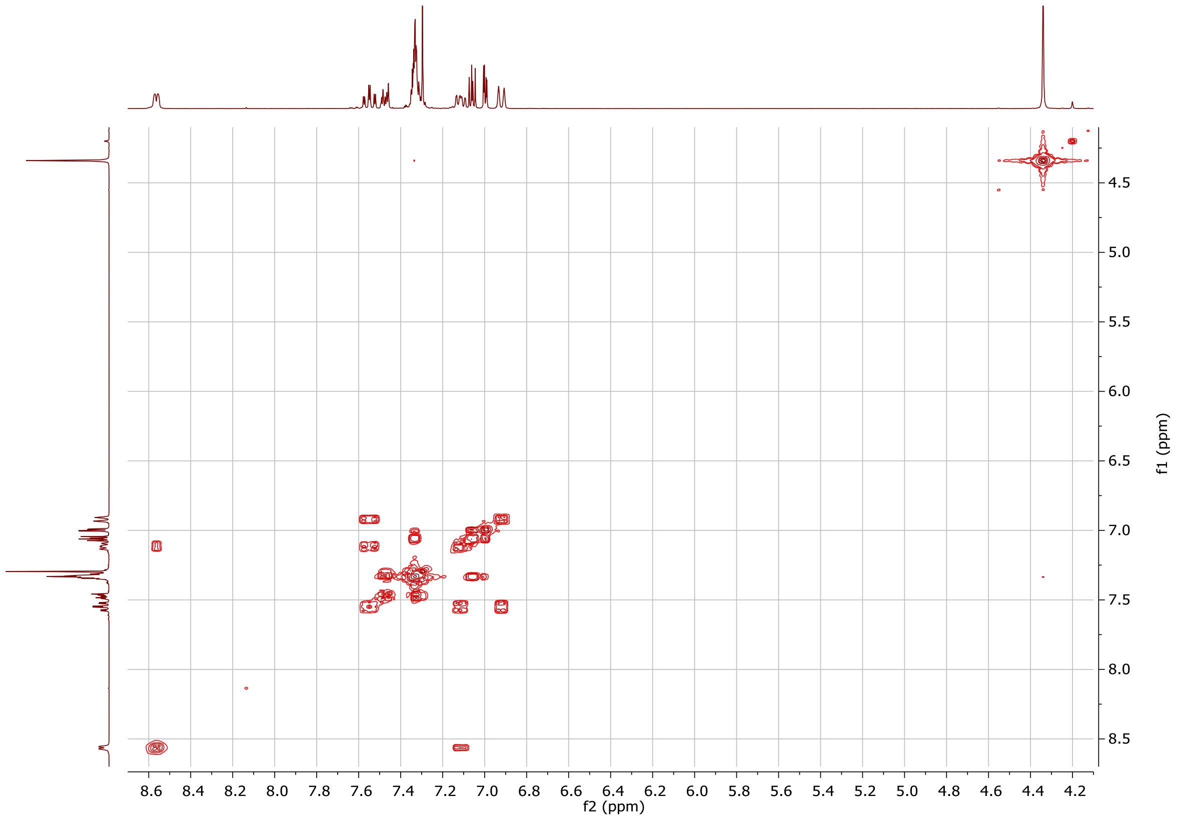

Figure S43. ^1H-^1H COSY NMR of **P5** in $CDCl_3$.

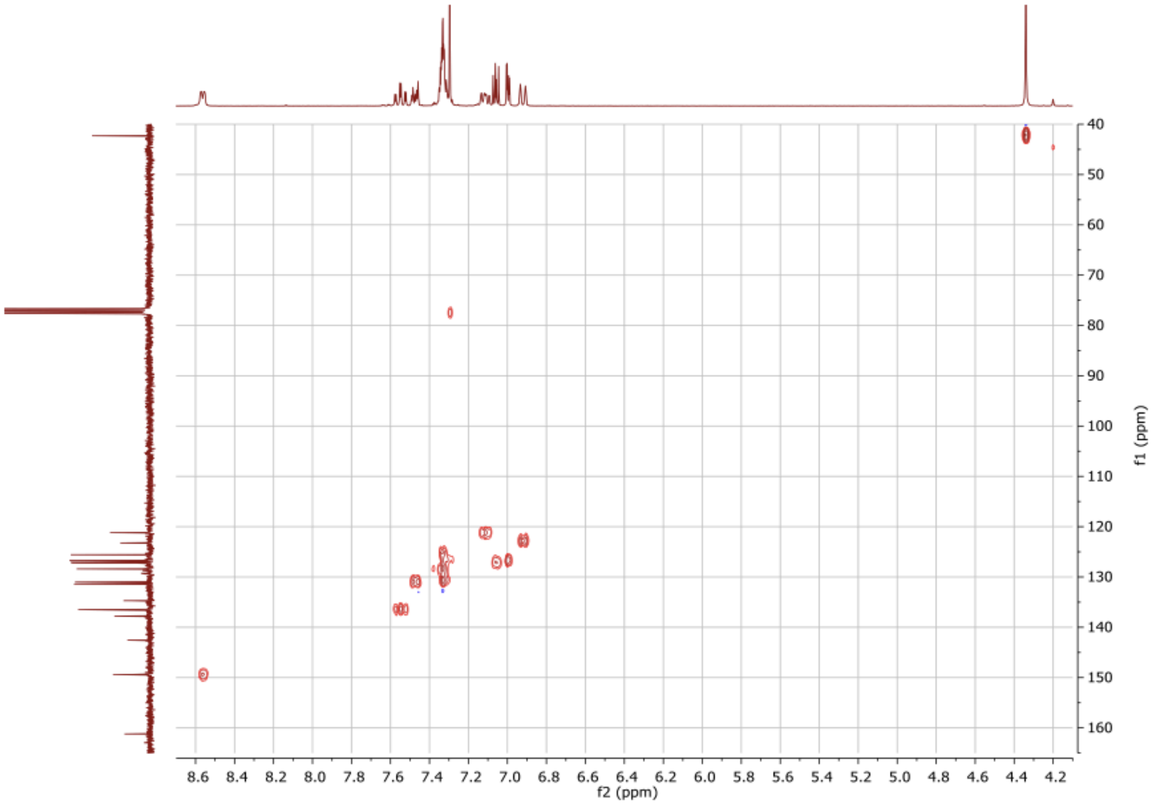

Figure S44. ^1H-^{13}C HSQC NMR of **P5** in $CDCl_3$.

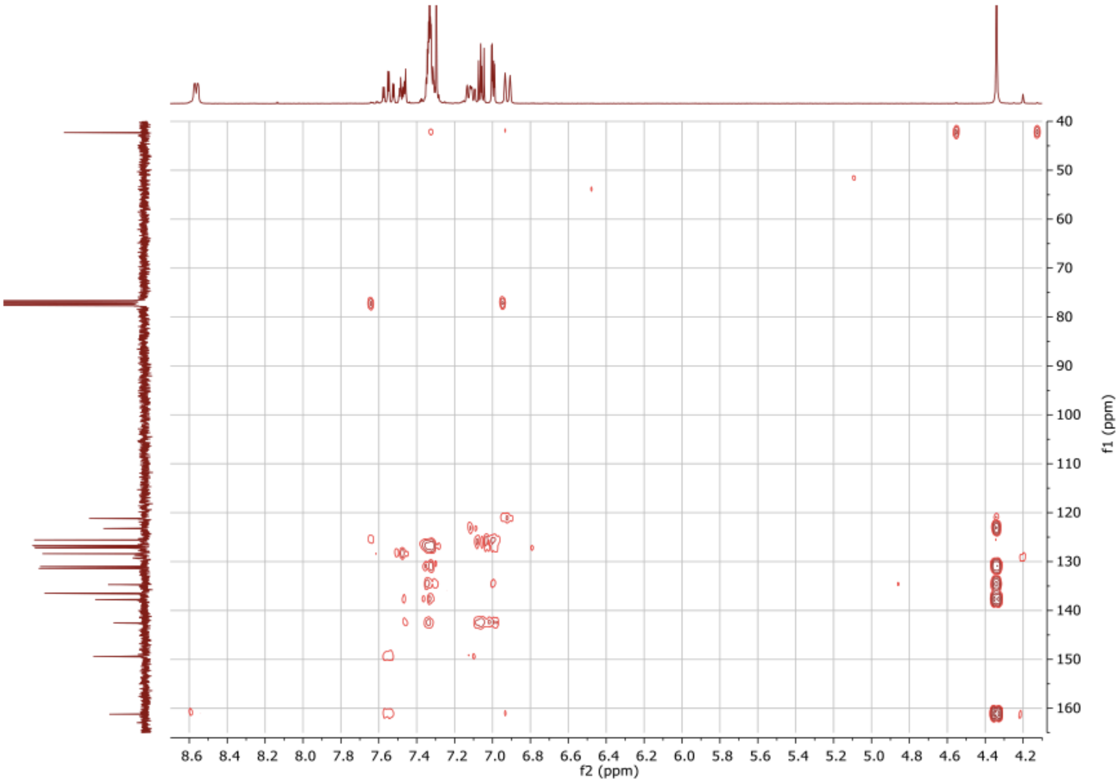

Figure S45. ^{1}H-^{13}C HMBC NMR of **P5** in $CDCl_3$.

XRD data

Compound P3a and P3b, co-crystal (CCDC 2082254)

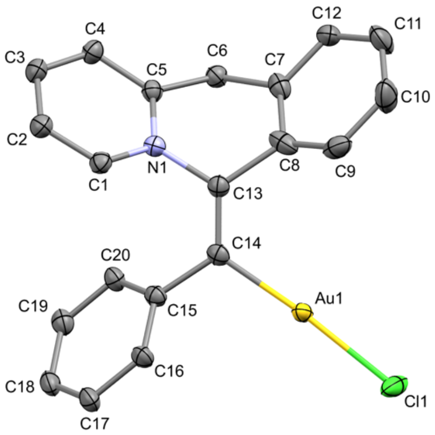

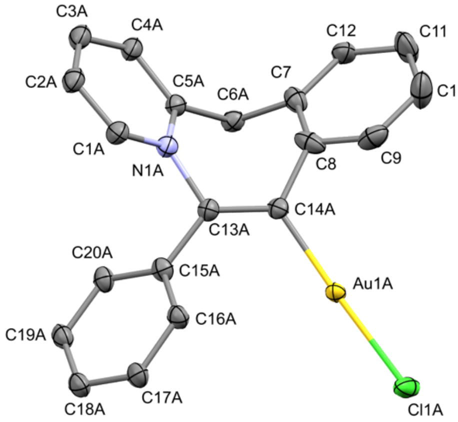

Diffractometer operator C. Jandl
scanspeed 1-10 s per frame
dx 40 mm
1732 frames measured in 10 data sets
phi-scans with delta_phi = 0.5
omega-scans with delta_omega = 0.5
shutterless mode

Crystal data

$C_{20}H_{15}AuClN{\cdot}CHCl_3$	$F(000)$ = 592
M_r = 621.12	
Triclinic, *P*	D_x = 2.011 Mg m^{-3}
Hall symbol: -P 1	Melting point: ? K
a = 8.6641 (8) Å	Mo *K*α radiation, λ = 0.71073 Å
b = 10.4411 (11) Å	Cell parameters from 9933 reflections
c = 12.2469 (12) Å	θ = 2.5–26.4°
α = 75.213 (3)°	μ = 7.70 mm^{-1}
β = 87.664 (3)°	*T* = 100 K
γ = 73.360 (3)°	Fragment, yellow
V = 1025.71 (18) Å^3	0.26 × 0.15 × 0.09 mm
Z = 2	

Data collection

Bruker D8 Venture diffractometer	4027 independent reflections
Radiation source: TXS rotating anode	3971 reflections with $I > 2\sigma(I)$
Helios optic monochromator	R_{int} = 0.018
Detector resolution: 16 pixels mm^{-1}	θ_{max} = 26.0°, θ_{min} = 2.4°
phi– and ω–rotation scans	h = −10 10
Absorption correction: multi-scan *SADABS* 2016/2, Bruker	k = −12 12
T_{min} = 0.543, T_{max} = 0.745	l = −15 15
24327 measured reflections	

Refinement

Refinement on F^2	Secondary atom site location: difference Fourier map
Least-squares matrix: full	Hydrogen site location: inferred from neighbouring sites
$R[F^2 > 2\sigma(F^2)]$ = 0.016	H-atom parameters constrained
$wR(F^2)$ = 0.040	W = $1/[\Sigma^2(FO^2) + (0.0149P)^2 + 1.649P]$ WHERE $P = (FO^2 + 2FC^2)/3$
S = 1.16	$(\Delta/\sigma)_{max}$ = 0.001
4027 reflections	$\Delta\rho_{max}$ = 0.61 e Å^{-3}
398 parameters	$\Delta\rho_{min}$ = −0.77 e Å^{-3}
732 restraints	Extinction correction: none
0 constraints	Extinction coefficient: -
Primary atom site location: iterative	

Compound R1, first polymorph (CCDC 2082255)

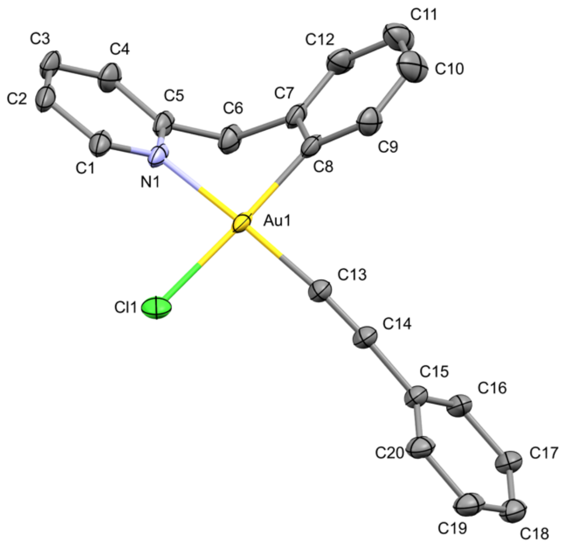

Diffractometer operator C. Jandl
scanspeed 1-10 s per frame
dx 60 mm
5822 frames measured in 20 data sets
phi-scans with delta_phi = 0.5
omega-scans with delta_omega = 0.5
shutterless mode

Crystal data

$C_{20}H_{15}AuClN$	
M_r = 501.75	D_x = 1.972 Mg m^{-3}
Monoclinic, $P2_1/n$	Melting point: ? K
Hall symbol: -P 2yn	Mo *K*α radiation, λ = 0.71073 Å
a = 9.9876 (15) Å	Cell parameters from 9258 reflections
b = 15.304 (2) Å	θ = 2.7–26.3°
c = 11.2071 (16) Å	μ = 8.86 mm^{-1}
β = 99.445 (4)°	*T* = 100 K
V = 1689.8 (4) Å^3	Fragment, colourless
Z = 4	0.21 × 0.14 × 0.08 mm
F(000) = 952	

Data collection

Bruker D8 Venture diffractometer	3327 independent reflections
Radiation source: TXS rotating anode	3258 reflections with $I > 2\sigma(I)$
Helios optic monochromator	$R_{int} = 0.026$
Detector resolution: 16 pixels mm^{-1}	$\theta_{max} = 26.0°$, $\theta_{min} = 2.3°$
phi– and ω–rotation scans	$h = -12$ 12
Absorption correction: multi-scan *SADABS* 2016/2, Bruker	$k = -18$ 18
$T_{min} = 0.557$, $T_{max} = 0.745$	$l = -13$ 13
91541 measured reflections	

Refinement

Refinement on F^2	Secondary atom site location: difference Fourier map
Least-squares matrix: full	Hydrogen site location: inferred from neighbouring sites
$R[F^2 > 2\sigma(F^2)] = 0.018$	H-atom parameters constrained
$wR(F^2) = 0.042$	W = $1/[\Sigma^2(F\mathrm{O}^2) + (0.0163P)^2 + 4.4762P]$ WHERE $P = (F\mathrm{O}^2 + 2F\mathrm{C}^2)/3$
$S = 1.12$	$(\Delta/\sigma)_{max} = 0.003$
3327 reflections	$\Delta\rho_{max} = 0.57$ e Å^{-3}
353 parameters	$\Delta\rho_{min} = -1.51$ e Å^{-3}
631 restraints	Extinction correction: none
0 constraints	Extinction coefficient: -
Primary atom site location: iterative	

Compound R1, second polymorph (CCDC 2082253)

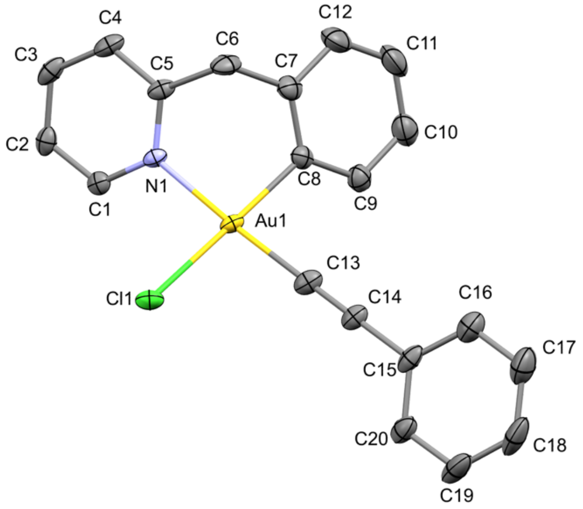

Diffractometer operator C. Jandl
scanspeed 1-10 s per frame
dx 34 mm
2871 frames measured in 13 data sets
phi-scans with delta_phi = 0.5
omega-scans with delta_omega = 0.5
shutterless mode

Crystal data

$C_{20}H_{15}AuClN$	
M_r = 501.75	D_x = 1.936 Mg m^{-3}
Monoclinic, $P2_1/c$	Melting point: ? K
Hall symbol: -P 2ybc	Mo $K\alpha$ radiation, λ = 0.71073 Å
a = 14.2259 (8) Å	Cell parameters from 9890 reflections
b = 14.9029 (8) Å	θ = 2.9–26.4°
c = 8.2468 (5) Å	μ = 8.70 mm^{-1}
β = 100.063 (2)°	T = 100 K
V = 1721.48 (17) Å^3	Shard, colourless
Z = 4	0.21 × 0.10 × 0.03 mm
F(000) = 952	

Data collection

Bruker D8 Venture diffractometer	3399 independent reflections

Radiation source: TXS rotating anode	3121 reflections with $I > 2\sigma(I)$
Helios optic monochromator	R_{int} = 0.037
Detector resolution: 16 pixels mm^{-1}	θ_{max} = 26.0°, θ_{min} = 2.7°
phi– and ω–rotation scans	$h = -17$ 17
Absorption correction: multi-scan *SADABS* 2016/2, Bruker	$k = -18$ 18
T_{min} = 0.614, T_{max} = 0.745	$l = -10$ 10
71803 measured reflections	

Refinement

Refinement on F^2	Secondary atom site location: difference Fourier map
Least-squares matrix: full	Hydrogen site location: inferred from neighbouring sites
$R[F^2 > 2\sigma(F^2)]$ = 0.012	H-atom parameters constrained
$wR(F^2)$ = 0.029	W = $1/[\Sigma^2(FO^2) + (0.0102P)^2 + 1.9834P]$ WHERE $P = (FO^2 + 2FC^2)/3$
S = 1.09	$(\Delta/\sigma)_{max}$ = 0.001
3399 reflections	$\Delta\rho_{max}$ = 0.92 e Å^{-3}
208 parameters	$\Delta\rho_{min}$ = −0.65 e Å^{-3}
0 restraints	Extinction correction: none
0 constraints	Extinction coefficient: -
Primary atom site location: iterative	

Compound R3, (CCDC 2096758)

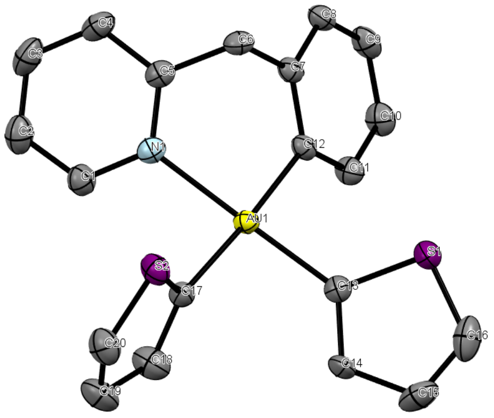

Diffractometer operator M. Rigoulet
scanspeed 1-5 s per frame
dx 42 mm
1685 frames measured in 6 data sets
phi-scans with delta_phi = 0.7
omega-scans with delta_omega = 0.7
shutterless mode

Crystal data

$C_{20}H_{16}AuNS2$

M_r = 531.42	D_x = ? Mg m^{-3}
Monoclinic, $P2_1/n$	Melting point: ? K
Hall symbol: -P 2yn	Mo $K\alpha$ radiation, λ = 0.71073 Å
a = 8.5623 (6) Å	Cell parameters from 9688 reflections
b = 23.4122 (16) Å	θ = 2.9–30.5°
c = 8.6706 (5) Å	μ = 8.75 mm^{-1}
β = 95.930 (2)°	T = 193 (2) K
V = 1728.8 (2) Å^3	Fragment, colourless
Z = 4	0.15 × 0.10 × 0.10 mm
F(000) = 1016	

Data collection

Bruker D8 Venture Photon3 diffractometer	5286 independent reflections
Radiation source: Fine focus sealed tube	5006 reflections with $I > 2\sigma(I)$
Triumph monochromator	R_{int} = 0.032
	θ_{max} = 30.5°, θ_{min} = 2.9°
phi– and ω–rotation scans	h = −12 12
Absorption correction: multi-scan *SADABS* (Bruker-AXS)	k = −33 33
T_{min} = 0.597, T_{max} = 0.746	l = −12 11
71491 measured reflections	

Refinement

Refinement on F^2	Secondary atom site location: difference Fourier map
Least-squares matrix: full	Hydrogen site location: inferred from neighbouring sites
$R[F^2 > 2\sigma(F^2)]$ = 0.024	H-atom parameters constrained
$wR(F^2)$ = 0.045	W = $1/[\Sigma^2(FO^2) + 4.3043P]$ WHERE $P = (FO^2 + 2FC^2)/3$
S = 1.27	$(\Delta/\sigma)_{max}$ = 0.006
5286 reflections	$\Delta\rho_{max}$ = 1.49 e Å^{-3}
256 parameters	$\Delta\rho_{min}$ = −2.50 e Å^{-3}
30 restraints	Extinction correction: none
0 constraints	Extinction coefficient: -
Primary atom site location: dual	
Secondary atom site location: difmap	

DFT data

Table S1. Cartesian coordinates (Å) and Gibbs Free energy values, of the compounds investigated by DFT calculations

1, G°=-1573.944768 au

C	1.498124	0.510093	-0.140469
C	1.359442	1.690756	0.584959
C	2.374530	2.645513	0.488335
C	3.487922	2.418701	-0.312151
C	3.598428	1.236103	-1.033700
C	2.600863	0.265599	-0.945723
C	0.127444	1.944460	1.415702
H	2.281918	3.570083	1.050959
H	4.267590	3.170047	-0.375086
H	4.460820	1.056722	-1.667129
H	2.691663	-0.661699	-1.498865
H	0.190028	2.913600	1.910579
H	0.042604	1.188530	2.207232
C	-1.111331	1.899213	0.574528
C	-2.005810	2.960107	0.497998
C	-3.117276	2.871753	-0.326241
C	-3.322007	1.715653	-1.068419
C	-2.414689	0.681196	-0.951873
N	-1.340713	0.781836	-0.146535
H	-1.814367	3.846909	1.089406
H	-3.814613	3.698891	-0.391982
H	-4.170233	1.603942	-1.730836
H	-2.527059	-0.252954	-1.486899
Au	-0.031671	-0.834162	0.019593
Cl	-1.961986	-2.387599	0.209660
Cl	1.481094	-2.597282	0.241683

R1$_{kin}$, G°=-1421.438418 au

C	2.593141	-0.579792	-0.104195
C	3.251103	0.405167	0.636763
C	4.644530	0.483034	0.595084
C	5.377892	-0.410008	-0.178478
C	4.719923	-1.385671	-0.918859
C	3.329327	-1.475364	-0.875737
C	2.454171	1.391586	1.456778
H	5.152874	1.247119	1.177204
H	6.460439	-0.341041	-0.203740
H	5.285210	-2.082095	-1.530366
H	2.823759	-2.248748	-1.445231
H	3.114111	2.090946	1.971227
H	1.885368	0.864399	2.234510
C	1.493302	2.163839	0.607834
C	1.513636	3.553225	0.536065
C	0.622466	4.221817	-0.287867
C	-0.290129	3.487435	-1.034928
C	-0.284685	2.111489	-0.924507
N	0.591043	1.474615	-0.122762
H	2.237942	4.092956	1.133674
H	0.640234	5.303926	-0.347604
H	-1.003403	3.963305	-1.695129
H	-0.985573	1.482992	-1.458663
Au	0.522638	-0.609868	-0.019149
Cl	0.548368	-2.954157	0.102038
C	-1.544656	-0.558034	0.054152
C	-2.763271	-0.446194	0.067856
C	-4.176149	-0.281115	0.072001
C	-5.034167	-1.357494	0.356692
C	-6.411193	-1.181667	0.358079
C	-6.962278	0.065788	0.075615
C	-6.123545	1.140810	-0.209235
C	-4.745888	0.973112	-0.211417
H	-4.605604	-2.329687	0.576513
H	-7.058728	-2.023520	0.580860

H	-8.038838	0.199505	0.077217
H	-6.545914	2.115637	-0.430643
H	-4.090983	1.810445	-0.432540

R1, G°=-1421.46103 au

C	-0.535392	1.487021	-0.134648
C	-1.535226	2.112168	0.613621
C	-1.651882	3.502120	0.533424
C	-0.793150	4.246107	-0.267043
C	0.194653	3.605826	-1.005772
C	0.332126	2.220837	-0.935930
C	-2.492090	1.311907	1.461427
H	-2.426985	3.997824	1.111162
H	-0.898064	5.324715	-0.313783
H	0.867154	4.178221	-1.636337
H	1.112584	1.723356	-1.499673
H	-3.176028	1.975358	1.991631
H	-1.938663	0.752565	2.227401
C	-3.285229	0.338114	0.641227
C	-4.674886	0.343369	0.614787
C	-5.352832	-0.567225	-0.182071
C	-4.625929	-1.474017	-0.942952
C	-3.246142	-1.442907	-0.871821
N	-2.596014	-0.556349	-0.097108
H	-5.209576	1.064686	1.220696
H	-6.436516	-0.566934	-0.211048
H	-5.111488	-2.198907	-1.583163
H	-2.624974	-2.135975	-1.426187
Au	-0.460852	-0.552187	-0.028590
Cl	-0.468333	-3.031579	0.100887
C	1.498060	-0.472463	0.045467
C	2.712441	-0.378557	0.069447
C	4.127887	-0.247570	0.080309
C	4.721939	1.005597	-0.149983
C	6.102937	1.141697	-0.144626
C	6.916872	0.036112	0.090390
C	6.339659	-1.210276	0.320311
C	4.959215	-1.355508	0.315697
H	4.084814	1.865366	-0.331990
H	6.547001	2.115262	-0.324400
H	7.996159	0.145418	0.094149
H	6.969029	-2.074895	0.503704
H	4.508443	-2.326074	0.493532

R2, G°=-1268.953711 au

C	-1.602970	-1.932108	-0.122852
C	-1.446521	-3.122402	0.598544
C	-2.422693	-4.117355	0.515085
C	-3.552809	-3.936928	-0.275112
C	-3.711384	-2.754873	-0.989795
C	-2.743525	-1.755666	-0.907149
C	-0.217992	-3.336431	1.450849
H	-2.294205	-5.037289	1.079754
H	-4.305432	-4.716732	-0.330113
H	-4.589811	-2.605459	-1.610216
H	-2.883665	-0.829454	-1.456046
H	-0.279464	-4.286288	1.983879
H	-0.155028	-2.548456	2.213235
C	1.045241	-3.308623	0.644810
C	1.926149	-4.384717	0.608767
C	3.076599	-4.312585	-0.161627
C	3.333371	-3.154656	-0.885368
C	2.430612	-2.111630	-0.807842
N	1.312052	-2.191927	-0.064264
H	1.696620	-5.269597	1.190049
H	3.765317	-5.149183	-0.196041
H	4.217965	-3.051087	-1.500398
H	2.585215	-1.174701	-1.329960
Au	-0.063415	-0.543818	-0.020335

C	-1.390315	0.899956	0.028819
C	-2.231170	1.781184	0.045752
C	-3.239249	2.783725	0.057689
C	-4.580924	2.444192	-0.190260
C	-5.568501	3.419116	-0.179467
C	-5.241346	4.748033	0.079531
C	-3.916036	5.096934	0.327344
C	-2.922098	4.128112	0.316391
H	-4.833493	1.407529	-0.389625
H	-6.599341	3.140963	-0.373043
H	-6.015561	5.507898	0.088335
H	-3.655232	6.130588	0.529778
H	-1.889319	4.399177	0.508564
C	1.508792	0.787640	0.042910
C	2.460953	1.556340	0.038948
C	3.589055	2.422293	0.024880
C	4.884195	1.907387	-0.162779
C	5.985188	2.752222	-0.180744
C	5.821254	4.125130	-0.011738
C	4.544174	4.648280	0.175566
C	3.438070	3.809836	0.193770
H	5.011034	0.837203	-0.294283
H	6.977279	2.337580	-0.327408
H	6.683593	4.783187	-0.026099
H	4.409044	5.716884	0.307751
H	2.443187	4.217918	0.338693

P, G°=-825.71712 au

C	-0.676521	1.872952	-0.014722
C	-1.736899	1.191835	0.625562
C	-3.014162	1.748602	0.574953
C	-3.259919	2.948563	-0.081235
C	-2.213299	3.622295	-0.706754
C	-0.934988	3.090506	-0.672076
C	-1.522220	-0.120734	1.337708
H	-3.831033	1.227170	1.067190
H	-4.263715	3.359509	-0.100884
H	-2.394149	4.561193	-1.218792
H	-0.112412	3.607292	-1.154838
C	-1.991899	-1.293443	0.511956
C	-3.205842	-1.925684	0.786303
C	-3.615646	-2.982304	-0.016283
C	-2.799489	-3.376180	-1.068993
C	-1.607077	-2.688336	-1.264149
N	-1.199114	-1.669590	-0.503023
H	-3.814236	-1.592367	1.621165
H	-4.554259	-3.490586	0.179040
H	-3.074482	-4.196359	-1.722184
H	-0.940100	-2.972174	-2.076243
C	0.639975	1.349370	0.000778
H	-0.460757	-0.250898	1.564195
H	-2.066400	-0.108312	2.286487
C	1.764060	0.890227	0.011529
C	3.046136	0.289106	0.038156
C	3.180803	-1.051071	0.444528
C	4.430361	-1.652151	0.475104
C	5.564946	-0.934287	0.103082
C	5.442522	0.392513	-0.302748
C	4.197153	1.003466	-0.336828
H	2.292562	-1.606712	0.728164
H	4.520477	-2.686482	0.789866
H	6.540742	-1.407390	0.128683
H	6.323369	0.954892	-0.594123
H	4.100223	2.036894	-0.651786

1+PhCC$^-$, G°=-1881.788221 au

C	0.277103	-0.967166	0.967408
C	0.618901	-0.180154	2.064620
C	1.791378	-0.490687	2.759637

C	2.586684	-1.560042	2.366632
C	2.221927	-2.331953	1.269054
C	1.056281	-2.040460	0.564116
C	-0.225471	1.008302	2.454292
H	2.072950	0.118223	3.614627
H	3.496247	-1.785331	2.913680
H	2.846895	-3.159103	0.946999
H	0.778236	-2.626146	-0.304636
H	0.179130	1.495825	3.341905
H	-1.245112	0.683139	2.701067
C	-0.297914	1.994913	1.329146
C	0.134729	3.311400	1.443362
C	0.082310	4.151248	0.339828
C	-0.396632	3.659938	-0.868174
C	-0.814061	2.342911	-0.934039
N	-0.767445	1.547880	0.148002
H	0.511967	3.659270	2.397330
H	0.420245	5.178269	0.420755
H	-0.443447	4.277648	-1.755680
H	-1.164799	1.871624	-1.845207
Au	-1.417512	-0.434087	-0.039182
Cl	-3.548991	0.360496	-1.027849
Cl	-2.162869	-2.648075	-0.022801
C	-0.015658	-0.294328	-2.595719
C	1.161357	-0.173752	-2.202529
C	2.484415	-0.054363	-1.694086
C	2.788942	0.873844	-0.677452
C	4.067823	0.968766	-0.147692
C	5.089679	0.144310	-0.615513
C	4.811284	-0.777766	-1.622832
C	3.531948	-0.878045	-2.153451
H	1.996878	1.515622	-0.302288
H	4.268748	1.688395	0.641129
H	6.089489	0.219003	-0.200351
H	5.598339	-1.426626	-1.996004
H	3.321254	-1.604697	-2.932575

R1+Cl$^-$, G°=-1881.850213 au

C	-0.479581	1.567900	-0.232191
C	-1.498128	2.155096	0.522416
C	-1.588397	3.550154	0.539399
C	-0.691247	4.336491	-0.172905
C	0.312364	3.734202	-0.922458
C	0.424783	2.345782	-0.948851
C	-2.489667	1.318533	1.286468
H	-2.376543	4.015548	1.125023
H	-0.778910	5.417462	-0.143812
H	1.015523	4.338386	-1.486743
H	1.216820	1.877043	-1.520806
H	-3.182379	1.958359	1.834747
H	-1.953628	0.697257	2.023143
C	-3.267028	0.401392	0.396732
C	-4.658073	0.407192	0.354655
C	-5.328632	-0.454876	-0.498409
C	-4.593323	-1.316425	-1.303574
C	-3.215078	-1.290953	-1.214514
N	-2.570298	-0.453649	-0.381323
H	-5.198029	1.094531	0.994643
H	-6.412109	-0.451620	-0.538839
H	-5.071617	-2.002123	-1.990883
H	-2.588148	-1.952156	-1.800408
Au	-0.430828	-0.476050	-0.290194
Cl	-0.444435	-2.961244	-0.499025
Cl	-0.517879	-0.982879	3.147121
C	1.531330	-0.424715	-0.225667
C	2.745885	-0.344218	-0.171696
C	4.160868	-0.219588	-0.116862
C	4.760962	1.049398	-0.198490
C	6.141255	1.181737	-0.142790

C	6.950257	0.056320	-0.004518
C	6.367660	-1.205965	0.077408
C	4.987814	-1.347245	0.021868
H	4.128198	1.925146	-0.303158
H	6.588609	2.168373	-0.206066
H	8.028980	0.162661	0.040141
H	6.992463	-2.086444	0.186453
H	4.533481	-2.330147	0.087395

R1+PhCC⁻, G°=-1729.301339 au

C	-0.054457	1.163835	-0.614995
C	0.826605	1.466096	-1.654088
C	1.133450	2.808495	-1.897820
C	0.567669	3.821389	-1.133968
C	-0.313975	3.501002	-0.106372
C	-0.631243	2.169715	0.152806
C	1.468803	0.377334	-2.473836
H	1.826549	3.050196	-2.699407
H	0.816096	4.857420	-1.339258
H	-0.758479	4.284092	0.499704
H	-1.314061	1.919821	0.956362
H	2.090978	0.809580	-3.258833
H	0.691388	-0.216718	-2.973439
C	2.298687	-0.550079	-1.636190
C	3.655603	-0.751270	-1.868200
C	4.364323	-1.635299	-1.069045
C	3.701135	-2.306945	-0.049679
C	2.355000	-2.058846	0.144248
N	1.675859	-1.199794	-0.634907
H	4.138208	-0.211204	-2.674147
H	5.423894	-1.794971	-1.236834
H	4.215027	-3.002905	0.601188
H	1.783699	-2.526702	0.935985
Au	-0.394001	-0.815351	-0.231409
Cl	-0.778873	-3.277007	0.071626
C	-2.306430	-0.422403	-0.005326
C	0.675372	-0.426143	2.581039
C	1.755714	0.130229	2.299027
C	2.982805	0.726921	1.896691
C	3.001191	1.901674	1.115064
C	4.196132	2.456175	0.676470
C	5.414808	1.865583	1.007133
C	5.420183	0.712900	1.790916
C	4.227887	0.152007	2.227901
H	2.056258	2.369130	0.857388
H	4.175521	3.358406	0.070845
H	6.348033	2.300560	0.664039
H	6.362886	0.244592	2.059571
H	4.240140	-0.753800	2.827595
C	-3.486248	-0.141627	0.118579
C	-4.854936	0.210266	0.270539
C	-5.242660	1.562023	0.269303
C	-6.577829	1.913595	0.410031
C	-7.552809	0.929349	0.554513
C	-7.181461	-0.412939	0.558846
C	-5.848089	-0.772985	0.419107
H	-4.481390	2.327473	0.155648
H	-6.859732	2.961549	0.406704
H	-8.595877	1.206932	0.663712
H	-7.935827	-1.184804	0.672103
H	-5.558782	-1.818566	0.422916

R2+Cl⁻, G°=-1729.34105 au

C	-1.541925	-1.974640	-0.355710
C	-1.316796	-3.204462	0.277557
C	-2.251069	-4.234335	0.139361
C	-3.403257	-4.055263	-0.617938
C	-3.627954	-2.836485	-1.248096
C	-2.704105	-1.802579	-1.110911

C	-0.075881	-3.423560	1.106382
H	-2.070238	-5.183585	0.637629
H	-4.120317	-4.864339	-0.714123
H	-4.523148	-2.685463	-1.843975
H	-2.896157	-0.849335	-1.593768
H	-0.085750	-4.419313	1.553034
H	-0.053636	-2.691106	1.930225
C	1.187030	-3.256597	0.324045
C	2.144538	-4.265329	0.242186
C	3.302793	-4.067979	-0.491682
C	3.492473	-2.852080	-1.137809
C	2.518055	-1.880984	-1.017540
N	1.392504	-2.080009	-0.305631
H	1.963846	-5.198966	0.761340
H	4.048423	-4.852309	-0.559847
H	4.379387	-2.649728	-1.724474
H	2.618080	-0.904957	-1.477357
Au	-0.075236	-0.512642	-0.195831
C	-1.467908	0.868838	-0.134079
C	1.423394	0.906161	-0.134834
Cl	0.207677	-0.655931	3.366628
C	-2.333325	1.725902	-0.106042
C	-3.363115	2.705713	-0.079143
C	-4.695572	2.348847	-0.351450
C	-5.701728	3.304533	-0.326364
C	-5.403302	4.632189	-0.029066
C	-4.087535	4.998504	0.243482
C	-3.075478	4.048916	0.218770
H	-4.926621	1.313258	-0.580125
H	-6.724976	3.011942	-0.538581
H	-6.191914	5.376962	-0.008922
H	-3.848322	6.030938	0.476939
H	-2.050487	4.333715	0.431580
C	2.347713	1.708309	-0.144859
C	3.455447	2.599800	-0.166901
C	4.760721	2.109219	-0.352527
C	5.845453	2.974464	-0.378561
C	5.656004	4.345384	-0.219797
C	4.369178	4.844812	-0.034402
C	3.279100	3.985587	-0.008128
H	4.908131	1.040397	-0.474073
H	6.845138	2.577226	-0.522553
H	6.505680	5.019651	-0.239647
H	4.213703	5.911544	0.091134
H	2.277109	4.375756	0.137251

R2+PhCC^{-}, G°=-1576.786982 au

C	1.912647	-2.130095	-0.136114
C	1.726024	-3.217288	-1.000174
C	2.454569	-4.393380	-0.806286
C	3.371469	-4.497176	0.233641
C	3.560846	-3.419038	1.091597
C	2.838297	-2.242961	0.902422
C	0.729574	-3.130385	-2.131962
H	2.301322	-5.230896	-1.482457
H	3.933357	-5.415295	0.372377
H	4.271580	-3.490263	1.909565
H	2.996809	-1.404105	1.573373
H	0.744755	-4.044273	-2.728207
H	1.006784	-2.307263	-2.804660
C	-0.667597	-2.882179	-1.649905
C	-1.717711	-3.751693	-1.927180
C	-2.992280	-3.469889	-1.456426
C	-3.200090	-2.309721	-0.721179
C	-2.122310	-1.477133	-0.479420
N	-0.887848	-1.766862	-0.924778
H	-1.521193	-4.642255	-2.512228
H	-3.813243	-4.146868	-1.665705
H	-4.175916	-2.041951	-0.333889

H	-2.213779	-0.554142	0.077728
Au	0.736448	-0.443724	-0.445170
C	2.296765	0.696858	-0.085679
C	-0.495602	1.170557	-0.810013
C	-0.486173	-0.427606	2.521063
C	3.265914	1.391789	0.166048
C	4.410534	2.172086	0.485577
C	5.548191	1.568276	1.050253
C	6.666120	2.326418	1.369050
C	6.676410	3.698955	1.131614
C	5.556650	4.309321	0.571856
C	4.434168	3.558015	0.251598
H	5.538573	0.498718	1.235310
H	7.534599	1.844041	1.805862
H	7.551441	4.289345	1.382168
H	5.557191	5.378395	0.385187
H	3.561091	4.034067	-0.182171
C	-1.326275	2.051076	-0.992538
C	-2.339642	3.030849	-1.180161
C	-3.687388	2.644002	-1.301175
C	-4.685759	3.593966	-1.465803
C	-4.366288	4.949031	-1.518436
C	-3.036331	5.346071	-1.403801
C	-2.032590	4.401683	-1.235801
H	-3.936693	1.587569	-1.255933
H	-5.719933	3.275178	-1.552216
H	-5.148376	5.689916	-1.646552
H	-2.779300	6.399811	-1.442807
H	-0.997618	4.713441	-1.141620
C	-1.731811	-0.381063	2.567913
C	-3.152894	-0.328955	2.527634
C	-3.954014	-1.317406	3.136437
C	-5.338787	-1.283603	3.037938
C	-5.975291	-0.265927	2.329099
C	-5.203450	0.723536	1.722144
C	-3.818658	0.696617	1.821342
H	-3.467036	-2.119503	3.683121
H	-5.928262	-2.060806	3.515802
H	-7.057414	-0.244135	2.251920
H	-5.684403	1.523978	1.166390
H	-3.222226	1.467892	1.340443

I1, G°=-1729.345749 au

C	0.240830	1.478508	-0.551421
C	0.196231	2.104340	0.698944
C	0.073419	3.498153	0.725587
C	0.002218	4.246296	-0.444137
C	0.052710	3.603370	-1.674867
C	0.169834	2.215151	-1.727817
C	0.265010	1.322296	1.986032
H	0.036209	3.996464	1.691476
H	-0.090802	5.326141	-0.392657
H	-0.000174	4.172942	-2.597381
H	0.202082	1.715196	-2.690325
H	0.390451	2.022752	2.819532
H	1.141530	0.666566	1.980043
C	-0.950329	0.461231	2.235900
C	-2.230943	1.020041	2.250599
C	-3.325761	0.196834	2.457213
C	-3.112147	-1.165478	2.640099
C	-1.805510	-1.631057	2.614658
N	-0.736290	-0.848566	2.424135
H	-2.356964	2.085982	2.082399
H	-4.332662	0.604051	2.456828
H	-3.938733	-1.851462	2.788979
H	-1.598195	-2.691373	2.749098
Au	0.383851	-0.559324	-0.679553
Cl	0.526171	-3.074038	-0.841640
C	2.374560	-0.415436	-0.382908

C	-1.618145	-0.581306	-0.906213
C	3.574859	-0.300915	-0.176491
C	4.973206	-0.190137	0.056671
C	5.561203	1.058807	0.326883
C	6.926388	1.165075	0.553831
C	7.736769	0.032872	0.516648
C	7.168395	-1.210595	0.250405
C	5.803917	-1.324792	0.023062
H	4.930717	1.941615	0.355582
H	7.361282	2.137709	0.760716
H	8.803630	0.118840	0.693980
H	7.792750	-2.097828	0.219792
H	5.362155	-2.294042	-0.183929
C	-2.835570	-0.474267	-0.887935
C	-4.241562	-0.303657	-0.786196
C	-4.810401	0.976799	-0.912578
C	-6.175916	1.164976	-0.748867
C	-7.005031	0.084178	-0.456758
C	-6.455733	-1.190406	-0.334173
C	-5.091283	-1.385805	-0.498388
H	-4.160781	1.820115	-1.127887
H	-6.596031	2.161094	-0.845883
H	-8.071563	0.233987	-0.325726
H	-7.095440	-2.037261	-0.106710
H	-4.661902	-2.376774	-0.391038

I1', G°=-1729.387797 au

C	1.220515	2.330486	-0.821768
C	1.776180	3.072433	0.247749
C	1.585435	4.452479	0.260450
C	0.866979	5.096167	-0.742346
C	0.310937	4.358088	-1.783301
C	0.481683	2.982597	-1.818383
C	2.523519	2.381587	1.356411
H	2.004744	5.029396	1.079893
H	0.733469	6.172102	-0.704861
H	-0.257635	4.852478	-2.563261
H	0.046618	2.392011	-2.617973
H	2.884926	3.136083	2.064677
H	3.414953	1.874783	0.970788
C	1.708784	1.343816	2.094564
C	0.360878	1.558201	2.388381
C	-0.361926	0.552988	3.014786
C	0.283353	-0.636252	3.331699
C	1.634015	-0.747724	3.024514
N	2.345642	0.211178	2.424236
H	-0.115368	2.491705	2.103241
H	-1.416181	0.688145	3.234851
H	-0.244168	-1.460303	3.798441
H	2.174924	-1.662507	3.262249
Au	-0.119785	-0.539444	-0.567216
Cl	-0.222127	-3.357863	0.166422
C	1.424700	0.912546	-0.861598
C	-2.136429	-0.458364	-0.449439
C	1.982193	-0.218486	-0.759393
C	3.183074	-1.008632	-0.761611
C	4.413743	-0.363918	-0.981869
C	5.595355	-1.089219	-0.966747
C	5.570874	-2.463432	-0.733286
C	4.355926	-3.107265	-0.516136
C	3.166089	-2.389636	-0.529795
H	4.427281	0.705833	-1.168509
H	6.539587	-0.582589	-1.137644
H	6.497180	-3.028378	-0.721716
H	4.334578	-4.176804	-0.333461
H	2.211550	-2.878718	-0.353336
C	-3.359251	-0.411827	-0.349083
C	-4.773864	-0.357122	-0.224468
C	-5.380002	-0.123261	1.024408

C	-6.761548	-0.067889	1.145416
C	-7.575202	-0.243948	0.028521
C	-6.991067	-0.476454	-1.214456
C	-5.610109	-0.532531	-1.343146
H	-4.748617	0.012760	1.896705
H	-7.207119	0.113026	2.118580
H	-8.654868	-0.200853	0.126013
H	-7.616515	-0.615290	-2.090736
H	-5.158650	-0.713974	-2.313124

P1, G°=-1729.410097 au

C	-1.565816	2.205552	-0.840372
C	-2.038227	2.715406	0.393022
C	-3.412088	2.831196	0.584454
C	-4.316704	2.464046	-0.408541
C	-3.849638	1.964501	-1.620118
C	-2.485669	1.834497	-1.833919
C	-1.063379	3.097916	1.472666
H	-3.776971	3.218176	1.532235
H	-5.383188	2.561003	-0.232912
H	-4.547907	1.665261	-2.394368
H	-2.111878	1.434633	-2.771035
H	-1.597533	3.643119	2.259719
H	-0.301084	3.777919	1.079665
C	-0.342031	1.921209	2.089671
C	-1.040839	0.771418	2.461625
C	-0.350770	-0.285197	3.034996
C	1.022475	-0.167321	3.213741
C	1.633661	1.012838	2.810640
N	0.980899	2.045706	2.267205
H	-2.110110	0.709811	2.282412
H	-0.872584	-1.194028	3.318413
H	1.610856	-0.974133	3.636455
H	2.710571	1.136100	2.919987
Au	1.273448	-1.730782	-0.049414
Cl	3.625242	-1.845235	0.366886
C	-0.173265	2.068755	-1.056033
C	-0.653927	-1.686915	-0.322860
C	1.029770	1.957539	-1.181159
C	2.434741	1.833493	-1.309436
C	3.280758	2.314083	-0.293297
C	4.657719	2.197955	-0.418292
C	5.215678	1.605850	-1.549432
C	4.385706	1.122666	-2.558587
C	3.007147	1.231775	-2.444083
H	2.831971	2.759392	0.588930
H	5.301042	2.569980	0.372638
H	6.292981	1.516965	-1.642280
H	4.815410	0.655202	-3.438446
H	2.358143	0.852162	-3.226344
C	-1.876669	-1.627969	-0.409024
C	-3.292095	-1.538814	-0.469908
C	-3.977165	-1.558500	-1.699037
C	-5.360834	-1.460995	-1.744048
C	-6.099093	-1.339204	-0.568974
C	-5.436685	-1.320377	0.656692
C	-4.053085	-1.419970	0.708939
H	-3.404683	-1.644081	-2.617399
H	-5.867662	-1.476520	-2.704094
H	-7.180686	-1.262250	-0.607570
H	-6.002680	-1.228771	1.578591
H	-3.539848	-1.411733	1.665661

I2, G°=-1576.834637 au

C	-0.279208	-2.193274	-0.808850
C	-0.570306	-3.038168	0.276233
C	-0.598078	-4.422086	0.074997
C	-0.352624	-4.978168	-1.176495
C	-0.065563	-4.142017	-2.248731

C	-0.026206	-2.761106	-2.058379
C	-0.830175	-2.478014	1.653831
H	-0.819583	-5.070519	0.920320
H	-0.383836	-6.055009	-1.310059
H	0.131274	-4.558993	-3.232206
H	0.209876	-2.119516	-2.903488
H	-1.181715	-3.284290	2.308787
H	-1.622583	-1.724351	1.611606
C	0.383324	-1.833134	2.277014
C	1.572540	-2.552500	2.431110
C	2.672875	-1.930705	2.999597
C	2.561465	-0.599591	3.390722
C	1.342205	0.035582	3.200551
N	0.266764	-0.555032	2.665291
H	1.623582	-3.585195	2.098222
H	3.606274	-2.469735	3.128811
H	3.397655	-0.066993	3.829558
H	1.216077	1.077568	3.490280
Au	-0.226901	-0.116546	-0.592667
C	-2.241374	-0.153535	-0.454345
C	-0.172488	1.937536	-0.373377
C	1.785287	-0.204600	-0.730646
C	-3.453516	-0.250965	-0.327071
C	-4.854256	-0.413340	-0.148191
C	-5.359091	-1.576641	0.462327
C	-6.724104	-1.745573	0.647335
C	-7.618406	-0.763948	0.226162
C	-7.133485	0.391631	-0.381606
C	-5.768983	0.569153	-0.566707
H	-4.661255	-2.342491	0.787809
H	-7.093294	-2.649266	1.121847
H	-8.685182	-0.898741	0.370597
H	-7.823568	1.161258	-0.712586
H	-5.393500	1.471419	-1.038258
C	-0.130852	3.151895	-0.221979
C	-0.077654	4.561803	-0.041186
C	0.859049	5.142445	0.833515
C	0.908984	6.518503	1.009308
C	0.031310	7.350207	0.317919
C	-0.900761	6.790332	-0.552594
C	-0.958439	5.414887	-0.730971
H	1.544823	4.496427	1.372271
H	1.638901	6.945284	1.689993
H	0.073287	8.425449	0.456306
H	-1.589232	7.430070	-1.095749
H	-1.687027	4.981582	-1.408473
C	3.004694	-0.287542	-0.768724
C	4.421875	-0.399215	-0.786662
C	5.056272	-1.546175	-0.273986
C	6.440023	-1.655100	-0.281348
C	7.224782	-0.628350	-0.801383
C	6.611172	0.511615	-1.315091
C	5.227857	0.628912	-1.308038
H	4.444865	-2.347676	0.128520
H	6.909810	-2.547615	0.119811
H	8.306129	-0.716322	-0.806885
H	7.214989	1.316166	-1.722813
H	4.752225	1.519312	-1.706133

I2’, G°=-1576.865066 au

C	1.719022	-2.628247	-0.722462
C	1.806864	-3.616805	0.287609
C	2.980213	-4.362347	0.392135
C	4.052434	-4.160621	-0.471613
C	3.969361	-3.177011	-1.452806
C	2.818708	-2.410023	-1.565017
C	0.675215	-3.826022	1.255871
H	3.051413	-5.114609	1.173490
H	4.951926	-4.758571	-0.369116

H	4.803824	-3.000572	-2.123571
H	2.751490	-1.627296	-2.314516
H	0.939365	-4.638010	1.944294
H	-0.231316	-4.159130	0.736963
C	0.289397	-2.604244	2.058363
C	1.228156	-1.632960	2.413294
C	0.807695	-0.506638	3.104921
C	-0.538293	-0.383766	3.430258
C	-1.392807	-1.415943	3.063472
N	-1.004940	-2.507048	2.395960
H	2.267289	-1.749288	2.119882
H	1.516662	0.271251	3.371081
H	-0.918161	0.486520	3.953325
H	-2.452330	-1.361944	3.310157
Au	0.050786	0.215320	-0.634448
C	0.530392	-1.825438	-0.842319
C	-1.493604	1.598002	-0.435193
C	1.746685	1.409373	-0.435053
C	-0.751875	-1.696076	-0.848003
C	-2.046856	-2.318846	-0.902890
C	-2.165550	-3.692201	-1.182917
C	-3.411897	-4.302765	-1.200134
C	-4.561797	-3.559429	-0.939445
C	-4.456515	-2.196749	-0.669330
C	-3.213169	-1.578806	-0.656240
H	-1.269751	-4.268646	-1.396442
H	-3.488726	-5.363210	-1.418557
H	-5.534537	-4.040105	-0.951709
H	-5.349718	-1.612588	-0.469848
H	-3.116166	-0.515714	-0.450255
C	-2.483102	2.308866	-0.272899
C	-3.657544	3.086977	-0.081413
C	-4.787017	2.532262	0.551785
C	-5.940101	3.281873	0.738445
C	-6.002063	4.602174	0.297859
C	-4.894526	5.165936	-0.331316
C	-3.736943	4.422546	-0.518515
H	-4.741438	1.503062	0.895690
H	-6.797701	2.832770	1.229803
H	-6.904859	5.185970	0.443750
H	-4.932645	6.194003	-0.678018
H	-2.876062	4.866576	-1.008074
C	2.768072	2.078459	-0.287944
C	3.947938	2.851916	-0.115198
C	4.482066	3.085783	1.166822
C	5.635446	3.840129	1.332624
C	6.289655	4.383394	0.229134
C	5.774603	4.162263	-1.046201
C	4.621689	3.408917	-1.219474
H	3.976054	2.665057	2.030140
H	6.027181	4.006020	2.331506
H	7.190825	4.972790	0.361684
H	6.275378	4.580852	-1.913813
H	4.224749	3.240288	-2.215458

P2, G°=-1576.909704 au

C	0.321310	1.657001	-1.630324
C	-0.417412	2.459113	-0.727894
C	-1.793594	2.568986	-0.907240
C	-2.443117	1.920705	-1.953697
C	-1.713835	1.139467	-2.844471
C	-0.342953	1.006026	-2.682869
C	0.283584	3.170450	0.396505
H	-2.364496	3.179359	-0.212132
H	-3.517718	2.019235	-2.066941
H	-2.213763	0.627637	-3.659882
H	0.234805	0.393090	-3.366869
H	-0.403059	3.902249	0.838765
H	1.140482	3.736385	0.017300

C	0.793268	2.263014	1.492142
C	0.018314	1.201287	1.961393
C	0.506105	0.409303	2.990012
C	1.755100	0.701553	3.525270
C	2.456666	1.776191	2.992972
N	2.001112	2.549429	2.001557
H	-0.946771	0.989690	1.508885
H	-0.076726	-0.429368	3.358063
H	2.183185	0.106433	4.324160
H	3.445637	2.027789	3.374791
Au	-1.536398	-1.528975	0.051480
C	1.722127	1.530313	-1.471115
C	0.417267	-1.945882	0.028157
C	-3.466100	-1.016218	0.091042
C	2.922561	1.467565	-1.296485
C	4.320091	1.414831	-1.074156
C	4.887106	2.120085	0.003302
C	6.254740	2.065596	0.229997
C	7.078318	1.312325	-0.604428
C	6.524286	0.609507	-1.671579
C	5.158559	0.657023	-1.908732
H	4.232350	2.692829	0.653493
H	6.682151	2.612324	1.064633
H	8.147207	1.271319	-0.421992
H	7.160290	0.013993	-2.318640
H	4.723202	0.099429	-2.731664
C	1.639015	-2.085939	0.037423
C	3.053991	-2.192728	0.094407
C	3.801191	-1.292365	0.878458
C	5.182109	-1.390393	0.954700
C	5.858463	-2.382093	0.248043
C	5.136581	-3.274804	-0.540588
C	3.752518	-3.185442	-0.617916
H	3.276209	-0.504707	1.411016
H	5.736898	-0.675592	1.555223
H	6.940444	-2.450021	0.301468
H	5.655156	-4.047420	-1.100043
H	3.193284	-3.885228	-1.230925
C	-4.625793	-0.606297	0.102318
C	-5.948623	-0.090428	0.101989
C	-7.064790	-0.928180	-0.084167
C	-8.352088	-0.408651	-0.087006
C	-8.563561	0.956095	0.095810
C	-7.470178	1.799229	0.281929
C	-6.180178	1.287150	0.285437
H	-6.904927	-1.992069	-0.226847
H	-9.197465	-1.073896	-0.232687
H	-9.570936	1.358817	0.093433
H	-7.624225	2.864154	0.425679
H	-5.329834	1.946251	0.432046

I3, G°=-1421.505053 au

C	-0.397032	2.245423	-0.003709
C	-1.697113	2.176648	0.548919
C	-2.544055	3.272618	0.385007
C	-2.139291	4.413500	-0.297143
C	-0.853279	4.484369	-0.825787
C	0.008922	3.410923	-0.679435
C	-2.186081	0.987711	1.348888
H	-3.539726	3.233097	0.817605
H	-2.822104	5.249080	-0.405650
H	-0.523947	5.373215	-1.352597
H	1.008128	3.449215	-1.100223
H	-2.886019	1.355462	2.103450
H	-1.355626	0.524741	1.889615
C	-2.892107	-0.075569	0.543505
C	-4.269308	-0.025826	0.338501
C	-4.894021	-1.029106	-0.387531
C	-4.127978	-2.072470	-0.893067

C	-2.763722	-2.061305	-0.659187
N	-2.158865	-1.088293	0.038890
H	-4.840764	0.794057	0.759203
H	-5.965935	-1.001316	-0.549158
H	-4.573689	-2.881976	-1.457542
H	-2.109850	-2.848968	-1.021870
Au	0.109424	-0.866621	0.053963
Cl	0.445339	-3.333470	-0.040904
C	0.527723	1.153593	0.068199
C	1.648314	0.538637	0.010015
C	3.076476	0.443135	-0.000114
C	3.851294	1.616797	-0.011984
C	5.235320	1.535643	-0.024139
C	5.866845	0.293044	-0.023861
C	5.106033	-0.873375	-0.011200
C	3.720317	-0.803266	-0.000428
H	3.357085	2.582728	-0.007503
H	5.825987	2.445512	-0.032759
H	6.950002	0.235354	-0.033068
H	5.595229	-1.841487	-0.010380
H	3.114467	-1.705324	0.008217

I3’, G°=-1421.499852 au

C	1.831891	-1.269238	-0.801936
C	2.355181	-1.886324	0.358647
C	3.694261	-2.267066	0.347982
C	4.502758	-2.043852	-0.762700
C	3.982745	-1.423411	-1.894666
C	2.652576	-1.033454	-1.913468
C	1.489910	-2.100045	1.570504
H	4.109124	-2.742350	1.231908
H	5.543278	-2.349493	-0.740480
H	4.611570	-1.242747	-2.759041
H	2.230293	-0.546952	-2.786150
H	2.072050	-2.628204	2.333801
H	0.640154	-2.748038	1.330492
C	0.929296	-0.824071	2.156251
C	1.712953	0.327896	2.256486
C	1.143987	1.489164	2.759297
C	-0.189511	1.465005	3.151700
C	-0.882627	0.266424	3.036129
N	-0.350763	-0.860797	2.551512
H	2.747413	0.311899	1.926189
H	1.727118	2.401472	2.832225
H	-0.682174	2.351235	3.535138
H	-1.926412	0.206030	3.339665
Au	-0.149215	1.192826	-0.685507
Cl	-0.072116	3.523647	-0.595192
C	0.453904	-0.890448	-0.819763
C	-0.791546	-0.823560	-0.679939
C	-2.135468	-1.302197	-0.553994
C	-2.358516	-2.689033	-0.570588
C	-3.645604	-3.183533	-0.424255
C	-4.719632	-2.310818	-0.261999
C	-4.503204	-0.935665	-0.244063
C	-3.218784	-0.430118	-0.387028
H	-1.518498	-3.363562	-0.698696
H	-3.812355	-4.255123	-0.436950
H	-5.724262	-2.703498	-0.148413
H	-5.337253	-0.254675	-0.115540
H	-3.038124	0.640429	-0.367033

P3a, G°=-1421.516245 au

C	-0.567526	1.958748	-0.222582
C	-1.404912	2.783773	0.539955
C	-1.177408	4.154175	0.597156
C	-0.111157	4.709889	-0.102338
C	0.719178	3.895444	-0.868648
C	0.490890	2.526642	-0.936593

C	-2.506858	2.098180	1.298884
H	-1.829801	4.782436	1.195803
H	0.066919	5.778833	-0.056289
H	1.541116	4.330347	-1.427067
H	1.122895	1.892317	-1.549904
H	-3.255398	2.796230	1.673566
H	-2.080089	1.589590	2.177759
C	-3.160809	1.068179	0.443368
C	-4.528945	0.858921	0.369350
C	-5.042950	-0.116899	-0.469807
C	-4.173529	-0.859245	-1.263806
C	-2.822808	-0.621447	-1.179751
N	-2.330544	0.302388	-0.317020
H	-5.177513	1.473746	0.980460
H	-6.112110	-0.283650	-0.522114
H	-4.533476	-1.606163	-1.958228
H	-2.089916	-1.135112	-1.785221
Au	1.967625	-0.263624	0.038299
Cl	4.345851	-0.020182	0.191623
C	-0.877403	0.521277	-0.223443
C	-0.024750	-0.520860	-0.076802
C	-0.501645	-1.911534	0.081313
C	-0.022094	-2.928762	-0.756323
C	-0.478920	-4.233311	-0.624742
C	-1.397742	-4.559874	0.370198
C	-1.855532	-3.567424	1.231513
C	-1.418695	-2.255979	1.085502
H	0.709566	-2.679242	-1.519932
H	-0.107184	-5.001903	-1.294688
H	-1.743671	-5.582036	0.480715
H	-2.557136	-3.813860	2.022028
H	-1.780510	-1.488233	1.764588

P3b, G°=-1421.515456 au

C	-0.855086	-1.735322	-0.121243
C	-1.964424	-1.998287	0.704978
C	-2.571683	-3.252932	0.694858
C	-2.089420	-4.262270	-0.127336
C	-0.987872	-4.017356	-0.946766
C	-0.368629	-2.778212	-0.926647
C	-2.495982	-0.874297	1.553745
H	-3.423556	-3.435675	1.343488
H	-2.562086	-5.238513	-0.122999
H	-0.603383	-4.801532	-1.590270
H	0.507571	-2.591270	-1.541316
H	-1.686245	-0.394353	2.116703
H	-3.248890	-1.226562	2.258464
C	-3.112042	0.101891	0.619043
C	-4.487627	0.235071	0.461190
C	-5.013642	1.116229	-0.463429
C	-4.142042	1.862026	-1.257030
C	-2.791323	1.707358	-1.090490
N	-2.277793	0.843947	-0.167174
H	-5.130130	-0.364866	1.093413
H	-6.086174	1.225021	-0.569708
H	-4.503009	2.555660	-2.004245
H	-2.060067	2.248885	-1.674416
Au	1.851690	-0.520932	0.016818
Cl	4.236401	-0.676246	0.194530
C	-0.165571	-0.438083	-0.083622
C	-0.816942	0.754686	-0.064414
C	-0.187405	2.091332	-0.004776
C	-0.682598	3.053084	0.886994
C	-0.087901	4.304365	0.983793
C	1.001978	4.624341	0.178766
C	1.487105	3.686806	-0.728184
C	0.896269	2.432597	-0.823325
H	-1.529499	2.809404	1.523131
H	-0.473274	5.029108	1.693125

H	1.464828	5.602684	0.252233
H	2.322754	3.935705	-1.373762
H	1.262417	1.710335	-1.545931

I4, G°=-1269.001824 au

C	-3.200017	-0.518018	-0.084844
C	-3.154068	-1.787750	0.530991
C	-4.225592	-2.660406	0.337439
C	-5.320810	-2.309975	-0.441482
C	-5.367849	-1.054796	-1.042941
C	-4.319679	-0.168247	-0.863502
C	-1.998018	-2.224558	1.398654
H	-4.200628	-3.633193	0.820432
H	-6.138895	-3.010158	-0.571478
H	-6.220516	-0.767208	-1.648344
H	-4.342784	0.809321	-1.333721
H	-2.380840	-2.876126	2.188401
H	-1.537467	-1.360223	1.886000
C	-0.932743	-2.968345	0.632660
C	-0.970220	-4.355837	0.494418
C	0.030055	-4.995567	-0.223528
C	1.050116	-4.235399	-0.784262
C	1.019580	-2.861289	-0.603759
N	0.052570	-2.242733	0.083000
H	-1.772768	-4.921063	0.956225
H	0.018239	-6.074206	-0.337087
H	1.855125	-4.694428	-1.345473
H	1.796595	-2.213445	-1.004206
Au	-0.040933	0.239518	0.093383
C	-2.145134	0.447079	0.036937
C	-1.637717	1.611571	0.001082
C	-1.631691	3.044249	-0.001438
C	-2.860206	3.727684	-0.048890
C	-2.885299	5.114124	-0.058214
C	-1.695792	5.839210	-0.018988
C	-0.476136	5.168926	0.029929
C	-0.440229	3.781785	0.037761
H	-3.785227	3.160986	-0.074248
H	-3.837510	5.632615	-0.094880
H	-1.720763	6.923609	-0.026258
H	0.451526	5.730285	0.060995
H	0.504146	3.247094	0.073813
C	1.982721	0.323590	0.076031
C	3.208349	0.279772	0.025595
C	4.626843	0.212495	-0.034457
C	5.278765	-1.011858	-0.273262
C	6.663772	-1.078405	-0.334312
C	7.433038	0.069577	-0.159094
C	6.802307	1.288478	0.078532
C	5.417642	1.363050	0.140526
H	4.680627	-1.907685	-0.408848
H	7.146765	-2.032575	-0.519673
H	8.515433	0.014589	-0.207119
H	7.393787	2.188040	0.216406
H	4.928960	2.314139	0.325299

I4’, G°=-1269.006988 au

C	-2.248896	-1.876110	-0.809106
C	-2.859799	-2.428184	0.340970
C	-3.142338	-3.791332	0.340668
C	-2.830843	-4.596223	-0.751419
C	-2.219280	-4.046693	-1.874237
C	-1.925862	-2.692065	-1.902639
C	-3.168083	-1.563593	1.532751
H	-3.611298	-4.227791	1.217616
H	-3.061346	-5.655756	-0.722087
H	-1.970582	-4.671519	-2.724563
H	-1.447546	-2.246618	-2.768366
H	-3.699260	-2.164183	2.279544

H	-3.843982	-0.747800	1.255558
C	-1.945579	-0.938673	2.165887
C	-0.777282	-1.679947	2.357235
C	0.332040	-1.059664	2.913334
C	0.242582	0.283191	3.261339
C	-0.966531	0.933857	3.049307
N	-2.045877	0.350782	2.516935
H	-0.741316	-2.723483	2.058547
H	1.255085	-1.610843	3.061354
H	1.086971	0.815141	3.684710
H	-1.076969	1.983982	3.314630
Au	0.172216	0.136755	-0.656296
C	-1.967804	-0.476752	-0.838023
C	-1.920070	0.765076	-0.753757
C	-2.373670	2.121484	-0.680420
C	-3.753337	2.357216	-0.557323
C	-4.228731	3.656425	-0.464166
C	-3.343559	4.731987	-0.492544
C	-1.975626	4.503909	-0.613351
C	-1.488788	3.207157	-0.704207
H	-4.437162	1.515354	-0.535101
H	-5.294892	3.831068	-0.368191
H	-3.720453	5.746400	-0.419157
H	-1.284306	5.339209	-0.634114
H	-0.423035	3.020447	-0.793510
C	2.136602	0.099776	-0.498740
C	3.355482	0.091070	-0.364808
C	4.767273	0.080542	-0.206606
C	5.561165	-0.877217	-0.863150
C	6.939522	-0.883591	-0.700862
C	7.557971	0.063000	0.112372
C	6.784074	1.018456	0.766546
C	5.404832	1.029287	0.613567
H	5.081099	-1.613853	-1.498775
H	7.535552	-1.631316	-1.214028
H	8.635736	0.056097	0.235468
H	7.258245	1.759497	1.401924
H	4.802718	1.772515	1.125662

P4a, G°=-1269.015037 au

C	-1.712064	1.963428	-0.247300
C	-2.505446	2.815476	0.532702
C	-2.246579	4.181201	0.566014
C	-1.191825	4.705703	-0.174160
C	-0.403727	3.864194	-0.955848
C	-0.664294	2.500091	-1.000080
C	-3.594761	2.162224	1.337426
H	-2.865124	4.830583	1.178084
H	-0.989571	5.771037	-0.146958
H	0.409609	4.274631	-1.544744
H	-0.066253	1.842968	-1.623410
H	-4.315748	2.880150	1.728568
H	-3.148091	1.655609	2.207643
C	-4.298433	1.134691	0.519300
C	-5.672376	0.951474	0.499123
C	-6.235791	-0.026568	-0.304985
C	-5.410002	-0.797782	-1.117731
C	-4.052694	-0.583996	-1.087209
N	-3.510971	0.343306	-0.259242
H	-6.285706	1.588143	1.124114
H	-7.309134	-0.173303	-0.315568
H	-5.809137	-1.548553	-1.786194
H	-3.352717	-1.120367	-1.711469
Au	0.856502	-0.317126	-0.052111
C	-2.046329	0.531855	-0.223793
C	-1.200807	-0.518190	-0.106022
C	-1.702035	-1.895159	0.084929
C	-1.278570	-2.930733	-0.761945
C	-1.747631	-4.227106	-0.594349

C	-2.621960	-4.530229	0.447085
C	-3.022993	-3.521306	1.317805
C	-2.575582	-2.217999	1.135322
H	-0.580710	-2.700462	-1.562550
H	-1.419689	-5.008214	-1.272938
H	-2.976822	-5.545956	0.585662
H	-3.689931	-3.748758	2.143503
H	-2.894647	-1.437431	1.821632
C	2.850991	-0.194850	0.018871
C	4.078041	-0.120531	0.052763
C	5.496214	-0.041132	0.092741
C	6.290346	-1.189284	-0.090138
C	7.675411	-1.109652	-0.051608
C	8.305839	0.112434	0.170756
C	7.534659	1.257822	0.354112
C	6.149045	1.186184	0.315747
H	5.802387	-2.143069	-0.262809
H	8.267681	-2.007821	-0.195631
H	9.388688	0.171701	0.200802
H	8.016728	2.214701	0.527973
H	5.551443	2.080703	0.458565

P4b, G°=-1269.013451 au

C	-1.789112	-1.791915	-0.148987
C	-2.804496	-2.209088	0.734223
C	-3.236310	-3.534359	0.744233
C	-2.670582	-4.466046	-0.115939
C	-1.662070	-4.069694	-0.993977
C	-1.215441	-2.757898	-0.993535
C	-3.437566	-1.169897	1.621179
H	-4.018343	-3.832885	1.436738
H	-3.006016	-5.497437	-0.096684
H	-1.213794	-4.791780	-1.668402
H	-0.409741	-2.451411	-1.655165
H	-2.671882	-0.585179	2.145269
H	-4.099047	-1.622608	2.359581
C	-4.226356	-0.285462	0.725866
C	-5.613187	-0.346304	0.630040
C	-6.297986	0.456699	-0.260950
C	-5.575452	1.321508	-1.083567
C	-4.210107	1.356393	-0.977797
N	-3.540357	0.569154	-0.088001
H	-6.136433	-1.033351	1.283318
H	-7.378885	0.414674	-0.319463
H	-6.063312	1.961482	-1.806278
H	-3.587857	1.997419	-1.587047
Au	0.805443	-0.265510	-0.104188
C	-1.266804	-0.418633	-0.130190
C	-2.073172	0.671509	-0.056112
C	-1.627575	2.079734	0.003246
C	-2.223866	2.963246	0.914489
C	-1.795318	4.280568	1.012527
C	-0.772717	4.745981	0.190261
C	-0.187953	3.885319	-0.734108
C	-0.613347	2.565676	-0.830900
H	-3.018066	2.607366	1.565756
H	-2.257499	4.943802	1.736146
H	-0.439720	5.775803	0.263919
H	0.595424	4.245231	-1.392915
H	-0.171099	1.900765	-1.565876
C	2.801075	-0.197791	-0.015973
C	4.029170	-0.166067	0.039656
C	5.448673	-0.137244	0.099236
C	6.188544	-1.320476	0.285968
C	7.575008	-1.291387	0.341077
C	8.260966	-0.085824	0.212224
C	7.543901	1.094028	0.027615
C	6.157177	1.072551	-0.028562
H	5.657280	-2.261372	0.386496

H	8.124833	-2.216083	0.485560
H	9.344810	-0.065984	0.255386
H	8.069407	2.038385	-0.073806
H	5.601888	1.993636	-0.173138

TS0, G°=-1881.786197 au

C	0.554841	1.559504	-0.231335
C	1.811751	2.112280	-0.466844
C	1.962950	3.491003	-0.285194
C	0.889488	4.281263	0.107529
C	-0.356430	3.703209	0.327091
C	-0.531093	2.331317	0.153436
C	2.979696	1.253863	-0.886655
H	2.936708	3.939247	-0.463131
H	1.026877	5.348895	0.242477
H	-1.199431	4.313247	0.636130
H	-1.497073	1.868738	0.324668
H	3.865254	1.868655	-1.052968
H	2.749565	0.757915	-1.839641
C	3.287153	0.195665	0.129900
C	4.536668	0.054752	0.725286
C	4.735609	-0.939285	1.672918
C	3.677464	-1.768783	2.023810
C	2.452361	-1.578171	1.409202
N	2.284236	-0.627451	0.481237
H	5.336845	0.726986	0.439558
H	5.706679	-1.058767	2.140410
H	3.787972	-2.547661	2.767486
H	1.562906	-2.149170	1.653186
Au	0.359565	-0.444689	-0.486482
Cl	0.293436	-2.931880	-0.896431
Cl	-1.368303	-0.056328	-2.125352
C	-0.708821	-0.742620	1.893056
C	-1.905497	-0.546439	1.600965
C	-3.257205	-0.321369	1.224998
C	-3.936646	0.859258	1.586869
C	-5.245636	1.084822	1.181155
C	-5.915357	0.142788	0.403533
C	-5.259409	-1.031229	0.035375
C	-3.952924	-1.262778	0.439429
H	-3.417432	1.597251	2.191732
H	-5.747180	2.002735	1.472537
H	-6.937444	0.321537	0.086128
H	-5.771469	-1.769914	-0.573937
H	-3.439141	-2.171864	0.141961

TS00, G°=-1729.296085 au

C	0.743568	-1.511591	1.181828
C	0.475148	-2.881856	1.195517
C	0.671138	-3.585393	2.387816
C	1.135087	-2.942590	3.529182
C	1.401129	-1.577916	3.494455
C	1.209599	-0.858599	2.316772
C	-0.043237	-3.584175	-0.033423
H	0.459317	-4.651125	2.409106
H	1.286493	-3.506333	4.443675
H	1.759863	-1.065971	4.381816
H	1.415970	0.205418	2.287219
H	-0.132613	-4.656385	0.147690
H	0.671885	-3.453738	-0.857347
C	-1.368839	-3.042733	-0.483008
C	-2.493640	-3.847847	-0.629983
C	-3.688238	-3.286429	-1.057483
C	-3.737321	-1.924946	-1.329923
C	-2.589447	-1.171277	-1.160118
N	-1.438284	-1.723458	-0.744535
H	-2.418480	-4.906056	-0.409806
H	-4.571025	-3.905175	-1.175174
H	-4.648633	-1.445415	-1.664823

H	-2.555731	-0.100091	-1.326910
Au	0.341391	-0.507761	-0.552450
Cl	-0.070621	0.383700	-2.894466
C	2.114887	0.339468	-0.444185
C	-1.360210	1.350728	0.441561
C	-2.431118	1.967827	0.572636
C	-3.668349	2.657061	0.719815
C	-4.802331	2.286014	-0.031796
C	-6.010549	2.954591	0.111153
C	-6.130122	4.015365	1.006812
C	-5.021285	4.398400	1.758615
C	-3.810848	3.732711	1.619726
H	-4.717136	1.461401	-0.733494
H	-6.866708	2.646448	-0.481736
H	-7.075061	4.537142	1.116840
H	-5.100844	5.224027	2.459636
H	-2.951473	4.036834	2.209490
C	3.206936	0.874579	-0.347694
C	4.470869	1.512569	-0.224443
C	5.203111	1.421125	0.972894
C	6.436583	2.045338	1.096577
C	6.967937	2.772009	0.033640
C	6.253144	2.870143	-1.157767
C	5.018684	2.249395	-1.289299
H	4.788265	0.855291	1.801193
H	6.986990	1.964809	2.028499
H	7.932312	3.258845	0.133396
H	6.659990	3.435100	-1.990332
H	4.461576	2.327555	-2.217007

TS1’, G°=-1729.295487 au

C	0.097925	1.169888	0.260836
C	-0.570961	1.486541	1.444351
C	-0.890997	2.829415	1.679086
C	-0.534783	3.823555	0.777274
C	0.147608	3.485478	-0.387857
C	0.468002	2.155180	-0.646290
C	-0.971101	0.428133	2.439584
H	-1.420444	3.085673	2.593335
H	-0.786644	4.858505	0.983885
H	0.431702	4.252689	-1.101244
H	0.992013	1.887594	-1.556766
H	-1.363508	0.895040	3.344727
H	-0.084906	-0.152309	2.729066
C	-1.987859	-0.528924	1.883301
C	-3.279286	-0.634094	2.396514
C	-4.155075	-1.557941	1.844499
C	-3.722176	-2.357706	0.792556
C	-2.433745	-2.181145	0.315756
N	-1.594274	-1.286950	0.849829
H	-3.580231	0.002308	3.221207
H	-5.164136	-1.653780	2.231243
H	-4.371742	-3.095786	0.337922
H	-2.038397	-2.748068	-0.520845
Au	0.448667	-0.796228	-0.149495
Cl	0.825179	-3.277752	-0.476340
C	2.382416	-0.387530	-0.066689
C	-1.093921	-0.581902	-2.062223
C	-2.187742	-0.006898	-1.933127
C	-3.409836	0.629700	-1.591368
C	-3.391920	1.887209	-0.953183
C	-4.566815	2.481867	-0.515505
C	-5.793925	1.850441	-0.714392
C	-5.831944	0.615837	-1.359990
C	-4.659168	0.010595	-1.791470
H	-2.434787	2.374810	-0.792530
H	-4.525471	3.445035	-0.014541
H	-6.711976	2.317126	-0.372413
H	-6.783479	0.118053	-1.521280

H	-4.691342	-0.960510	-2.276549
C	3.567093	-0.089885	-0.069314
C	4.944476	0.258658	-0.079708
C	5.337635	1.607995	-0.145417
C	6.681574	1.954090	-0.157043
C	7.663734	0.967755	-0.103325
C	7.288539	-0.372073	-0.038110
C	5.946575	-0.726671	-0.026113
H	4.572555	2.376811	-0.188116
H	6.964794	3.000556	-0.209066
H	8.713556	1.241121	-0.112994
H	8.047329	-1.146987	0.003439
H	5.656323	-1.770851	0.024777

TS2’, G°=-1729.320201 au

C	1.025428	1.176535	-0.835798
C	0.953560	2.139324	0.190977
C	1.049098	3.482717	-0.172603
C	1.201696	3.878564	-1.498245
C	1.267496	2.912685	-2.498130
C	1.180717	1.565971	-2.171976
C	0.793477	1.746476	1.635246
H	1.004410	4.232360	0.613840
H	1.273833	4.931958	-1.746123
H	1.388580	3.202863	-3.536893
H	1.245900	0.810842	-2.947354
H	1.065528	2.601851	2.263908
H	1.483238	0.932466	1.879267
C	-0.599379	1.288449	2.001817
C	-1.707016	2.106428	1.767185
C	-2.968894	1.650650	2.114330
C	-3.091051	0.386427	2.680057
C	-1.934979	-0.354103	2.879922
N	-0.708224	0.074303	2.560169
H	-1.570810	3.082740	1.311073
H	-3.845983	2.265514	1.938471
H	-4.059700	-0.020188	2.949764
H	-1.990070	-1.350403	3.315367
Au	0.159031	-0.722040	-0.501253
Cl	0.114167	-3.287536	0.147401
C	2.125629	-0.338040	-0.366437
C	-1.872303	-0.662721	-0.705749
C	3.339544	-0.393054	-0.176156
C	4.729650	-0.420640	0.064031
C	5.638692	-0.766170	-0.955304
C	7.002659	-0.781843	-0.705551
C	7.492873	-0.455160	0.557274
C	6.604609	-0.111210	1.574513
C	5.238775	-0.093515	1.337040
H	5.257014	-1.021281	-1.938110
H	7.689448	-1.051035	-1.501371
H	8.560660	-0.469118	0.747799
H	6.980146	0.144014	2.560077
H	4.545672	0.172900	2.128357
C	-3.093778	-0.559706	-0.715066
C	-4.506614	-0.415650	-0.674352
C	-5.099904	0.858863	-0.746918
C	-6.478468	1.006957	-0.681872
C	-7.301423	-0.108892	-0.544745
C	-6.729991	-1.377182	-0.473288
C	-5.351617	-1.532198	-0.536410
H	-4.459676	1.729302	-0.851599
H	-6.914610	1.999427	-0.738930
H	-8.378759	0.009077	-0.494296
H	-7.363153	-2.252399	-0.366409
H	-4.909339	-2.521520	-0.477615

TS3’, G°=-1729.380236 au

C	0.370772	2.340917	-1.049750

C	0.153554	3.222394	0.033614
C	-0.791769	4.234588	-0.117510
C	-1.509095	4.388835	-1.299725
C	-1.288422	3.518173	-2.363407
C	-0.357373	2.498955	-2.237819
C	0.916292	3.055732	1.319738
H	-0.965268	4.913819	0.712574
H	-2.239945	5.185504	-1.389014
H	-1.844569	3.629977	-3.287768
H	-0.186058	1.804658	-3.053826
H	0.709095	3.914243	1.968876
H	1.994771	3.059705	1.131308
C	0.594228	1.779933	2.063597
C	-0.726604	1.364460	2.240880
C	-0.977642	0.180098	2.917887
C	0.098078	-0.558803	3.395629
C	1.377512	-0.062944	3.179627
N	1.635846	1.080084	2.536196
H	-1.540739	1.954161	1.828757
H	-1.995740	-0.170933	3.052863
H	-0.046760	-1.499936	3.913752
H	2.247206	-0.616801	3.530516
Au	0.207502	-0.809634	-0.490963
Cl	1.956246	-2.851433	0.141420
C	1.339435	1.298726	-0.925515
C	-1.759188	-1.012447	-0.471031
C	2.344141	0.603934	-0.784561
C	3.618646	-0.006350	-0.645568
C	4.301148	0.082403	0.578829
C	5.552922	-0.500608	0.715446
C	6.136629	-1.176512	-0.353750
C	5.461742	-1.267488	-1.569232
C	4.209518	-0.690753	-1.718536
H	3.823150	0.600167	1.405538
H	6.076143	-0.430487	1.663601
H	7.114463	-1.632739	-0.240199
H	5.912195	-1.795754	-2.403002
H	3.672847	-0.771379	-2.657965
C	-2.982560	-1.063992	-0.376856
C	-4.394460	-1.104127	-0.230610
C	-5.030491	-0.351166	0.775920
C	-6.409517	-0.386226	0.927518
C	-7.192881	-1.168710	0.081844
C	-6.579483	-1.918078	-0.919496
C	-5.200448	-1.889461	-1.076306
H	-4.422684	0.261829	1.434638
H	-6.877297	0.202005	1.710908
H	-8.270787	-1.194090	0.202088
H	-7.180674	-2.531315	-1.583560
H	-4.727272	-2.476667	-1.856664

TS1, G°=-1576.784217 au

C	0.287505	-2.117551	0.185573
C	1.031753	-2.494004	1.310606
C	1.563875	-3.785928	1.384474
C	1.347015	-4.707531	0.367088
C	0.590481	-4.338043	-0.741594
C	0.063596	-3.051980	-0.825664
C	1.277845	-1.517141	2.435750
H	2.145786	-4.068275	2.258954
H	1.761101	-5.708095	0.441955
H	0.410873	-5.049605	-1.542344
H	-0.520890	-2.768238	-1.695779
H	1.758738	-2.020795	3.277012
H	0.315124	-1.129114	2.795425
C	2.114496	-0.343481	2.014483
C	3.383685	-0.103679	2.540076
C	4.095635	1.011253	2.122901
C	3.522347	1.869747	1.191272

C	2.267988	1.560086	0.692745
N	1.588947	0.477942	1.092196
H	3.794876	-0.790292	3.271614
H	5.084702	1.210612	2.521853
H	4.039422	2.755641	0.843098
H	1.775290	2.169556	-0.058146
Au	-0.368517	-0.157397	0.030236
C	-2.218796	-0.858806	-0.017722
C	-0.971622	1.818650	-0.043418
C	1.230296	0.040784	-1.884661
C	-3.348537	-1.316509	-0.092155
C	-4.655649	-1.867704	-0.176512
C	-4.839971	-3.253930	-0.334380
C	-6.115584	-3.795032	-0.414108
C	-7.236695	-2.971576	-0.338751
C	-7.069623	-1.597500	-0.183147
C	-5.797157	-1.048749	-0.103142
H	-3.966808	-3.896314	-0.393298
H	-6.236244	-4.866826	-0.535939
H	-8.232559	-3.397484	-0.400949
H	-7.937508	-0.948369	-0.123926
H	-5.669590	0.022044	0.017891
C	-1.278032	3.004795	-0.063121
C	-1.587707	4.392395	-0.083805
C	-0.637506	5.345513	0.328056
C	-0.935394	6.701002	0.307867
C	-2.184487	7.142047	-0.123289
C	-3.135204	6.210942	-0.534653
C	-2.844573	4.853526	-0.516142
H	0.336964	5.003461	0.663141
H	-0.187528	7.418872	0.629863
H	-2.414771	8.202132	-0.138733
H	-4.111176	6.544868	-0.872720
H	-3.587704	4.130985	-0.837475
C	2.458109	-0.139755	-1.884588
C	3.853941	-0.335880	-1.714260
C	4.357205	-1.579646	-1.279438
C	5.710054	-1.752395	-1.021450
C	6.603282	-0.696471	-1.196600
C	6.126556	0.536177	-1.638589
C	4.774096	0.716352	-1.893764
H	3.661924	-2.401963	-1.135986
H	6.072085	-2.717272	-0.679142
H	7.660304	-0.834098	-0.993618
H	6.814535	1.364431	-1.779937
H	4.403905	1.683043	-2.222456

TS2, G°=-1576.801939 au

C	-0.670424	-2.002853	-0.697208
C	-0.342755	-2.843812	0.396896
C	-0.106462	-4.195070	0.156530
C	-0.161827	-4.735364	-1.126650
C	-0.468579	-3.905146	-2.203255
C	-0.719444	-2.558593	-1.991978
C	-0.261658	-2.297509	1.797340
H	0.128996	-4.838612	1.001539
H	0.027688	-5.792108	-1.282137
H	-0.514256	-4.308622	-3.210255
H	-0.974743	-1.917952	-2.830679
H	-0.368573	-3.126208	2.507306
H	-1.091141	-1.606814	1.978070
C	1.020267	-1.557096	2.104028
C	2.263087	-2.174170	1.939036
C	3.415414	-1.467435	2.241769
C	3.296193	-0.158068	2.695308
C	2.021968	0.374170	2.824799
N	0.898567	-0.298321	2.547568
H	2.314017	-3.193310	1.566275
H	4.393954	-1.920535	2.111476

H	4.170084	0.439361	2.930611
H	1.888509	1.398271	3.169996
Au	-0.236680	0.092881	-0.588130
C	-2.025920	-0.817913	-0.367881
C	-0.511396	2.144137	-0.405930
C	1.824823	0.101009	-0.850592
C	-3.223860	-1.025452	-0.143733
C	-4.565302	-1.342388	0.131779
C	-4.976682	-1.643044	1.449560
C	-6.298366	-1.956772	1.724506
C	-7.248818	-1.981681	0.704610
C	-6.858338	-1.687515	-0.601022
C	-5.539689	-1.372089	-0.890036
H	-4.239675	-1.624622	2.246221
H	-6.591600	-2.184657	2.744585
H	-8.282196	-2.226840	0.924966
H	-7.590530	-1.704083	-1.402343
H	-5.241629	-1.142617	-1.907787
C	-0.654429	3.359006	-0.301892
C	-0.815842	4.766346	-0.178490
C	-0.507942	5.428425	1.025009
C	-0.664560	6.802742	1.141576
C	-1.129925	7.554558	0.065239
C	-1.438846	6.914290	-1.132601
C	-1.285901	5.540107	-1.256285
H	-0.143791	4.846743	1.865767
H	-0.420646	7.291311	2.079826
H	-1.250600	8.628653	0.159009
H	-1.802231	7.490257	-1.978020
H	-1.527926	5.045673	-2.191556
C	3.042671	-0.049125	-0.853448
C	4.439776	-0.295988	-0.777905
C	4.973013	-1.530580	-1.193491
C	6.327863	-1.802589	-1.060887
C	7.186722	-0.853329	-0.511147
C	6.675772	0.376194	-0.100316
C	5.322403	0.655087	-0.233623
H	4.302563	-2.276672	-1.609179
H	6.716342	-2.763457	-1.384079
H	8.244613	-1.069360	-0.404217
H	7.337432	1.122405	0.328683
H	4.925025	1.609479	0.097558

TS3, G°=-1576.860265 au

C	-2.083484	1.808348	-0.619827
C	-2.418328	2.554794	0.536257
C	-3.760088	2.846685	0.774756
C	-4.761825	2.430246	-0.097393
C	-4.428152	1.691193	-1.228589
C	-3.102370	1.371938	-1.479722
C	-1.345453	3.012166	1.487064
H	-4.019216	3.415620	1.663842
H	-5.798830	2.671681	0.112779
H	-5.203356	1.346518	-1.905327
H	-2.834067	0.772936	-2.343015
H	-1.805525	3.610143	2.283013
H	-0.637673	3.677534	0.979264
C	-0.531405	1.899951	2.107438
C	-1.111791	0.676410	2.445563
C	-0.313283	-0.329161	2.970150
C	1.042918	-0.082742	3.148483
C	1.526556	1.173872	2.805473
N	0.771627	2.153714	2.299570
H	-2.169760	0.509233	2.267618
H	-0.740227	-1.295878	3.218242
H	1.712218	-0.844154	3.533638
H	2.583059	1.406636	2.934832
Au	0.134679	-0.655405	-0.676464
C	-0.713908	1.492819	-0.867118

C	2.016409	-1.454909	-0.518275
C	-1.734482	-1.541255	-0.513850
C	0.536394	1.577900	-0.926505
C	1.745255	2.347459	-1.034928
C	1.646428	3.714663	-1.353227
C	2.784229	4.505264	-1.424363
C	4.039580	3.952701	-1.176385
C	4.146494	2.601159	-0.860148
C	3.012604	1.800857	-0.793054
H	0.666342	4.142884	-1.541340
H	2.692128	5.557989	-1.671796
H	4.927832	4.573565	-1.230671
H	5.121572	2.164826	-0.665497
H	3.086784	0.745645	-0.551094
C	3.163893	-1.844197	-0.301807
C	4.511351	-2.186358	-0.015176
C	5.188717	-3.205239	-0.711898
C	6.510967	-3.510856	-0.418827
C	7.194593	-2.815347	0.576072
C	6.539146	-1.804588	1.276531
C	5.218671	-1.490916	0.986852
H	4.661149	-3.751992	-1.486994
H	7.012347	-4.300136	-0.970583
H	8.227178	-3.058485	0.803404
H	7.062298	-1.255345	2.053344
H	4.709812	-0.700349	1.531644
C	-2.912702	-1.863375	-0.361222
C	-4.290547	-2.144700	-0.163218
C	-5.081237	-1.282782	0.623321
C	-6.432704	-1.531282	0.816789
C	-7.036075	-2.644231	0.233822
C	-6.269186	-3.507227	-0.545848
C	-4.916010	-3.264993	-0.742675
H	-4.614456	-0.408685	1.067373
H	-7.021076	-0.850420	1.424843
H	-8.093148	-2.836509	0.385146
H	-6.729594	-4.377020	-1.004444
H	-4.323866	-3.940853	-1.351627

TS1'', G°=-1421.427564 au

C	0.043044	1.179730	-0.585794
C	-0.580233	1.980542	0.389141
C	-0.685331	3.350387	0.139609
C	-0.211021	3.909982	-1.039978
C	0.345004	3.091515	-2.020799
C	0.466971	1.727426	-1.802439
C	-1.284648	1.376616	1.576205
H	-1.173253	3.977198	0.881105
H	-0.294630	4.978683	-1.203581
H	0.695808	3.515222	-2.955652
H	0.921563	1.088772	-2.550396
H	-1.431209	2.131814	2.350322
H	-0.677885	0.577023	2.018673
C	-2.637823	0.804503	1.198441
C	-3.815829	1.293933	1.752830
C	-5.031449	0.734673	1.380303
C	-5.046733	-0.310651	0.465813
C	-3.838250	-0.755504	-0.045462
N	-2.671295	-0.203986	0.305930
H	-3.770921	2.102176	2.473787
H	-5.956616	1.110065	1.803457
H	-5.972336	-0.775893	0.150965
H	-3.778878	-1.578078	-0.751302
Au	-0.587458	-0.796763	-0.400463
Cl	-1.230186	-3.184405	-0.418413
C	1.316763	-0.264240	-0.245631
C	2.512518	-0.232580	0.024618
C	3.889934	-0.192036	0.337255
C	4.856383	-0.087561	-0.681197

C	6.206089	-0.042573	-0.365216
C	6.621334	-0.101466	0.963247
C	5.673982	-0.205617	1.979560
C	4.321794	-0.250846	1.676034
H	4.531864	-0.042886	-1.715288
H	6.939591	0.038190	-1.160568
H	7.678100	-0.067228	1.205296
H	5.991933	-0.252595	3.015809
H	3.583289	-0.332754	2.466192

TS2'', G°=-1421.494801 au

C	-1.054775	1.669217	-0.981706
C	-1.980797	2.081009	0.002034
C	-3.163584	2.683424	-0.424312
C	-3.441065	2.864082	-1.774386
C	-2.530759	2.439684	-2.738846
C	-1.344061	1.844565	-2.344876
C	-1.736190	1.832345	1.469358
H	-3.879177	3.018071	0.321021
H	-4.369966	3.337193	-2.074198
H	-2.743644	2.576420	-3.792977
H	-0.620522	1.512172	-3.081430
H	-2.274649	2.584423	2.052284
H	-0.672726	1.922608	1.703723
C	-2.201262	0.450429	1.870741
C	-3.548046	0.204398	2.148324
C	-3.946234	-1.084559	2.470832
C	-2.988939	-2.092802	2.502148
C	-1.674056	-1.754618	2.213241
N	-1.273298	-0.515082	1.907740
H	-4.267165	1.016796	2.114235
H	-4.985820	-1.299522	2.695031
H	-3.252797	-3.115209	2.746790
H	-0.894404	-2.514201	2.226327
Au	0.523364	-1.052464	-0.760894
Cl	0.166958	-3.317963	-1.201439
C	0.193893	1.090146	-0.598008
C	1.340550	0.809846	-0.175431
C	2.631397	1.063004	0.393860
C	2.899584	2.346101	0.899755
C	4.136796	2.624147	1.460612
C	5.117384	1.636244	1.524600
C	4.855897	0.363173	1.025875
C	3.619826	0.073009	0.465267
H	2.131763	3.110917	0.847259
H	4.337423	3.616327	1.850162
H	6.083511	1.859187	1.964273
H	5.616738	-0.407953	1.075769
H	3.407136	-0.918669	0.078133

TS3a, G°=-1421.484941 au

C	-0.613901	-1.867834	0.225985
C	-1.609459	-2.511182	-0.543525
C	-1.742282	-3.892714	-0.443584
C	-0.910354	-4.637519	0.384938
C	0.064462	-4.001085	1.149745
C	0.205677	-2.625591	1.082208
C	-2.535867	-1.700082	-1.399874
H	-2.508349	-4.389364	-1.030890
H	-1.026107	-5.714564	0.437849
H	0.712956	-4.576854	1.800382
H	0.962355	-2.115366	1.669666
H	-3.184743	-2.348360	-1.989947
H	-1.949791	-1.097151	-2.108150
C	-3.359639	-0.752090	-0.565694
C	-4.742830	-0.635477	-0.675630
C	-5.403488	0.270960	0.143541
C	-4.672245	1.031457	1.049634
C	-3.296388	0.856104	1.096480

N	-2.668797	-0.015175	0.306357
H	-5.283647	-1.246448	-1.389557
H	-6.480518	0.380761	0.078249
H	-5.153301	1.745727	1.706639
H	-2.672333	1.428389	1.778200
Au	2.118372	0.047013	-0.096119
Cl	4.427514	-0.413841	-0.239239
C	-0.436486	-0.458462	0.135454
C	0.159317	0.665159	0.019800
C	-0.205947	2.074067	-0.011952
C	-1.237791	2.517702	-0.849957
C	-1.599528	3.858537	-0.861731
C	-0.943895	4.771036	-0.039632
C	0.093451	4.339086	0.782891
C	0.473031	3.003701	0.785888
H	-1.745080	1.807215	-1.493916
H	-2.397427	4.191866	-1.517093
H	-1.231965	5.816793	-0.048821
H	0.615522	5.046665	1.418179
H	1.289859	2.663611	1.415233

TS3b, G°=-1421.48388 au

C	0.586431	-1.912590	-0.089962
C	1.789083	-2.238493	0.572065
C	2.313816	-3.521251	0.416199
C	1.677592	-4.477638	-0.366051
C	0.480774	-4.162316	-1.001487
C	-0.063590	-2.896494	-0.849378
C	2.518904	-1.238711	1.433099
H	3.239885	-3.769903	0.927203
H	2.108471	-5.467863	-0.467897
H	-0.032991	-4.902708	-1.604710
H	-1.004640	-2.641880	-1.328155
H	3.181423	-1.765226	2.122534
H	1.803386	-0.663309	2.031012
C	3.317243	-0.284136	0.594041
C	4.703940	-0.360181	0.470603
C	5.360182	0.542484	-0.354604
C	4.620135	1.500627	-1.039116
C	3.244354	1.512667	-0.864640
N	2.616849	0.642532	-0.072650
H	5.250733	-1.118869	1.019866
H	6.438755	0.501530	-0.460964
H	5.094572	2.224509	-1.690280
H	2.614218	2.246303	-1.364566
Au	-2.055894	-0.305797	0.065669
Cl	-4.413701	-0.221155	0.124959
C	-0.013371	-0.590119	0.010194
C	0.367445	0.630164	0.054702
C	0.233890	2.051440	0.040647
C	0.745814	2.828586	1.092098
C	0.635027	4.209252	1.052789
C	0.017727	4.832168	-0.030702
C	-0.490593	4.069370	-1.078988
C	-0.381080	2.686825	-1.051430
H	1.235135	2.334955	1.925066
H	1.030491	4.804034	1.868695
H	-0.066186	5.913231	-0.057483
H	-0.970141	4.553186	-1.922643
H	-0.774876	2.081944	-1.861719

TS1’’’, G°=-1268.922978 au

C	1.644965	1.267368	-0.675200
C	1.759614	2.335875	0.229662
C	2.563156	3.420703	-0.128058
C	3.228725	3.454523	-1.347222
C	3.071967	2.407059	-2.252325
C	2.284629	1.314011	-1.918873
C	0.908283	2.406802	1.469225

H	2.647031	4.257677	0.560317
H	3.857210	4.302410	-1.597185
H	3.572175	2.433747	-3.214607
H	2.181340	0.482824	-2.607395
H	1.343228	3.099392	2.192016
H	0.856735	1.423935	1.953925
C	-0.500855	2.857712	1.146763
C	-1.044603	4.013927	1.699947
C	-2.345227	4.377782	1.376471
C	-3.080727	3.574074	0.514102
C	-2.478187	2.434447	0.004105
N	-1.220430	2.098055	0.304003
H	-0.450009	4.612832	2.380631
H	-2.780912	5.277254	1.797564
H	-4.098894	3.820370	0.239153
H	-3.004995	1.755436	-0.660860
Au	-0.083682	0.116026	-0.364689
C	1.729200	-0.671576	-0.167298
C	2.705090	-1.361736	0.099941
C	3.829766	-2.162344	0.407723
C	4.566428	-2.789003	-0.615563
C	5.672923	-3.567978	-0.310478
C	6.070631	-3.741795	1.013210
C	5.349580	-3.126993	2.034483
C	4.241638	-2.345568	1.741446
H	4.257132	-2.655048	-1.646755
H	6.229110	-4.043592	-1.111729
H	6.936052	-4.352320	1.247397
H	5.653051	-3.257589	3.068125
H	3.681075	-1.867694	2.537803
C	-1.827728	-0.946137	-0.258934
C	-2.931550	-1.476504	-0.195415
C	-4.224471	-2.063488	-0.120603
C	-5.342253	-1.286002	0.234059
C	-6.605988	-1.854590	0.307910
C	-6.785811	-3.208107	0.032025
C	-5.688433	-3.990259	-0.319809
C	-4.421485	-3.428384	-0.396862
H	-5.201927	-0.231487	0.450430
H	-7.456188	-1.238698	0.582932
H	-7.774680	-3.650312	0.091106
H	-5.820788	-5.045534	-0.535783
H	-3.567560	-4.038964	-0.671244

TS2’’’, G°=-1269.000205 au

C	-2.360909	-1.196001	-1.302827
C	-2.964903	-2.120530	-0.422531
C	-3.327145	-3.367595	-0.929473
C	-3.091737	-3.708738	-2.256333
C	-2.480397	-2.798033	-3.114209
C	-2.116989	-1.548468	-2.640591
C	-3.164239	-1.803778	1.038339
H	-3.805004	-4.083021	-0.266505
H	-3.386114	-4.686843	-2.621382
H	-2.292558	-3.059638	-4.149389
H	-1.645150	-0.824026	-3.295739
H	-4.011516	-2.382767	1.415576
H	-3.391410	-0.744116	1.176511
C	-1.925069	-2.136342	1.838624
C	-1.675850	-3.447128	2.252838
C	-0.507842	-3.725060	2.947116
C	0.381015	-2.686659	3.203247
C	0.048885	-1.415968	2.753752
N	-1.077030	-1.130225	2.089642
H	-2.393092	-4.232095	2.034066
H	-0.294123	-4.734151	3.283732
H	1.307530	-2.855361	3.739859
H	0.718818	-0.576887	2.933420
Au	0.156156	0.531565	-0.519574

C	-2.015486	0.111177	-0.844375
C	-1.901148	1.277684	-0.427200
C	-2.273033	2.584094	0.027174
C	-3.616188	2.818845	0.366665
C	-4.009856	4.072223	0.811108
C	-3.079081	5.102837	0.921822
C	-1.747018	4.875474	0.587036
C	-1.341352	3.624057	0.144885
H	-4.336821	2.012833	0.277324
H	-5.048184	4.245847	1.072339
H	-3.392123	6.081196	1.270103
H	-1.020004	5.675586	0.673190
H	-0.303742	3.436286	-0.113223
C	2.116499	0.362021	-0.418356
C	3.336505	0.267210	-0.338450
C	4.749827	0.156525	-0.246632
C	5.458205	-0.742396	-1.064634
C	6.838915	-0.846647	-0.970045
C	7.545221	-0.058487	-0.064639
C	6.856582	0.836837	0.750158
C	5.475626	0.944935	0.664903
H	4.910001	-1.355681	-1.772074
H	7.367750	-1.546732	-1.608753
H	8.624569	-0.141467	0.005445
H	7.399127	1.454758	1.458365
H	4.940692	1.641656	1.301742

TS4a, G°=-1268.989471 au

C	1.663115	-1.896947	-0.250588
C	2.564657	-2.648713	0.535620
C	2.541633	-4.036574	0.437511
C	1.646204	-4.683487	-0.407280
C	0.765045	-3.941091	-1.189094
C	0.780133	-2.557440	-1.122226
C	3.553617	-1.948994	1.421998
H	3.234965	-4.616334	1.039021
H	1.640967	-5.766764	-0.460194
H	0.069470	-4.439711	-1.854813
H	0.101044	-1.964808	-1.726845
H	4.140733	-2.670445	1.991650
H	3.015928	-1.325806	2.151272
C	4.458992	-1.042051	0.630338
C	5.843642	-1.011317	0.773791
C	6.576954	-0.128990	-0.009318
C	5.916038	0.693752	-0.915225
C	4.534221	0.603418	-0.998807
N	3.838570	-0.244850	-0.241366
H	6.330113	-1.669313	1.484948
H	7.656686	-0.086391	0.083464
H	6.455611	1.390908	-1.544433
H	3.960909	1.224269	-1.682158
Au	-0.973907	0.251988	-0.017182
C	1.642192	-0.472494	-0.160607
C	1.103680	0.678729	-0.057906
C	1.569005	2.055602	0.023119
C	2.588202	2.405192	0.920539
C	3.043111	3.715553	0.988498
C	2.495356	4.693823	0.162947
C	1.472838	4.357379	-0.720483
C	0.999749	3.053111	-0.779401
H	3.012920	1.643710	1.566483
H	3.829841	3.973505	1.689996
H	2.855783	5.715618	0.215792
H	1.034680	5.116305	-1.360136
H	0.193283	2.789495	-1.457148
C	-2.942484	0.083095	0.041993
C	-4.167597	0.017517	0.077270
C	-5.585990	-0.058015	0.117250
C	-6.373632	1.095313	-0.056587

C	-7.758917	1.020857	-0.017135
C	-8.393600	-0.200575	0.195838
C	-7.627495	-1.350751	0.369436
C	-6.241594	-1.284714	0.331333
H	-5.881607	2.048101	-0.222449
H	-8.347669	1.922409	-0.153475
H	-9.476622	-0.255674	0.226172
H	-8.113469	-2.306859	0.535830
H	-5.647326	-2.182421	0.466996

TS4b, G°=-1268.987154 au

C	1.744951	-1.904387	-0.092385
C	2.925160	-2.265767	0.592468
C	3.414777	-3.563924	0.454369
C	2.764303	-4.505300	-0.334963
C	1.590330	-4.156336	-0.995037
C	1.081431	-2.873501	-0.859596
C	3.667137	-1.278670	1.458630
H	4.324096	-3.836900	0.982912
H	3.166489	-5.508814	-0.423746
H	1.066399	-4.883988	-1.605230
H	0.157761	-2.592919	-1.357597
H	4.322527	-1.815844	2.146696
H	2.959163	-0.694461	2.056668
C	4.479291	-0.337273	0.619641
C	5.858850	-0.464069	0.460418
C	6.530851	0.423111	-0.368027
C	5.813302	1.418120	-1.023862
C	4.444504	1.482668	-0.816566
N	3.802560	0.628534	-0.017144
H	6.387835	-1.251243	0.986413
H	7.604179	0.341399	-0.500218
H	6.300148	2.130655	-1.678235
H	3.831083	2.244800	-1.293090
Au	-0.918513	-0.246704	0.012705
C	1.185901	-0.564283	-0.004199
C	1.628364	0.633361	0.061749
C	1.457829	2.056289	0.062782
C	2.017527	2.849295	1.076401
C	1.849348	4.225167	1.057730
C	1.126812	4.829537	0.030637
C	0.572078	4.051674	-0.982071
C	0.738572	2.674039	-0.973855
H	2.586727	2.372522	1.867497
H	2.281822	4.830159	1.847067
H	0.998450	5.906529	0.019228
H	0.012212	4.519440	-1.784441
H	0.310821	2.058638	-1.758826
C	-2.893685	-0.162409	0.035128
C	-4.120976	-0.143370	0.044392
C	-5.541788	-0.111667	0.054091
C	-6.268416	0.060876	-1.138650
C	-7.655934	0.090139	-1.126150
C	-8.353236	-0.051069	0.071350
C	-7.647963	-0.222341	1.260203
C	-6.260365	-0.252845	1.255731
H	-5.727372	0.171205	-2.072740
H	-8.197324	0.224198	-2.057236
H	-9.437819	-0.027818	0.078031
H	-8.183127	-0.333247	2.197906
H	-5.713182	-0.386840	2.183117
